# Reciprocity in dynamics of supramolecular biosystems for the clustering of ligands and receptors

**DOI:** 10.1073/pnas.2500686122

**Published:** 2025-09-08

**Authors:** Shikha Dhiman, Marle E. J. Vleugels, Richard A. J. Post, Martina Crippa, Annalisa Cardellini, Esmee de Korver, Lu Su, Anja R. A. Palmans, Giovanni M. Pavan, Remco W. van der Hofstad, Lorenzo Albertazzi, E. W. Meijer

**Affiliations:** ^a^Institute for Complex Molecular Systems, Eindhoven University of Technology, Eindhoven 5600 MB, The Netherlands; ^b^Laboratory of Macromolecular and Organic Chemistry, Eindhoven University of Technology, Eindhoven 5600 MB, The Netherlands; ^c^Department of Chemistry, Johannes Gutenberg University Mainz, Mainz D-55128, Germany; ^d^Department of Epidemiology and Biostatistics, Erasmus University Medical Center, Rotterdam 3015 GD, The Netherlands; ^e^Department of Applied Science and Technology, Politecnico di Torino, Torino 10129, Italy; ^f^Department of Innovative Technologies, University of Applied Sciences and Arts of Southern Switzerland, Polo Universitario Lugano, Lugano-Viganello 6962, Switzerland; ^g^Division of Biotherapeutics, Leiden Academic Centre for Drug Research, Leiden University, Leiden 2333 CC, The Netherlands; ^h^Department of Biomedical Engineering, Eindhoven University of Technology, Eindhoven 5600 MB, Netherlands; ^i^School of Chemistry and RNA Institute, University of New South Wales, Sydney, NSW 2052, Australia

**Keywords:** multivalency, supramolecular polymer, clustering, biomaterials, dynamics

## Abstract

Multivalent binding and the resulting dynamical clustering of receptors and ligands are known to be key features in biological interactions. For optimizing biomaterials capable of similar dynamical features, it is essential to understand the first step of these interactions, namely the multivalent molecular recognition between ligands and cell receptors. Here, we present the reciprocal cooperation between dynamic ligands in supramolecular polymers and dynamic receptors in model cell membranes, determining molecular recognition and multivalent binding via receptor clustering. The nonlinear dependences of the ligand concentration, receptors, and their binding affinity are observed experimentally by fluorescence and superresolution fluorescence microscopies, revealing a valency-dependent clustering mode of anchoring. The mechanism is supported by stochastic modeling demonstrating that such nonlinear dependence is unlikely in the absence of any dynamics and superselectivity. Using a coarse-grained molecular model, the subtle competition between local and global entropies that controls this anchoring mechanism explains the clustering. Further investigation using single particle tracking reveals the presence of two populations of bound and unbound receptors after the clustering process. The result of this study highlights the importance of reciprocity of dynamics in supramolecular polymer and lipid membrane for recruitment, multivalent binding, and clustering, all of which are crucial elements in the design of materials capable of actively interacting with biological targets.

## Significance

Supramolecular materials with dynamic and adaptive properties are promising for biomedical use. When synthetic materials contact living cells, their interaction is key for tissue development. Critical factors include biological recognition and mechanical properties during contact. The most crucial step is initial molecular recognition between biomaterial ligands and cell membrane receptors. Understanding this interaction is vital for designing effective biomaterials. Experiments with supramolecular polymers and supported bilayers show that receptor–ligand clustering occurs when both have similar dynamics. This clustering, confirmed by computational studies, is primarily entropy driven. These findings explain how superselectivity can arise during the early stages of interaction between supramolecular biomaterials and cells, guiding the design of next-generation bioactive materials for medical applications.

Developing biomaterials that interact in a controlled and effective way with cells and tissue is essential for numerous applications (1). Supramolecular biomaterials, in particular, have shown significant potential to influence cell fate within the realm of regenerative medicine (2, 3). Supramolecular polymeric materials are composed of distinct monomeric units held together by reversible noncovalent interactions, which make them dynamic, adaptive, and responsive (4–6). Through multicomponent self-assembly, they can be designed to present functional groups and exhibit modular physicochemical properties (7, 8). These features enable them to mimic the native microenvironment of extracellular matrices, making them promising candidates for biomedical applications (9–12). Understanding the interactions between supramolecular materials and cells, crucial for defining the chemical and physical features necessary for optimizing biomaterials, yet remains challenging due to the dynamic nature of both entities (13–16). The first point of interaction, molecular recognition of ligands by cell-surface receptors, is a fundamental biological process that remains poorly understood, especially in the context of multivalent and dynamic interactions between supramolecular materials and cells (17, 18). Most biomaterial studies emphasize ligand selectivity for target receptors, with less attention given to how the dynamics of both partners influence cooperativity and binding efficiency (19, 20).

Nature often employs multivalency to achieve strong molecular recognition by using multiple ligand/receptor pairs with individually weak binding (21–24). In many multivalent systems, high avidity primarily results from increased local concentration and a higher probability of rebinding, rather than changes in the intrinsic affinity of individual binding sites due to cooperativity. Superselectivity, characterized by a sharp transition between bound and unbound states over a narrow concentration range, arises when multiple weak binding sites are linked together (22–24). This phenomenon is largely driven by avidity, as multiple simultaneous interactions make complete dissociation less likely once initial binding occurs. While cooperativity can enhance this sharp transition, avidity—through local concentration effects and multivalent interactions—remains a key factor. Cooperativity arises when multiple binding events are interdependent, reshaping the system’s entropy and energy landscape. Multivalent cooperative binding occurs when both receptors and ligands possess multiple binding sites, where binding at one site influences the affinity of others. While multivalency alone enhances binding by increasing local effective concentration and rebinding probabilities, cooperativity adds complexity by altering intrinsic affinities upon initial binding. This interdependence of binding events can result in a significantly stronger overall binding than what would be expected from independent interactions.

One key feature of biological multivalency is receptor clustering, which plays a major role in cellular signaling, as seen in virus–cell interaction (25, 26) and immunological synapses (19, 27, 28). Receptor preclustering is a process driven by intracellular mechanisms such as cytoskeletal remodeling and lipid raft organization. This preclustering generates localized high-density receptor domains that enhance superselective binding. This enables immune cells to respond rapidly and selectively to ligands, even at low concentrations.

Inspired by these biological principles, chemists have leveraged multivalency for targeted drug design (29–32) and the control of supramolecular assembly (33–39). Recent studies by Bastings and coworkers demonstrated that even in low-valency regimes, the rigidity and spatial arrangement of ligands significantly influence superselective binding by reducing the entropic penalty of interaction. Here, binding efficacy depends not only on receptor density but also on ligand nano-organization and is important for preclustered receptors (40). In contrast, advancements in supramolecular chemistry have emphasized the important role of dynamics in rearranging monomer organization and influencing material function (41–45). The degree of internal dynamics in peptide amphiphile-based supramolecular matrices significantly impacts tissue regeneration and functional repair (46, 47). Membrane dynamics and receptor mobility are also key players in the molecular recognition process. Recently, Stupp and coworkers demonstrated that supramolecular dynamics are also an important factor in the receptor–ligand binding in synthetic materials (48). We also observed that ligand-appended supramolecular polymers interacting with the receptors on the surface of human erythrocytes (hRBCs) exhibit a clustering-based anchoring mechanism (49). These findings among others with biointerfaces highlight the pivotal role of reciprocity in dynamics in multivalency (50). However, despite these advances, we still lack a complete understanding of how ligand and receptor dynamics collectively influence multivalent binding. This gap in knowledge arises due to experimental limitations, as previous studies have primarily focused on either ligand dynamics or receptor dynamics separately, leaving the scenario where both partners are dynamic largely unexplored (51–55).

In this study, we investigated the scenario where both receptors coassembled in a membrane and ligands coassembled in a supramolecular polymer exhibit dynamic rearrangement capability, with exchange occurring within seconds. Through systematically varying i) monomer exchange dynamics within the supramolecular polymers, ii) lipid diffusion within the supportedlipid bilayers (SLB), and iii) the association/dissociation kinetics of the receptor–ligand pairs, we establish general guidelines for optimizing biomaterial design. Our findings highlight the critical role of the reciprocity of dynamics between supramolecular ligands and membrane-bound receptors in driving cooperative multivalent binding and receptor clustering. The dynamicity of ligands and receptors allows for continuous rearrangement, optimizing the spatial presentation of binding sites. This dynamic adaptation fosters cooperative binding, where initial binding events increase the likelihood of subsequent interactions, leading to strong multivalent attachment and receptor clustering. The reciprocal multivalent cooperative binding mechanism described in our study offers a highly adaptable approach for interacting also with preclustered receptors. Unlike static multivalent systems, dynamic supramolecular polymers can adapt to receptor clusters, ensuring optimal ligand–receptor engagement. Moreover, the supramolecular polymer’s ability to promote additional receptor clustering could further amplify cellular signaling, reinforcing immune cell activation and enhancing responses.

## Results and Discussion

### Design and Selection of the Components.

**Fig. 1.**
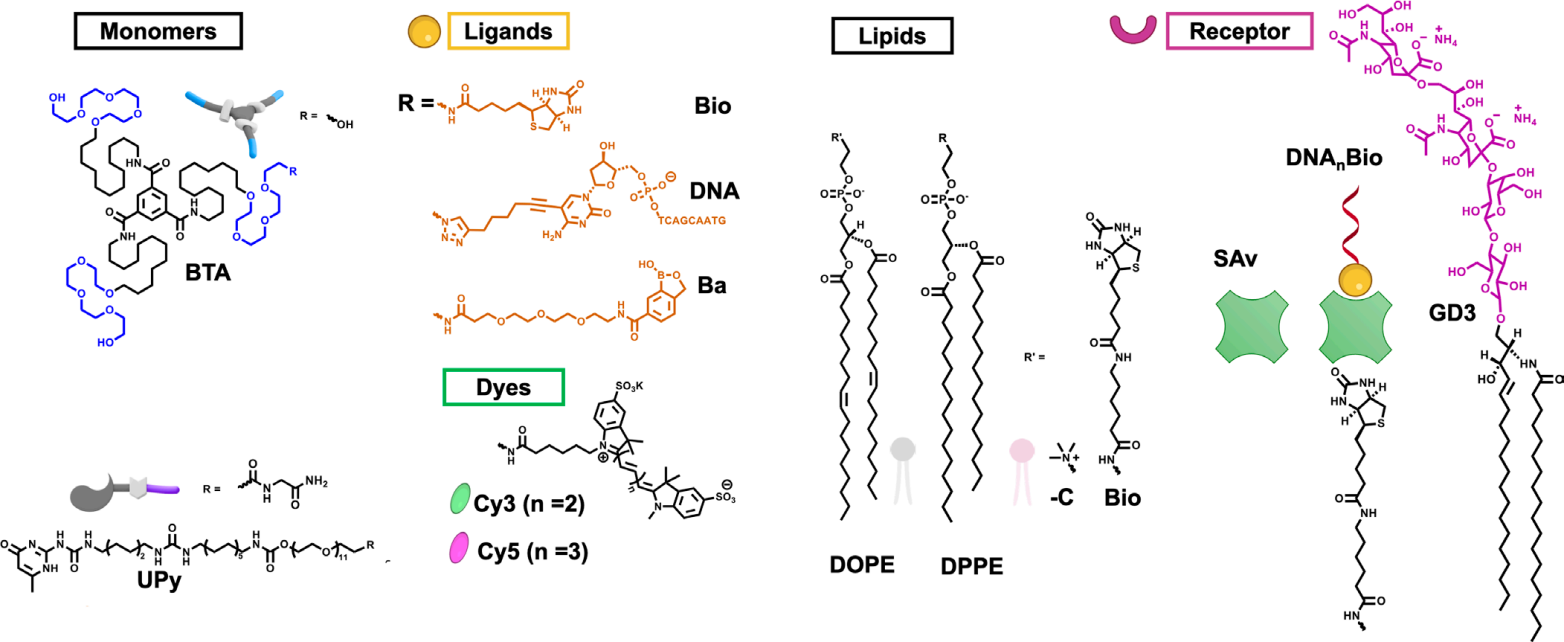
Chemical structures of monomers, ligands, lipids, and receptors used in this study. Schematics created with https://Biorender.com.

We explore the role of dynamics in multivalent recognition at three levels: i) the equilibrium between the binding of ligand and receptor, ii) the one-dimensional supramolecular dynamics of the ligand-carrying supramolecular polymers, and iii) the two-dimensional dynamics of the receptor-carrying SLB, effectively mimicking cell-membrane bilayer (Fig. 1 and *SI Appendix*, Figs. S1–S6). We have investigated the dynamics of receptor–ligand pairs using different pairs with their binding affinity, including streptavidin–biotin, DNA hybridization, and sialic acid (GD3)-benzoxaborole (Ba), which are discussed later (Fig. 1 and *SI Appendix*, Figs. S2–S5). We realize that the dynamic behavior of components and interactions are time dependent and not easy to quantify. For the dynamic supramolecular polymers, we have exchange rates that range from several minutes to less than 1 h, while for the less-dynamic systems, we assume positional stability within roughly a day (56). We selected the widely studied biotin–streptavidin due to their well-explored high selectivity and affinity, as first receptor–ligand pair. The biotin ligand is covalently bound to one of the monomers of the one-dimensional (1D) supramolecular polymers based on the benzene-1,3,5-tricarboxamide (BTA) core with attached amphiphilic tails (57–59) as a dynamic representative (Biotin-functionalized BTA = BTA-Bio) (Fig. 1). These polymers can also easily be labeled with dyes for microscopic analyses (BTA-Cy3 and BTA-Cy5). The well-studied fast dynamics of these polymers exhibit monomer exchange between fibers occurring within minutes to an hour, while intrafiber monomer exchange is hypothesized to be even faster than few seconds (43, 45). It is important to note that BTA fibers usually exist as double helices. However, to make the concept general and simplify the models, the mathematical and computational models are based on single helices instead. In contrast, for our comparatively less-dynamic representative of 1D supramolecular polymers, we opted for the ureidopyrimidinone-based polymer (UPy-Gly, with biotin-functionalized one as UPy-Bio, *SI Appendix*, Fig. S1). This polymer exhibits relatively slow dynamics, with monomer exchange between fibers occurring negligibly over a span of up to 20 h (56). In comparison lateral diffusion and reorganization of receptors in biological cell membrane typically occur on the time scale of milisecond to second (60, 61).

For dynamic and less-dynamic representatives within the SLB, we selected streptavidin-modified 1,2-dioleoyl-sn-glycero-3-phosphoethanolamine (DOPE) and 1,2-dipalmitoyl-sn-glycero-3-phosphoethanolamine (DPPE) lipids, respectively. Our selection criteria were primarily grounded in their respective diffusion coefficients, which are 1 to 10 μm^2^/s for 1,2-dioleoyl-sn-glycero-3-phosphocholine (DOPC) and 0.006 to 0.1 μm^2^/s for 1,2-dipalmitoyl-sn-glycero-3-phosphocholine (DPPC) (62, 63). This suggests movement of receptors over significant distances within seconds, supporting the notion of dynamics on a submicrometer scale. Functionalized lipids, with postfunctionalization when needed, were used as receptors (Fig. 1 and *SI Appendix*, Figs. S6–S9). It is crucial to emphasize that the selection of these components was based on their relevance to biomaterial studies. Supramolecular polymers based on BTA and UPy units are extensively studied as building blocks for biomaterials, while DOPC and DPPC are natural lipids found in cell membranes.

The distinct dynamics of supramolecular polymers and SLBs play a crucial role in multivalent interactions. Supramolecular polymers exhibit dynamic monomer exchange, occurring over minutes to hours in 1D structures, enabling structural rearrangement and topology changes. Intrafiber monomer exchange is hypothesized to be even faster, potentially within seconds. While this facilitates ligand reorganization, strong receptor binding could also extract ligand monomers, altering ligand density. In contrast, SLBs rely on two-dimensional (2D) lateral diffusion, where lipids and embedded receptors move within the membrane on a submicrometer scale within seconds. Lipid dissociation from the bilayer is highly unfavorable, though receptors bound to lipid head groups may still dissociate. This asymmetry between polymer exchange and membrane diffusion has functional implications. Polymers offer adaptability but risk monomer loss, whereas SLBs provide stability but may limit receptor rearrangement. These differences influence multivalent binding efficiency—if polymer rearrangement is too slow relative to receptor movement, kinetic trapping may occur. Optimizing multivalent interactions requires not only matching the timescales of polymer and membrane dynamics but also ensuring compatibility between their distinct mechanisms: monomer exchange versus 2D diffusion.

### Clustering of Ligand and Receptor due to Dynamics.

**Fig. 2.**
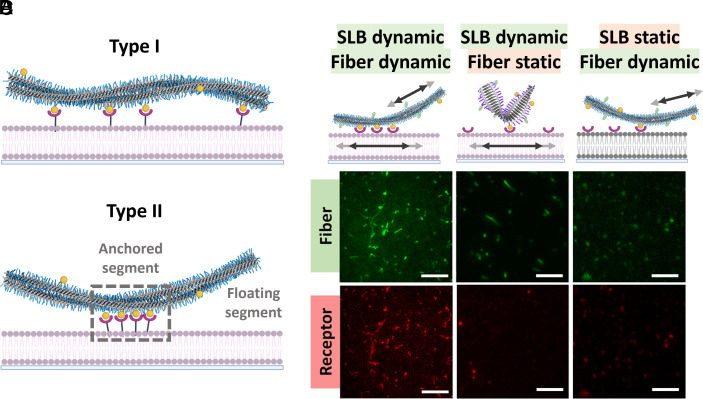
Role of dynamics on anchoring supramolecular polymers and recruitment process. (*A*) Schematic representation of two types of mode of anchoring in the presence of dynamic ligands on supramolecular polymer and dynamic receptors on SLB. (*B*–*D*) Cartoons and two channel TIRF image for fibers and receptor. (*B*) Dynamic BTA fibers and dynamic SLB; (*C*) Less dynamic UPy fibers and dynamic SLB, and (*D*). Dynamic BTA fibers and nondynamic immobile DPPC-based SLB. c_T_,_BTA_ = 2.5 µM, in *B* and *D*. [BTA-Cy3] = 5%, [BTA-Bio] = 1%, *C*. c_T_,_UPy_ = 10 µM, [UPy-Cy3] = 5%, [UPy-Bio] = 1%, *A* and *B*. 0.1% DOPE-Bio in DOPC, *D*. 0.1% DPPE-Bio in DPPC. (Scale bar, 5 µm.) Schematics created with https://Biorender.com.

The difference in dynamics for the clustering and mechanism of anchoring (Fig. 2*A*) was studied using three combinations: BTA + DOPC (dynamic fiber + dynamic bilayer), UPy + DOPC (less-dynamic fiber + dynamic bilayer), and BTA + DPPC (dynamic fiber + less-dynamic bilayer). In these experiments, 5% of monomer-functionalized with biotin as the ligand (BTA-Bio and UPy-Bio) and 5% with fluorescent dye cyanine-3 for the green channel (BTA-Cy3 and UPy-Cy3) were incorporated in the supramolecular polymers. In addition, 0.1% of SLB was functionalized with fluorescently labeled streptavidin as the receptor for the red channel (*Materials and Methods*) to be able to track ligand and receptor dynamics.

We employed two-channel imaging using total internal reflection fluorescence (TIRF) microscopy to visualize the interaction between supramolecular polymer and bilayer. In the case when both BTA fibers and DOPC are dynamic, we observed substantial attachment of the BTA fibers to the DOPC (Fig. 2*B* and *SI Appendix*, Fig. S10). Remarkably, streptavidin receptors that initially displayed a uniform distribution in the absence of fibers spontaneously rearranged themselves to align with the fiber-appended ligands (*SI Appendix*, Figs. S9 and S10). Notably, even when we employed nonfluorescent fibers (without BTA-Cy3) to eliminate potential crosstalk between the two channels, we still observed receptor rearrangement within fiber-like structures (*SI Appendix*, Fig. S11). These results suggest that dynamic supramolecular polymers have the capacity to anchor themselves to the dynamic DOPC while concurrently recruiting receptors. In contrast, the less dynamic UPy fibers failed to bind to the dynamic DOPC in the same experimental setup, and no receptor recruitment was evident except a few attachment points as red spots (Fig. 2*C* and *SI Appendix*, Figs. S12 and S13). Even after a prolonged 30-min period, we observed only limited anchoring of fibers and minimal recruitment of receptors, as depicted by the movement of fibers (*SI Appendix*, Fig. S14). Similarly, the interaction of dynamic BTA fibers with nondynamic DPPC did not show any significant attachment of fibers to the DPPC, nor did we observe any receptor recruitment (Fig. 2*D* and *SI Appendix*, Fig. S15). The data show that to arrive at a fast—within a few minutes—attachment of fibers to the SLB, both BTA and DOPC must be dynamic. This clearly indicates that the outcome of attachment is influenced by the physicochemical characteristics of the constituent fiber and membrane components. The dynamic nature of both components is a prerequisite to arrive at the mobility of the functional units within the polymers in 1D as well as the bilayers in 2D.

Given the high affinity between streptavidin and biotin, as well as the inherent multivalency of the supramolecular polymers and SLBs which is independent of dynamics, the question arises why do such disparities exist among the three cases? To answer this, we need to unravel the mode by which supramolecular polymers anchor to SLB and how dynamic processes either facilitate or modify this anchoring mechanism (Fig. 2*A*). In the context of monovalent single-point interactions (involving one ligand and one receptor), the anticipation is that interactions should occur identically across all cases. When these two partners encounter each other randomly, they are likely to interact and remain bound due to high binding affinity of streptavidin–biotin. As a result, over time, even when one partner is less dynamic, only a few interaction sites are expected to form. This leads to a limited number of anchoring points, which corresponds to a type-I mode, where no rearrangement happens for the interactions to occur (Fig. 2*A*). Moreover, a potential defect mechanism is possible that the less dynamic partner detaches some of the streptavidin or biotin from the dynamic partner due to very strong receptor–ligand binding. However, it is important to note that in scenarios where a receptor–ligand pair exhibits weaker interaction, as elaborated upon later in this study, the attachment with nondynamic partner is not expected to be favored even with prolonged incubation due to weak individual binding. Moreover, despite the presence of multiple ligands or receptors in the polymer or SLB, effective multivalency heavily depends on the arrangement and spacing of both partners. Thus, the lack of rearrangement does not lead to the required perfect fit in spacing of both partners. Optimal spacing is critical for efficient multivalent interactions; overly close packing can lead to steric hindrance and hinder simultaneous binding, while excessive spacing can weaken cooperative effects (Fig. 2*A*) (40, 64). For example, when only 1 in 20 monomers are functionalized with a ligand in the supramolecular polymer and 1 in 1,000 lipids are functionalized with a receptor in the SLB, the considerable distance between individual interactions results in low to nonexistent cooperativity and lack of any multivalency, as will be shown by detailed stochastic simulations below. Therefore, in the case of static receptors and ligands, it is highly unlikely for all receptors to interact with all ligands, primarily due to the statistical distribution of each within their respective host matrices. The mobility ensuing the dynamic nature and mobility of both the fibers and the SLB facilitates the rapid rearrangement of ligands or receptors in their respective hosts. Then, the cooperativity in multivalency is enhanced due to the reduced distance between individual interactions, i.e., the clustering of receptors which is illustrated in our proposed type-II mode of anchoring (Fig. 2*A*). This phenomenon of reciprocity mirrors how viruses interact with cells through multivalency and clustering with simultaneous cooperation (53).

### Multivalent Binding in Clusters.

**Fig. 3.**
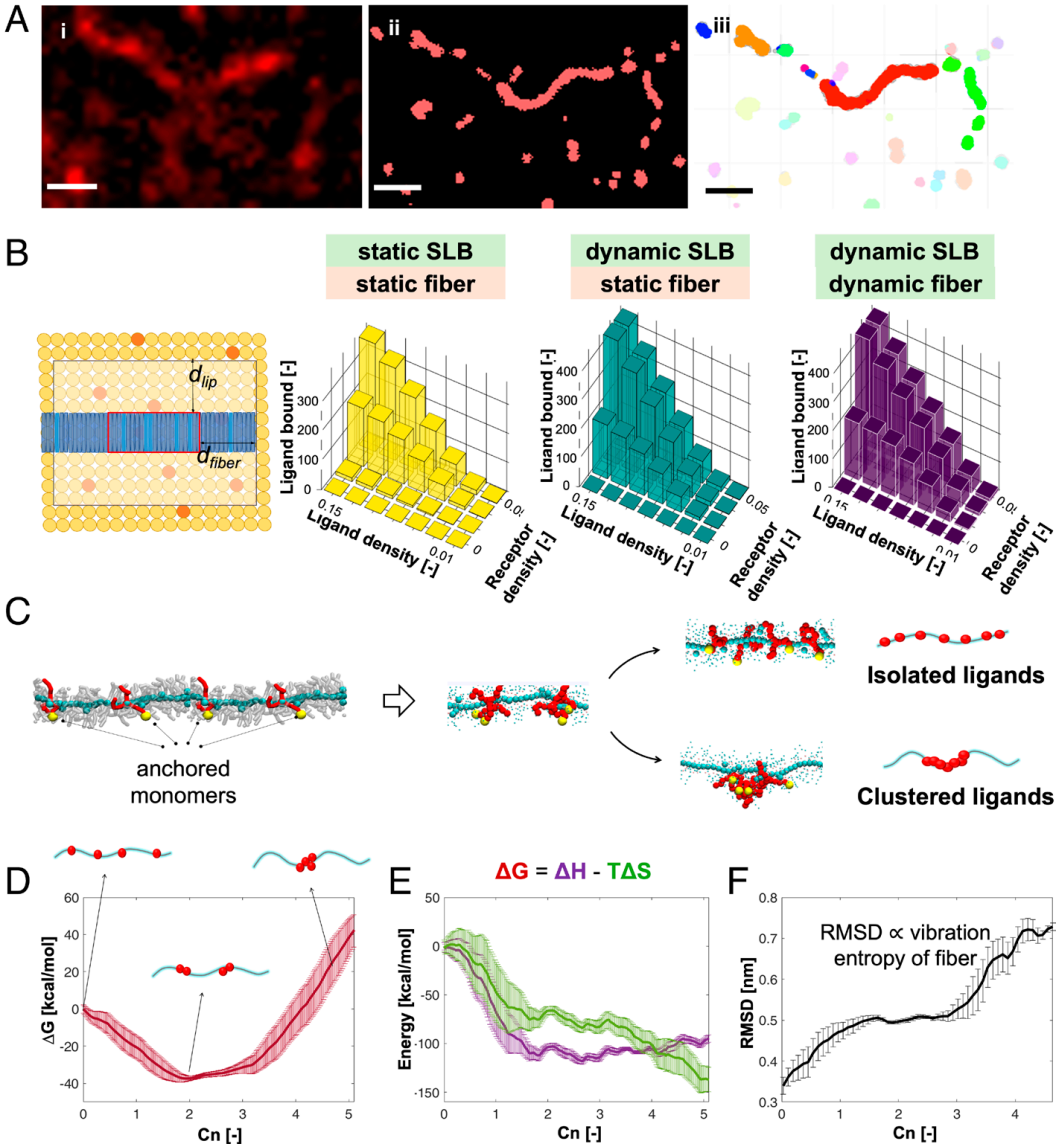
Clustering mechanism. (*A*) Cluster analysis of recruited receptors i) TIRF, ii) STORM and iii) Clusters. c_T_,_BTA_ = 2.5 µM, in [BTA-Cy3] = 5%, [BTA-bio] = 1%, 0.1% DOPE-Bio in DOPC, 0.1% SAvCy5. (Scale bar, 1 µm.) (*B*) This cartoon illustrates the clustering constraint for stochastic simulations. On the membrane (yellow), some monomers contain receptors (orange), while on the fiber (dark blue), certain monomers contain ligands (light blue). For binding to occur, the area in the red box, which has a width of d_fiber_, should contain at least five ligands and the highlighted area of the membrane (bounded at a distance d_lip_ from the red box) must contain at least n_cluster_ receptors. The following conditions are tested (left to right) when both nondiffusing lipids and ligands (D_lip_ = 0, D_lig_ = 0) demonstrated low probability of binding, diffusing lipids and nondiffusing ligands (D_lip_ > 0, D_lig_ = 0) showed medium binding and cluster formation, and both diffusing lipids and ligands (D_lip_ > 0, D_lig_ > 0) exhibit strong cluster formation tendencies. Ligand and receptor density versus the mean number of bound ligands at t = 500 based on 1,000 simulations (*C*) Coarse-grained model of a BTA-fiber containing four ligand functionalized monomers bound to a surface (red colored, yellow dots indicate the anchoring points. (*D*) Clustering free energy profile as a function of the ligands’ coordination number (Cn) computed from the MetaD simulation. High Cn indicates fiber’s configurations where larger clusters are formed, while Cn=0 identifies a state where all ligands are separated in the bound fiber. (*E*) Decomposition of the clustering free energy profile into enthalpic (purple) and entropic terms (green). (*F*) Rms displacement measurement showing increase in degree of freedom, i.e., the conformational variability of BTA fiber configurations with increasing Cn (relative only to the conformational/vibrational entropy of the fiber in the various bound configurations).

A cluster formation of ligands and receptors at the anchoring site, must result in alternating clustered and floating segments, as shown in type-II mode of anchoring (Fig. 2*A*). To further investigate the role of valency in the system, we systematically changed the concentration of both partners in streptavidin–biotin pair. A nonlinear dependency on receptor and ligand concentrations was observed (*SI Appendix*, Figs. S16 and S17), indicating multivalent interactions. At low concentrations of both receptor and ligands (<0.05% of SAv and <0.1% BTA-Bio), we observed no fiber attachment. In contrast, at high concentrations of both receptor and ligands (>0.1% of SAv and >1% BTA-Bio), we observed complete fiber attachment. Notably, within an intermediate (0.05% of SAv and 1% BTA-Bio or 0.1% SAv and 0.1% BTA-Bio) concentration range, fibers appeared to be only partially attached, and receptors were clustered at the anchoring site, supporting type-II mode of anchoring (Fig. 2*A*). To visualize this at the single molecule level, we conducted stochastic optical reconstruction microscopy (STORM) superresolution imaging. STORM images of receptors after the attachment of supramolecular polymers BTA to DOPC-based SLB revealed a noncontinuous distribution of fluorescent receptors along the polymer backbone, consistent with the anticipated type-II mode of attachment by cluster formation (Fig. 3*A* and *SI Appendix*, Fig. S18). Cluster analysis using MATLAB based on the nearest-neighbor algorithm and density-based spatial clustering of applications with noise (DBSCAN) further confirmed the presence of clusters of different sizes along the polymer backbone (65). It is worth noting that large clusters could be either composed of numerous small clusters that could not be individually resolved due to the resolution limit of ca. 50 nm or may result from the aggregation of small clusters through the dynamic ligand enrichment process (discussed below, *SI Appendix*, Fig. S19).

### Stochastic Simulations.

To support the observed superselective binding in our system, where the concentration and dynamics of both partners are important as well as the interaction energy between them, we performed stochastic simulations. We propose a stochastic model that approximates binding phenomena via constraints on adjacent receptors and ligands (Fig. 3*B* and *SI Appendix*, Figs. S20–S27). For simplicity, this model considers a single fiber with n_fiber_ = 3,001 monomers of ca. 1,000 nm, and each monomer of 3 nm diameter and ×1/3 nm height (Fig. 3*B*). The number of ligand-functionalized monomers, N_lig_, follows a Poisson distribution, with ligands randomly placed and diffusing along the fiber according to a discretized Brownian motion governed by the diffusion coefficient (D_lig_). The fiber interacts with receptors on a 1 μm × 1 μm lipid membrane comprising n_membrane_ = 2,000 × 2,000 monomers of 1 nm diameter. The receptor quantity N_lip_ also follows a Poisson distribution, with receptors undergoing two-dimensional Brownian motion characterized by the lipid diffusion coefficient (D_lip_). Binding occurs under a clustering restriction, where detailed molecular interactions are replaced by a stochastic process. Bound ligands and receptors form static clusters over time. We define cluster formation when n_cluster_ = 5 ligands and receptors are present in the region of fiber length d_fiber_ = 20 monomers, and lipid membrane area d_lip_ = 40 lipids, as illustrated in Fig. 3*B* and *SI Appendix*, Fig. S26. If the cluster restriction applies, ligand–receptor pairs are then optimally matched by minimizing distances. We examined three dynamic scenarios for ligands and lipids: i) both nondiffusing (D_lip_ = 0, D_lig_ = 0), ii) diffusing lipids and nondiffusing ligands (D_lip_ > 0, D_lig_ = 0) with irreversible binding, and iii) both diffusing (D_lip_ > 0, D_lig_ > 0). In the third scenario, we tracked ligand-functionalized monomers that met the clustering criteria without fixing ligands or lipids.

The average number of ligands that met the clustering criteria in this scenario was equivalent to that in a fixed state, no diffusion (D_lip_ = 0, D_lig_ = 0). In these simulations, we considered three cooperative receptor–ligand binding events that occurred simultaneously within a confined region as a cluster (Fig. 3*B*). Under conditions similar to our experimental settings where no dynamics were allowed, the probability of binding was low (Fig. 3 *B*, *i*). However, when receptors were introduced as dynamic entities, cluster formation increased (Fig. 3 *B*, *ii*). Finally, when both receptors and ligands were dynamic, even at low concentrations of both, our stochastic simulations demonstrated a higher cluster formation tendency (Fig. 3 *B*, *iii*). Moreover, a nonlinear dependence between the concentration of ligands, receptors, and their binding affinity was observed (*SI Appendix*, Fig. S22). Dynamics thus plays a major role in supporting the type-II mode of the anchoring mechanism.

Although the stochastic model can represent a wide range of receptor–ligand pairs with different orders of K_a_ (*SI Appendix*, Figs. S23–S27), we used an irreversible binding model to isolate the effects of diffusion and clustering on initial binding events while minimizing computational expense. Introducing reversible binding, which is more relevant for lower-affinity ligand–receptor systems, would establish a dynamic equilibrium at the interface between the supramolecular polymer and the SLB. This would facilitate greater exploration of binding configurations and enhance multivalent interactions over time, as observed in the “zipping effect” (see below).

### Coarse-Grained (CG) Molecular Modeling of Ligand Clustering.

To gain further insights into the underlying mechanisms of the type-II mode of anchoring, we developed a minimalistic CG molecular model allowing to explore the most energetically favored ligands’ clustering configuration in the bound fibers (Fig. 3 *C*–*F* and *SI Appendix*, Fig. S29) (66, 67). For simplicity, this model features a single BTA fiber containing a fixed number (N) of ligand-functionalized monomers, which are irreversibly bound to N receptors (R) on a SLB surface (Fig. 3*C*: N = 4 bound ligands and fiber length (l) = 40 free BTA monomers, respectively, in N:l ratio of 1:10). This model builds on the fact that the ligand–receptor interactions are very strong compared to the interaction of each monomer with its neighbors in the stacks, so that the dynamics of ligand–receptor unbinding can be assumed as negligible as compared to the monomers’ reshuffling and the overall internal supramolecular dynamics of the fibers and of the SLB surface (43). Biased Metadynamics (MetaD) simulations (43, 67, 68) were then performed during which the monomers that are (irreversibly) bound to the surface can slide and move along the fiber (46), exploring various possible clustering arrangements to optimize the interaction and the internal energy (see computational details in the *SI Appendix*). This allowed us to investigate the most thermodynamically favorable ligand configurations within this model during the fiber multivalent binding (Fig. 3*C*: anchored monomers). Given the relatively small size of such models (compared to the real experimental system), they do not aim at providing a comprehensive ensemble picture of clustering or of the clusters size distribution in the experimental scenarios, but rather to assess in general the propensity of the system to establish L-R local interactions that are individual/separated or clustered in space, and to assess the molecular mechanisms driving the clustering on a local basis, and the associated energetic, enthalpic, and entropic penalties/gains.

The results obtained from the MetaD simulations indicate how the fiber internally reorganizes to optimize the multivalent interaction (*SI Appendix*). From the MetaD simulations, we were able to estimate the ligands’ clustering free energy profile of Fig. 3*D*. This profile indicates that generating N separate (monovalent) binding sites (i.e., no clustering and ligands’ coordination number: Cn = 0) is thermodynamically unfavorable. The same is true for having all ligands gathered in one individual large cluster (Cn > 4), which is found highly unfavorable. The minimum of the free energy is observed for configurations where the fiber reorganizes to form an intermediate number of clusters of bound monomers (0 < Cn < 4: intermediate clustering). We decomposed the free energy profile of Fig. 3*D* into its enthalpic and entropic contributions (Fig. 3*E* and *SI Appendix* for more technical details) (43, 69–71). The shape of the green curve indicates that the global entropy penalty of the system in extreme clustering state (Cn-max) is related to the very “ordered” and entropically unfavored nature of this state (Fig. 3*E*). Interestingly, the Cn > 4 configuration state is characterized by a total low entropy even though the fiber as a whole has higher freedom of movement when all bound monomers are gathered together into a single binding cluster: the Rms displacement plot in Fig. 3*F*—indicative of the conformational degrees of freedom of the fiber in the various clustering states—show how the fiber is globally more free to move when all ligands are clustered together into a single multivalent binding spot and the fiber tips are free to oscillate. Altogether, this indicates how the conformational entropy of the fibers alone is not sufficient to estimate the behavior of such reconfigurable systems upon multivalent binding, while the entropic penalty (unlikelihood) of observing specific ordered clustering states (e.g., where all ligands are clustered together, or are all separated or equidistant along the fibers) becomes a dominant factor. Consistent results proving that intermediate clustering levels are the most favored ones (i.e., with multiple clusters’ containing multiple L-R interactions) have been obtained even from a larger model with increased fiber length and a higher number of anchored ligands (*SI Appendix*, Fig. S28). While it is important to note that the number of ligands and receptors involved in one single cluster in the real system may depend on the system (e.g., on the concentration, the L:R ratio, the distribution and variability of the real fibers’ lengths, etc.) and the specific conditions, even considering the simplified nature of these models, these findings align with and complement the experimental evidence, and the results obtained from stochastic simulations in proving that the formation of clusters is highly likely during multivalent interactions between dynamical entities such as the ones studied herein.

The superselective binding and receptor clustering observed in our system distinguish it from purely avidity-driven interactions. While avidity leads to gradual binding increases, the sharp transition seen in our experiments—supported by STORM imaging and stochastic simulations—suggests a cooperative mechanism in which each binding event amplifies the next, leading to nonlinear affinity responses. Moreover, stochastic and molecular dynamics simulations confirm that clustering and strong binding occur only when both ligands and receptors are dynamic. Furthermore, our simulations reveal that intermediate cluster formation is entropically favored, reinforcing the hypothesis that cooperativity emerges from dynamics-driven enrichment rather than just site proximity effects.

### Mechanistic Insights on Clustering—DNA Association Constant.

**Fig. 4.**
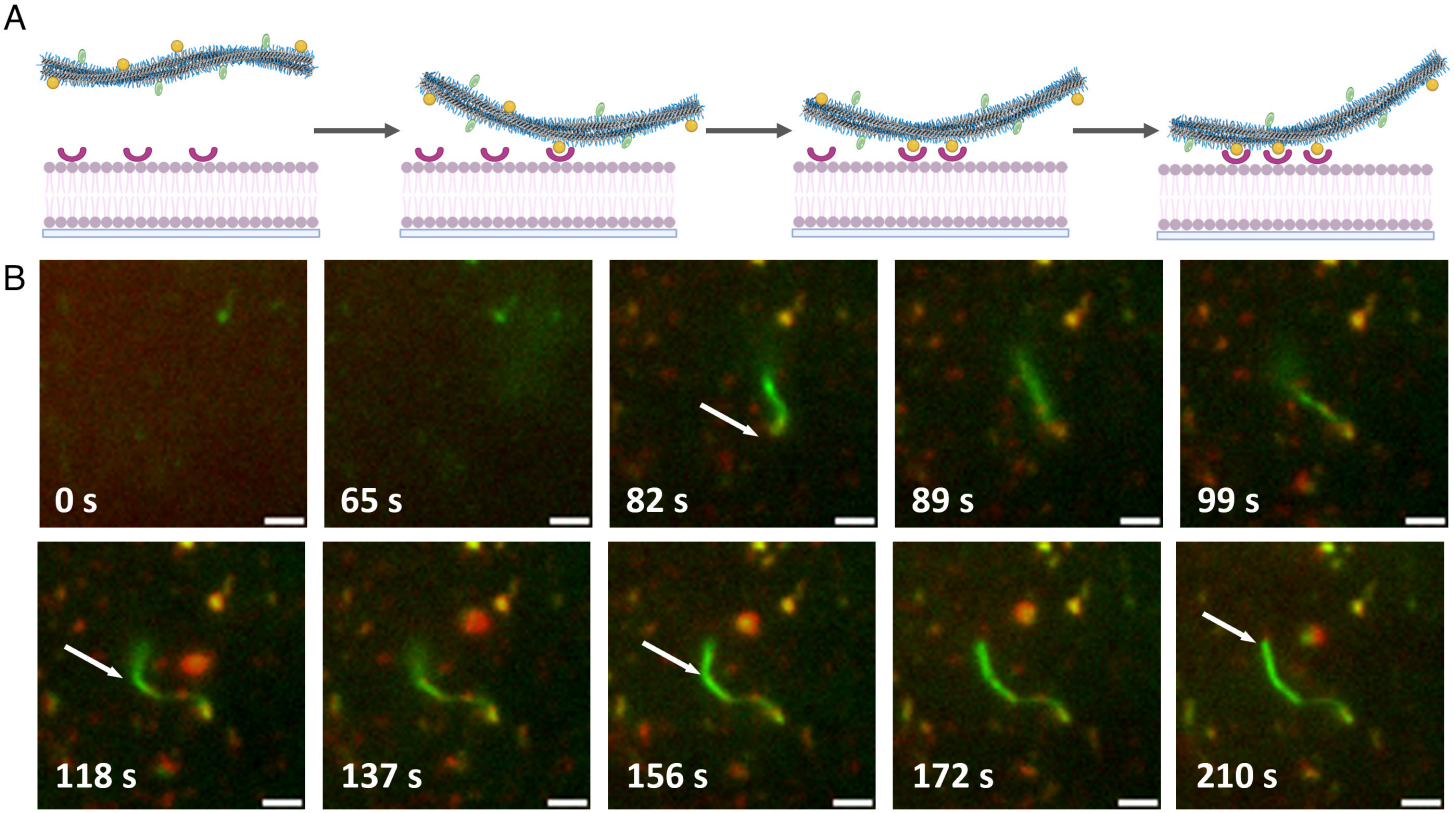
Proposed mechanism of adhesion of fiber to SLB via highly coordinated sequential binding of multiple ligand–receptor pairs. (zipping effect) (*A*) Schematic, (*B*) Time-lapse TIRFM imaging showing zipping effect, arrows represent attachment points. [Receptor] = 0.1 mol%, [BTA-DNA] = 1%, [BTA-Cy3] = 5%, DNA_3_Biotin = 1 eq., c_T_,_BTA_ = 2.5 μM, (Scale bar, 2 μm.) Red channel = receptor, Green = BTA-Cy3. Schematic created with https://Biorender.com. The arrows represent the new binding site between supramolecular polymers and SLB.

In general, investigations assume that the effect of multivalent clustering depends on the association constant of the receptor–ligand pair (72). To investigate the generality and dependence of the clustering mechanism on the association constant of the receptor–ligand pair, we used DNA base pairing as the receptor–ligand pair. The association constant of the DNA double helix is proportional to the length of the DNA binding motif. For this study, the receptor used was DNAn-Bio (n = 3, 5, 8, and 10), which interacts via SAv-Bio interaction on a DOPE-Bio based SLB (Fig. 1 and *SI Appendix*, Fig. S29). We examined the binding of BTA-DNA with the complementary sequence to the SLB and observed that decreasing the length of DNAn-Bio reduced both the extent and rate of fiber binding to the SLB (*SI Appendix*, Fig. S30). This observation directly correlates with the association constant of double-stranded DNA formation (*SI Appendix*, Fig. S31). For further insights, we recorded the time-dependent adhesion of the fiber to the SLB. A highly coordinated process using DNA_3_Bio was observed with successive binding of multiple ligand–receptor pairs (Fig. 4, *SI Appendix*, Figs. S32 and S33, and Movie S1). The process starts with an initial binding occurrence between a receptor and a ligand as the fiber approaches the SLB. Subsequently, other regions of the fiber approach the SLB, where the dynamic nature leads to ligand rearrangement to obtain a high local concentration of ligands and the concomitant increase in the probability of receptor recruitment. Thus, the binding process creates a zipping effect as the fibers pull themselves along the SLB, causing more ligand–receptor pairs to bind that eventually lead to the attachment of the fibers to the SLB. This follows type-II mode of anchoring (Fig. 2*A*). These findings highlight the importance of synchronized dynamics between partners to act cooperatively. The rate-limiting step depends on the specific system: For weaker receptor–ligand pairs, binding affinity and association rates may slow down zipping, whereas for stronger receptor–ligand interactions, the diffusion of receptors and ligands may become the bottleneck.

Furthermore, when either ligand dynamics (UPy fibers) or receptor dynamics (DPPC membranes) are restricted, strong binding and clustering do not occur, even in a high-affinity streptavidin–biotin system (Fig. 2). This further supports the idea that synchronized dynamics between the fiber and SLB are critical for cooperative multivalent interactions. If the internal dynamics of the polymer, such as monomer exchange, are significantly slower than the movement and potential clustering of receptors in the SLB, the system might not be able to achieve or maintain optimal ligand–receptor arrangements. This could lead to suboptimal binding or a form of “kinetic trapping” where energetically favorable configurations are not reached within the relevant timeframe of interaction. Furthermore, we consider the role of dynamics in promoting the enrichment of ligands within the polymer backbone of anchored fibers during and after zipping. This enrichment must occur for additional receptor–ligand interactions to achieve an optimal spatial arrangement for individual interactions and between the multivalent clusters. Therefore, the system must evolve toward thermodynamic equilibrium. This enrichment hypothesis can be substantiated by the observed retention of dynamic behavior of anchored BTA-based supramolecular polymer as evidenced by the rapid exchange of monomers (labeled with fluorescent markers) between anchored and free BTA fibers (*SI Appendix*, Fig. S34). Unlike conventional multivalent systems, where avidity-driven interactions dominate, our results demonstrate that cooperative binding in dynamic supramolecular polymers and SLBs follows a zipping mechanism, reinforced by ligand–receptor rearrangement. This process is not solely due to increased local effective concentration but emerges from the coordinated mobility of both binding partners, which we define as “reciprocity.” Reciprocity in our system refers to the dynamic interplay between ligand-functionalized supramolecular polymers and receptor-functionalized SLBs, enabling binding partners to rearrange and optimize multivalent interactions. Without reciprocity, interactions remain isolated, lacking the reinforcement needed for cluster formation. While reciprocity facilitates cooperativity in our system, cooperativity can also occur in other contexts such as the allosteric effect, without requiring reciprocal dynamics.

### The Sialic Acid–Benzoxaborole System.

**Fig. 5.**
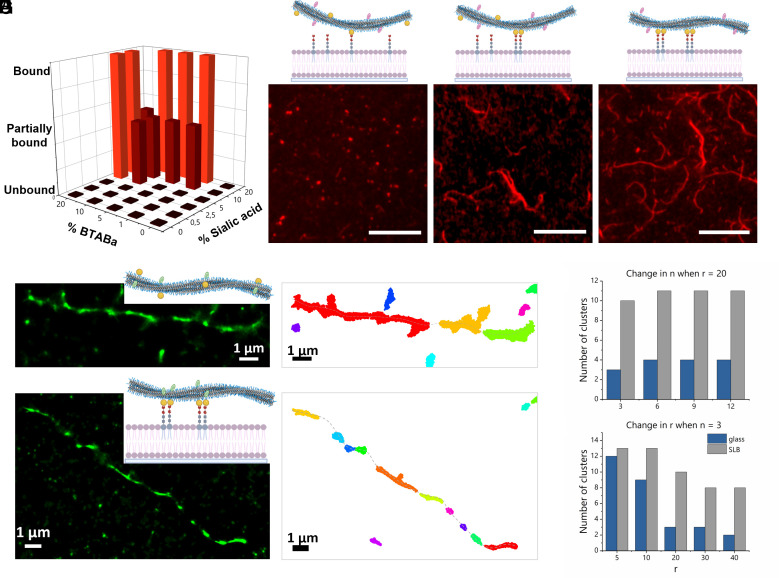
Anchoring of BTA-Ba supramolecular polymers to GD3-based SLB. (*A*) Effect of change in % ligand (BTA-Ba) and % receptor (Sialic acid) on anchoring of supramolecular polymers to SLB. Schematic representation and TIRF image of *B*. Unbound state [Sialic acid] = 2.5%, (*C*) Partially bound state [Sialic acid] = 10 mol % and (*D*) Completely bound state [Sialic acid] = 20 mol %. [BTA-Ba] = 1%, [BTACy5] = 5%, c_T_,_BTA_ = 2.5 µM, (Scale bar, 10 µm.) (*E*) STORM image and corresponding cluster analysis of BTABaCy3 fibers on *E*. Glass and *F*. GD3-based SLB. No. of cluster dependence on change in *G*. nearest neighbor (n) and (*H*). radius (r) in clustering algorithm on glass and on SLB. Schematics created with https://Biorender.com.

To test the generality of our theory on the role of concurrent dynamics in facilitating the type-II mode of anchoring, we used another ligand–receptor pair used in our group before in studying the interaction of the supramolecular polymer with hRBCs (49). Specifically, we used benzoxaborole as a ligand in the BTA supramolecular polymer (referred to as BTA-Ba) and sialic acid (GD3) as the receptor on a DOPC-based SLB (*SI Appendix*, Figs. S35 and S36). This pair features a relatively low equilibrium constant for this chemical reaction (40 M^−1^) with a high reversibility. The TIRF microscopy images revealed a dependence between fiber attachment and receptor/ligand concentration, indicative of the presence of a multivalent effect (Fig. 5 *A*–*D* and *SI Appendix*, Figs. S37 and S38). Notably, intermediate concentration ranges showed partial fiber attachment to the SLB, indicating the existence of clusters and a type-II mode of anchoring (Fig. 2*A*). Interestingly, a 1% BTA-Ba fiber with 10% GD3 in DOPC in the SLB mimicked the partial attachment and partial displacement of fibers (Fig. 5*C* and *SI Appendix*, Fig. S38), similar to what was observed with BTA fibers and hRBCs (49). Interestingly, we observed that at high concentrations of both ligand and receptor, fiber–surface interactions decreased, suggesting an “oversaturation” effect. This trend, also seen in our earlier studies, points to reduced fiber–surface interaction with increased BTA-Ba incorporation (49). We hypothesize that excessive functionalization reduces fiber stability—possibly due to the presence of unbound BTA-Ba monomers after clustering—which may further destabilize portions of the fiber structure.

To investigate ligand clustering at the binding site, we designed asymmetric BTAs with both the ligand benzoxaborole and Cy3 (a fluorescent marker) on the same monomer, denoted as BTA-BaCy3 (*SI Appendix*, Fig. S4). STORM images indicated that fibers in the absence of SLB had a relatively uniform distribution of Cy3 fluorescence emission, indicating the dispersed nature of the ligands (Fig. 5*E*). However, when fibers were anchored on the SLB functionalized with GD3, ligands clustered at a few random sites based on the concentrated emission of the BTA-BaCy3, supporting the concept of a type-II mode of anchoring and dynamic receptor recruitment (Figs. 5*F* and 2*A*). To further substantiate these findings, a cluster analysis with DBSCAN was performed. Variations in initial parameters, such as the number of neighbors (n) in a particular radius of neighbors (r), revealed that the relative number of clusters for the fibers on SLB remained higher than on glass (Fig. 5 *G* and *H* and *SI Appendix*, Figs. S39 and S40). This observation shows that fibers with alternating bound and floating segments on the SLB engage in multivalent clustering, akin to what is observed in biological systems. The formation of these clusters thus involves the simultaneous participation of receptors and ligands in a dynamic manner. Our experimental and theoretical models are thus good representatives of the interaction of supramolecular materials with cells and highlight the role of dynamics to drive the molecular recognition process through multivalent clustering at varying binding affinity between receptor–ligand pair.

To check whether receptors within clusters are static or dynamic, we used single particle tracking with photoactivated localization microscopy (73). In the dynamic receptor system, receptors exhibited high lateral mobility, yielding a diffusion coefficient of 0.8 × 10^−2^ µm^2^/s (*SI Appendix*, Figs. S41 and S42). In contrast, the presence of dynamic BTA-based supramolecular polymers in the dynamic receptor system resulted in two receptor populations: one mobile and another nearly stationary with a diffusion coefficient of 2.8 × 10^−2^ µm^2^/s. This stationary population closely matched the diffusion coefficient observed in immobile DPPC-based bilayers (2.7 × 10^−2^ µm^2^/s). Thus, the receptors in clusters appear to be static based on these observations.

### Summary.

This study systematically investigated the role: i) monomer exchange dynamics within the supramolecular polymers, ii) lipid diffusion within the SLB, and iii) the association/dissociation kinetics of the receptor–ligand pairs. It demonstrates how dynamic reciprocity between ligands on supramolecular polymers and receptors on model cell membranes governs molecular recognition and multivalent binding through receptor clustering. Efficient multivalent binding occurs through type-II mode of anchoring only when ligand mobility (monomer exchange in supramolecular polymers) and receptor mobility (lipid diffusion in SLB) are synchronized with receptor–ligand association/dissociation kinetics. Systems with mismatched dynamics, such as less-dynamic UPy fibers or DPPE membranes, fail to achieve significant attachment and clustering, even with high-affinity interactions like streptavidin–biotin, due to kinetic barriers that prevent optimal binding. Computational modeling suggests that the clustering process is entropy-driven, with receptor–ligand pairs minimizing distances dynamically to enhance cooperative binding. This process follows a zipping effect, where binding occurs sequentially through coordinated ligand–receptor interactions, reinforced by partner mobility rather than just increased local concentration and avidity.

These insights establish key design principles for biomaterials: incorporating dynamic components that match biological receptor motion, leveraging the zipping mechanism for enhanced adhesion, and optimizing ligand density for thermodynamically favorable clustering. By aligning dynamic properties with biological timescales, this work advances the development of supramolecular biomaterials for biomedical applications, including cell-membrane interactions, biological multivalency, superselectivity, and receptor clustering in cell signaling (74, 75).

## Materials and Methods

### Synthesis and Experiments.

All reagents and chemicals were obtained from commercial sources at the highest purity available and used without further purification unless stated otherwise. All solvents were of AR quality. DOPC, DOPE-N-(cap biotinyl), sodium salt (DOPEBio), DPPC, were obtained from Avanti Polar Lipids. Texas Red™ 1,2-Dihexadecanoyl-sn-Glycero-3-Phosphoethanolamine, Triethylammonium Salt (TRDHPE), and Streptavidin conjugated with Alexa Fluor 488 were obtained from ThermoFisher. Streptavidin from *Streptomyces avidinii* was obtained from Merck. Streptavidin conjugated with Abberior CAGE 635 was purchased from Abberior. DNA oligomers were purchased from IDTDNA. Μ-Slide 8 Well plate for TIRF microscopy was obtained from Ibidi. Water was purified on an EMD Millipore Milli-Q Integral Water Purification System. SiO_2_-coated QCM sensors (QSX 303) were purchased from Biolin Scientific. Reactions were followed by thin-layer chromatography (precoated 0.25 mm, 60-F254 silica gel plates from Merck). Dry solvents were obtained with an MBRAUN Solvent Purification System (MB-SPS). Automated column chromatography was performed on a Biotage Isolera using Biotage® SNAP-KP SIL cartridges.

The following methods are explained in the *SI Appendix*: NMR, Liquid chromatography–mass spectrometry (LC–MS), Matrix-assisted laser absorption/ionization mass time of flight (MALDI-TOF), High-performance liquid chromatography (HPLC), Reversed Phase-Medium pressure liquid chromatography (RP-MPLC), Total internal reflection fluorescence (TIRF) microscopy, Stochastic Optical Reconstruction Microscopy (STORM) imaging, Quartz Crystal Microbalance with Dissipation monitoring (QCM-D) measurements, Dynamic Light Scattering (DLS) measurements, MATLAB cluster analysis, and SPT-PALM.

## Supplementary Material

Appendix 01 (PDF)

Movie S1. TIRFM video showing zipping effect.

## Data Availability

All study data are included in the article and/or supporting information.

## References

[1] A. S. Mao, D. J. Mooney, Regenerative medicine: Current therapies and future directions. Proc. Natl. Acad. Sci. U.S.A. **112**, 14452–14459 (2015). 26598661 10.1073/pnas.1508520112 PMC4664309

[2] C. M. Rubert Pérez , The powerful functions of peptide-based bioactive matrices for regenerative medicine. Ann. Biomed. Eng. **43**, 501–514 (2015). 25366903 10.1007/s10439-014-1166-6 PMC4380550

[3] O. J. G. M. Goor, S. I. S. Hendrikse, P. Y. W. Dankers, E. W. Meijer, From supramolecular polymers to multi-component biomaterials. Chem. Soc. Rev. **46**, 6621–6637 (2017). 28991958 10.1039/c7cs00564d

[4] S. Patra, S. Chandrabhas, S. Dhiman, S. J. George, Controlled supramolecular polymerization via bioinspired, liquid-liquid phase separation of monomers. J. Am. Chem. Soc. **146**, 12577–12586 (2024). 38683934 10.1021/jacs.4c01377

[5] T. Aida, E. W. Meijer, S. I. Stupp, Functional supramolecular polymers. Science **335**, 813–817 (2012). 22344437 10.1126/science.1205962 PMC3291483

[6] J. Zhou, J. Li, X. Du, B. Xu, Supramolecular biofunctional materials. Biomaterials **129**, 1–27 (2017). 28319779 10.1016/j.biomaterials.2017.03.014 PMC5470592

[7] P. R. A. Chivers, D. K. Smith, Shaping and structuring supramolecular gels. Nat. Rev. Mater. **4**, 463–478 (2019).

[8] T. F. A. de Greef, E. W. Meijer, Supramolecular polymers. Nature **453**, 171–173 (2008). 18464733 10.1038/453171a

[9] R. C. Ollier, Y. Xiang, A. M. Yacovelli, M. J. Webber, Biomimetic strain-stiffening in fully synthetic dynamic-covalent hydrogel networks. Chem. Sci. **14**, 4796–4805 (2023). 37181784 10.1039/d3sc00011g PMC10171040

[10] J. Boekhoven, S. I. Stupp, 25th anniversary article: Supramolecular materials for regenerative medicine. Adv. Mater. **26**, 1642–1659 (2014). 24496667 10.1002/adma.201304606 PMC4015801

[11] M. J. Webber, E. A. Appel, E. W. Meijer, R. Langer, Supramolecular biomaterials. Nat. Mater. **15**, 13–26 (2016). 26681596 10.1038/nmat4474

[12] J. J. Rice , Engineering the regenerative microenvironment with biomaterials. Adv. Healthc. Mater. **2**, 57–71 (2013). 23184739 10.1002/adhm.201200197

[13] J. P. Wojciechowski, M. M. Stevens, A dynamic duo. Science **374**, 825–826 (2021). 34762463 10.1126/science.abm3881

[14] E. Fuentes , Supramolecular stability of benzene-1, 3, 5-tricarboxamide supramolecular polymers in biological media: Beyond the stability-responsiveness trade-off. J. Am. Chem. Soc. **144**, 21196–21205 (2022). 36368016 10.1021/jacs.2c08528 PMC9706554

[15] S. Chagri, D. Y. W. Ng, T. Weil, Designing bioresponsive nanomaterials for intracellular self-assembly. Nat. Rev. Chem. **6**, 320–338 (2022). 37117928 10.1038/s41570-022-00373-x PMC8972907

[16] A. Uvyn , Hapten/myristoyl functionalized poly(propyleneimine) dendrimers as potent cell surface recruiters of antibodies for mediating innate immune killing. Adv. Mater. **35**, 2303909 (2023). 10.1002/adma.202303909 37572294

[17] E. Persch, O. Dumele, F. Diederich, Molecular recognition in chemical and biological systems. Angew. Chem. Int. Ed. Engl. **54**, 3290–3327 (2015). 25630692 10.1002/anie.201408487

[18] J. M. Fox, M. Zhao, M. J. Fink, K. Kang, G. M. Whitesides, The molecular origin of enthalpy/entropy compensation in biomolecular recognition. Ann. Rev. Biophys. **47**, 223–250 (2018). 29505727 10.1146/annurev-biophys-070816-033743

[19] A. Grakoui , The immunological synapse: A molecular machine controlling T cell activation. Science **285**, 221–227 (1999). 10398592 10.1126/science.285.5425.221

[20] W. He, Q. Wang, X. Tian, G. Pan, Recapitulating dynamic ECM ligand presentation at biomaterial interfaces: Molecular strategies and biomedical prospects. Exploration **2**, 20210093 (2022). 37324582 10.1002/EXP.20210093 PMC10191035

[21] V. Ravnik, U. Bren, T. Curk, Designing multivalent copolymers for selective targeting of multicomponent surfaces. Macromolecules **57**, 5991–6002 (2024).

[22] G. V. Dubacheva, T. Curk, D. Frenkel, R. P. Richter, Multivalent recognition at fluid surfaces: The interplay of receptor clustering and superselectivity. J. Am. Chem. Soc. **141**, 2577–2588 (2019). 30676018 10.1021/jacs.8b12553

[23] G. V. Dubacheva, T. Curk, R. Auzély-Velty, D. Frenkel, R. P. Richter, Designing multivalent probes for tunable superselective targeting. Proc. Natl. Acad. Sci. U.S.A. **112**, 5579–5584 (2015). 25901321 10.1073/pnas.1500622112 PMC4426472

[24] M. Mammen, S.-K. Choi, G. M. Whitesides, Polyvalent interactions in biological systems: Implications for design and use of multivalent ligands and inhibitors. Angew. Chem. Int. Ed. Engl. **37**, 2754–2794 (1998). 29711117 10.1002/(SICI)1521-3773(19981102)37:20<2754::AID-ANIE2754>3.0.CO;2-3

[25] Y. Lu, G. Allegri, J. Huskens, Recruitment of receptors and ligands in a weakly multivalent system with omnipresent signatures of superselective binding. Small **19**, 2206596 (2023). 10.1002/smll.202206596 36876448

[26] N. J. Overeem, E. van der Vries, J. Huskens, A. Dynamic, Supramolecular view on the multivalent interaction between influenza virus and host cell. Small **17**, 2007214 (2021). 10.1002/smll.202007214 33682339

[27] M. D. Hollenberg, Mechanisms of receptor-mediated transmembrane signalling. Experientia **42**, 718–727 (1986). 2426135 10.1007/BF01941517

[28] A. L. Schwartz, Receptor cell biology: Receptor-mediated endocytosis. Pediatr. Res. **38**, 835–843 (1995). 8618782 10.1203/00006450-199512000-00003

[29] W. Morton, R. Vácha, S. Angioletti-Uberti, Valency of ligand-receptor binding from pair potentials. J. Chem. Theory Comput. **20**, 2901–2907 (2024). 38516954 10.1021/acs.jctc.4c00112 PMC11008093

[30] S. Bhatia, L. C. Camacho, R. Haag, Pathogen inhibition by multivalent ligand architectures. J. Am. Chem. Soc. **138**, 8654–8666 (2016). 27341003 10.1021/jacs.5b12950

[31] O. Rolland, C.-O. Turrin, A.-M. Caminade, J.-P. Majoral, Dendrimers and nanomedicine: Multivalency in action. New J. Chem. **33**, 1809–1824 (2009).

[32] L. Woythe, N. B. Tito, L. Albertazzi, A quantitative view on multivalent nanomedicine targeting. Adv. Drug Deliv. Rev. **169**, 1–21 (2021). 33264593 10.1016/j.addr.2020.11.010

[33] F. Xiu , Multivalent noncovalent interfacing and cross-linking of supramolecular tubes. Adv. Mater. **34**, 2105926 (2022). 10.1002/adma.202105926 34821422

[34] L. Albertazzi , Spatiotemporal control and superselectivity in supramolecular polymers using multivalency. Proc. Natl. Acad. Sci. U.S.A. **110**, 12203–12208 (2013). 23836666 10.1073/pnas.1303109110 PMC3725081

[35] S. Dhiman, A. Jain, M. Kumar, S. J. George, Adenosine-phosphate-fueled, temporally programmed supramolecular polymers with multiple transient states. J. Am. Chem. Soc. **139**, 16568–16575 (2017). 28845662 10.1021/jacs.7b07469

[36] H.-J. Schneider, Binding mechanisms in supramolecular complexes. Angew. Chem. Int. Ed. Engl. **48**, 3924–3977 (2009). 19415701 10.1002/anie.200802947

[37] C. Fasting , Multivalency as a chemical organization and action principle. Angew. Chem. Int. Ed. Engl. **51**, 10472–10498 (2012). 22952048 10.1002/anie.201201114

[38] L. Röglin, E. H. M. Lempens, E. W. Meijer, A. Synthetic, “Tour de force”: Well-defined multivalent and multimodal dendritic structures for biomedical applications. Angew. Chem. Int. Ed. Engl. **50**, 102–112 (2011). 21117109 10.1002/anie.201003968

[39] T. F. E. Paffen, A. J. P. Teunissen, T. F. A. de Greef, E. W. Meijer, Model-driven engineering of supramolecular buffering by multivalency. Proc. Natl. Acad. Sci. U.S.A. **114**, 12882–12887 (2017). 29158398 10.1073/pnas.1710993114 PMC5724267

[40] H. Bila, K. Paloja, V. Caroprese, A. Kononenko, M. M. Bastings, Multivalent pattern recognition through control of nano-spacing in low-valency super-selective materials. J. Am. Chem. Soc. **144**, 21576–21586 (2022). 36383954 10.1021/jacs.2c08529 PMC9716526

[41] L. Bartoš, M. Lund, R. Vácha, Enhanced diffusion through multivalency. Soft Matter **21**, 179–185 (2025). 39628400 10.1039/d4sm00778f PMC11615653

[42] Y. Cho, T. Christoff-Tempesta, S. J. Kaser, J. H. Ortony, Dynamics in supramolecular nanomaterials. Soft Matter **17**, 5850–5863 (2021). 34114584 10.1039/d1sm00047k

[43] D. Bochicchio, M. Salvalaglio, G. M. Pavan, Into the dynamics of a supramolecular polymer at submolecular resolution. Nat. Commun. **8**, 147 (2017). 28747661 10.1038/s41467-017-00189-0 PMC5529520

[44] J. H. Ortony , Internal dynamics of a supramolecular nanofibre. Nat. Mater. **13**, 812–816 (2014). 24859643 10.1038/nmat3979 PMC4110180

[45] L. Albertazzi , Probing exchange pathways in one-dimensional aggregates with super-resolution microscopy. Science **344**, 491–495 (2014). 24786073 10.1126/science.1250945

[46] A. Torchi, D. Bochicchio, G. M. Pavan, How the dynamics of a supramolecular polymer determines its dynamic adaptivity and stimuli-responsiveness: Structure–dynamics–property relationships from coarse-grained simulations. J. Phys. Chem. B. **122**, 4169–4178 (2018). 29543455 10.1021/acs.jpcb.8b00428

[47] L. Rijns, M. B. Baker, P. Y. W. Dankers, Using chemistry to recreate the complexity of the extracellular matrix: Guidelines for supramolecular hydrogel-cell interactions. J. Am. Chem. Soc. **146**, 17539–17558 (2024). 38888174 10.1021/jacs.4c02980 PMC11229007

[48] S. C. Yuan , Supramolecular motion enables chondrogenic bioactivity of a cyclic peptide mimetic of transforming growth factor-β1. J. Am. Chem. Soc. **146**, 21555–21567 (2024). 39054767 10.1021/jacs.4c05170

[49] G. Morgese , Anchoring supramolecular polymers to human red blood cells by combining dynamic covalent and non-covalent chemistries. Angew. Chem. Int. Ed. Engl. **59**, 17229–17233 (2020). 32584462 10.1002/anie.202006381 PMC7540258

[50] M. M. Bastings , Quantifying guest-host dynamics in supramolecular assemblies to analyze their robustness. Macromol. Biosci. **19**, 1800296 (2019). 10.1002/mabi.201800296 30511809

[51] Z. Álvarez , Bioactive scaffolds with enhanced supramolecular motion promote recovery from spinal cord injury. Science **374**, 848–856 (2021). 34762454 10.1126/science.abh3602 PMC8723833

[52] X. Xia, G. Zhang, M. Pica Ciamarra, Y. Jiao, R. Ni, The role of receptor uniformity in multivalent binding. JACS Au **3**, 1385–1391 (2023). 37234107 10.1021/jacsau.3c00052 PMC10207130

[53] D. Di Iorio, M. L. Verheijden, E. van der Vries, P. Jonkheijm, J. Huskens, Weak multivalent binding of influenza hemagglutinin nanoparticles at a sialoglycan-functionalized supported lipid bilayer. ACS Nano **13**, 3413–3423 (2019). 30844236 10.1021/acsnano.8b09410 PMC6439437

[54] D. D. Iorio, Y. Lu, J. Meulman, J. Huskens, Recruitment of receptors at supported lipid bilayers promoted by the multivalent binding of ligand-modified unilamellar vesicles. Chem. Sci. **11**, 3307–3315 (2020). 34122838 10.1039/d0sc00518e PMC8152591

[55] S. I. S. Hendrikse, R. Contreras-Montoya, A. V. Ellis, P. Thordarson, J. W. Steed, Biofunctionality with a twist: The importance of molecular organisation, handedness and configuration in synthetic biomaterial design. Chem. Soc. Rev. **51**, 28–42 (2022). 34846055 10.1039/d1cs00896j

[56] S. I. S. Hendrikse , Controlling and tuning the dynamic nature of supramolecular polymers in aqueous solutions. Chem. Commun. **53**, 2279–2282 (2017). 10.1039/c6cc10046e 28154855

[57] M. Kumar, J. N. S. Hanssen, S. Dhiman, Unveiling the liquid-liquid phase separation of Benzene-1, 3, 5-tricarboxamide in water. ChemSystemsChem **6**, e202400013 (2024).

[58] H. Duijs, M. Kumar, S. Dhiman, L. Su, Harnessing competitive interactions to regulate supramolecular “micelle-droplet-fiber” transition and reversibility in water. J. Am. Chem. Soc. **146**, 29759–29766 (2024). 39405510 10.1021/jacs.4c11285 PMC11528417

[59] J. N. S. Hanssen, S. Dhiman, Impact of subtle intermolecular interactions on the structure and dynamics of multicomponent supramolecular polymers. Chem. Commun. **59**, 13466–13469 (2023). 10.1039/d3cc04567f 37877229

[60] P. Thomas, T. G. Smart, “Receptor dynamics at the cell surface studied using functional tagging” in The Dynamic Synapse: Molecular Methods in Ionotropic Receptor Biology, J. T. Kittler, S. J. Moss, Eds. (CRC Press/Taylor & Francis, Boca Raton, FL, 2006), pp. 155–176.

[61] K. Jacobson, P. Liu, B. C. Lagerholm, The lateral organization and mobility of plasma membrane components. Cell **177**, 806–819 (2019). 31051105 10.1016/j.cell.2019.04.018 PMC6541401

[62] C. Scomparin, S. Lecuyer, M. Ferreira, T. Charitat, B. Tinland, Diffusion in supported lipid bilayers: Influence of substrate and preparation technique on the internal dynamics. Eur. Phys. J. E **28**, 211–220 (2009). 19101741 10.1140/epje/i2008-10407-3

[63] G. Lindblom, G. Orädd, Lipid lateral diffusion and membrane heterogeneity. Biochim. Biophys. Acta. **1788**, 234–244 (2009). 18805393 10.1016/j.bbamem.2008.08.016

[64] D. Morzy, M. Bastings, Significance of receptor mobility in multivalent binding on lipid membranes. Angew. Chem. Int. Ed. Engl. **134**, e202114167 (2022). 10.1002/anie.202114167 PMC9303963 34982497

[65] M. Ester, H.-P. Kriegel, J. Sander, X. Xu, A density-based algorithm for discovering clusters in large spatial databases with noise. kdd **96**, 226–231 (1996).

[66] M. Crippa, C. Perego, A. L. de Marco, G. M. Pavan, Molecular communications in complex systems of dynamic supramolecular polymers. Nat. Commun. **13**, 2162 (2022). 35443756 10.1038/s41467-022-29804-5 PMC9021206

[67] D. Bochicchio, G. M. Pavan, From cooperative self-assembly to water-soluble supramolecular polymers using coarse-grained simulations. ACS Nano **11**, 1000–1011 (2017). 27992720 10.1021/acsnano.6b07628

[68] A. Laio, M. Parrinello, Escaping free-energy minima. Proc. Natl. Acad. Sci. U.S.A. **99**, 12562–12566 (2002). 12271136 10.1073/pnas.202427399 PMC130499

[69] I. Gimondi, G. A. Tribello, M. Salvalaglio, Building maps in collective variable space. J. Chem. Phys. **149**, 104104 (2018). 30219018 10.1063/1.5027528

[70] L. Leanza , Into the dynamics of rotaxanes at atomistic resolution. Chem. Sci. **14**, 6716–6729 (2023). 37350834 10.1039/d3sc01593a PMC10283497

[71] R. Cappabianca, P. De Angelis, A. Cardellini, E. Chiavazzo, P. Asinari, Assembling biocompatible polymers on gold nanoparticles: Toward a rational design of particle shape by molecular dynamics. ACS Omega **7**, 42292–42303 (2022). 36440134 10.1021/acsomega.2c05218 PMC9686196

[72] F. J. Martinez-Veracoechea, D. Frenkel, Designing super selectivity in multivalent nano-particle binding. Proc. Natl. Acad. Sci. U.S.A. **108**, 10963–10968 (2011). 21690358 10.1073/pnas.1105351108 PMC3131366

[73] V. Bayle , Single-particle tracking photoactivated localization microscopy of membrane proteins in living plant tissues. Nat. Protoc. **16**, 1600–1628 (2021). 33627844 10.1038/s41596-020-00471-4

[74] K. Kaygisiz , Data-mining unveils structure–property–activity correlation of viral infectivity enhancing self-assembling peptides. Nat. Commun. **14**, 5121 (2023). 37612273 10.1038/s41467-023-40663-6 PMC10447463

[75] M. E. J. Vleugels , Antibody-recruiting surfaces using adaptive multicomponent supramolecular copolymers. Biomacromolecules **26**, 2971–2985 (2025). 40202813 10.1021/acs.biomac.5c00043 PMC12076489

