# Supplementary material for: Reciprocity in dynamics of supramolecular biosystems for the clustering of ligands and receptors
Source: Proc Natl Acad Sci U S A. 2025 Sep 8;122(37):e2500686122. doi: 10.1073/pnas.2500686122 (PMC12452917; doi:10.1073/pnas.2500686122)
Supplement: Supplementary file 1 — Appendix 01 (PDF) [file pnas.2500686122.sapp.pdf]

PNAS

Supporting Information for

Reciprocity in dynamics of supramolecular biosystems for the clustering of ligands and receptors

Shikha Dhiman[1,2,3*†], Marle E. J. Vleugels[1,2†], Richard A. J. Post[1,5], Martina Crippa[6], Annalisa Cardellini[7], Esmee de Korver[1,2], Lu Su[4], Anja R. A. Palmans,[3] Giovanni M. Pavan[6,7], Remco W. van der Hofstad[1,5], Lorenzo Albertazzi[1,8], and E. W. Meijer[1,2,9*]

[1] Institute for Complex Molecular Systems, Eindhoven University of Technology, Eindhoven 5600 MB, The Netherlands
[2] Laboratory of Macromolecular and Organic Chemistry, Eindhoven University of Technology, Eindhoven 5600 MB, The Netherlands
[3] Department of Chemistry, Johannes Gutenberg University Mainz, Duesbergweg 10-14, D-55128, Mainz, Germany
[4] Division of Biotherapeutics, Leiden Academic Centre for Drug Research (LACDR), Leiden University, Einsteinweg 55, 2333 CC Leiden, The Netherlands.
[5] Department of Epidemiology and Biostatistics, Erasmus University Medical Center, Rotterdam 3015 GD, The Netherlands
[6] Department of Applied Science and Technology, Politecnico di Torino, Corso Duca degli Abruzzi 24, 10129 Torino, Italy
[7] Department of Innovative Technologies, University of Applied Sciences and Arts of Southern Switzerland, Polo Universitario Lugano, Campus Est, Via la Santa 1, 6962 Lugano-Viganello, Switzerland
[8] Department of Biomedical Engineering, Eindhoven University of Technology, 5600 MB Eindhoven, Netherlands
[9] School of Chemistry and RNA Institute, University of New South Wales, Sydney, NSW 2052, Australia

*Shikha Dhiman, *E.W. Meijer
Email: shikha.dhiman@uni-mainz.de , e.w.meijer@tue.nl

This PDF file includes:

Supporting text
Figures S1 to S41
Legends for Movie S1
SI References

Other supporting materials for this manuscript include the following:

Movies S1: TIRFM video showing zipping effect

Supporting Information Text

Synthesis and experiments: All reagents and chemicals were obtained from commercial sources at the highest purity available and used without further purification unless stated otherwise. All solvents were of AR quality. 1,2-Dioleoyl-sn-glycero-3-phosphocholine (DOPC), 1,2-dioleoyl-sn-glycero-3-phosphoethanolamine-N-(cap biotinyl), sodium salt (DOPEBio), 1,2-dipalmitoyl-sn-glycero-3-phosphocholine (DPPC), were obtained from Avanti Polar Lipids. Texas Red™ 1,2-Dihexadecanoyl-sn-Glycero-3-Phosphoethanolamine, Triethylammonium Salt (TRDHPE) and Streptavidin conjugated with Aexa Fluor 488 were obtained from Thermofischer. Streptavidin from Streptomyces avidinii was obtained from Merck. Streptavidin conjugated with Abberior CAGE 635 were purchased from Abberior. DNA oligomers were purchased from IDTDNA. M-Slide 8 Well plate for total internal reflection fluorescence (TIRF) microscopy were obtained from Ibidi. Water was purified on an EMD Millipore Milli-Q Integral Water Purification System. SiO_2-coated QCM sensors (QSX 303) were purchased from Biolin Scientific. Reactions were followed by thin-layer chromatography (precoated 0.25 mm, 60-F254 silica gel plates from Merck). Dry solvents were obtained with an MBRAUN Solvent Purification System (MB-SPS). Automated column chromatography was performed on a Biotage Isolera using Biotage® SNAP-KP SIL cartridges.

Nuclear Magnetic Resonance (NMR): NMR spectra were recorded on Bruker Avance III HD spectrometer at 298 K (400 MHz for ^{1}H-NMR). Deuterated solvents were used are indicated in each case. Chemical shifts are reported in ppm downfield from TMS at room temperature. Abbreviations used for splitting patterns are s = singlet, t = triplet, q = quartet, m = multiplet and br = broad.

Liquid chromatography mass spectrometry (LC-MS): LCMS was performed on a system consisting of the following components: Shimadzu SCL-10A VP system controller with Shimadzu LC-10AD VP liquid chromatography pumps (with an Alltima C18 3 u (50 × 2.1 mm) reversed-phase column and gradients of water–acetonitrile supplemented with 0.1 % formic acid, a Shimadzu DGU 20A3 prominence degasser, a Thermo Finnigan surveyor auto sampler, a Thermo Finnigan surveyor PDA detector and a Thermo Scientific LCQ Fleet. Gradients were run from 5 % MeCN to 100 % MeCN over a 15-minute period.

Matrix assisted laser absorption/ionization mass time of flight (MALDI-TOF) spectra were obtained on a Bruker Autoflex Speed. A-cyano-4-hydroxycinnamic acid (CHCA) and trans-2-[3-(4-tert-butylphenyl)-2-methyl-2-propenylidene]malononitrile (DCBT) were used as matrix. All samples were dissolved in tetrahydrofuran.

High-performance liquid chromatography (HPLC): HPLC-PDA/MS was performed using a Shimadzu LC-10 AD VP series HPLC coupled to a diode array detector (Finnigan Surveyor PDA Plus detector, Thermo Electron Corporation) and an Ion-Trap (LCQ Fleet, Thermo Scientific). HPLC-analyses were performed using a Alltech Alltima HP C18 3µ column using an injection volume of 2 µL, a flow rate of 0.2 mL min-1 and a gradient (5 % to 100 % in 10 min, held at 100 % for a further 3 min) of MeCN in H2O (both containing 0.1 % formic acid) at 298 K.

Reversed Phase-Medium pressure liquid chromatography (RP-MPLC): Preparative RP-MPLC (MeCN/H2O) was performed using a Biotage One coupled to a PDA detector and a Biotage SNAP-KP-C18-HS column.

Total internal reflection fluorescence (TIRF) microscopy: TIRF images were acquired with a Nikon N-STORM microscopy system. Sample was excited using 561 nm laser. Fluorescence was collected using a Nikon×100, 1.4NA oil immersion objective and passed through a quad-band pass dichroic filter (97335 Nikon). Images were recorded with an EMCCD camera (ixon3, Andor, pixel size 0.17 µm). The samples were imaged in a µ-Slide 8 Well plate with No. 1.5 coverslip bottom suitable for microscopy.

STORM imaging: STORM images were acquired using a Nikon N-STORM system configured for total internal reflection fluorescence (TIRF) imaging. Excitation inclination was tuned to maximize the signal-to-noise ration of the glass-absorbed fibers. Cy3- and Cy-5 labeled samples were

illuminated by the 561nm and 647nm laser lines built into the microscope. No activation UV light was employed. Fluorescence was collected by means of a Nikon 100x, 1.4NA oil immersion objective and passed through a quad-band pass dichroic filter (97335 Nikon). All timelapses were recorded onto a 128x128pixel region (pixel size 0.17µm) of a EMCCD camera (ixon3, Andor). Upto 10,000 frames for every channel were acquired sequentially. STORM movies were analyzed with the STORM module of the NIS element Nikon software.

QCM-D measurements: *QCM-D* was performed on a Qsense Analyser from Biolin Scientific. SiO_2-coated sensors (QSX 303) were used for measurements. The experiment was done with four parallel flow chambers with controlled flow rate using Ismatec peristaltic pumps.
Dynamic Light Scattering (DLS) measurements: DLS were performed using a Malvern µV Zetasizer equipped with an 830 nm laser and a scattering angle of 90° at a temperature of 25 °C. Samples were measured in Sarstedt UV-transparent disposable cuvettes with a pathlength of 10 x 2 mm. Measurements were analyzed using Zetasizer software.

MATLAB cluster analysis: The Thunderstorm plugin of ImageJ (Fiji) software was employed to conduct data analysis on the STORM time series. This involved using Gaussian fitting to detect blinking chromophores within the conventional microscopic image movie, generating a list of localizations. A custom MATLAB script, adapted from a previously used version for STORM image analysis of BTA fibers, was utilized to analyze this list of localizations.[1] The script employs DBSCAN, a clustering algorithm commonly used in machine learning and data mining, to identify dense regions of data points and group them together as clusters.[2,3] DBSCAN can identify clusters of arbitrary shapes and outliers (noise) within the data, and requires two key parameters, namely the radius (dbr or r) and minimum number of points required to form a dense region (dbn or n). The script was built on previously STORM imaging of 1D supramolecular polymers reported.

SPT-PALM: Super-resolution images were reconstructed using Oxford Nanoimager software Nimos 1.16. Briefly, a two-dimensional Gaussian was fitted to individual fluorescence spots with at least 300 photons per frame to identify single molecules. Single-particle tracking to obtain MR trajectories was performed with the same software and the following parameters: maximum frame gap, 3; maximum distance between frames, 0.6 µm; exclusion radius, 1.2 µm; and a minimum of two steps per trajectory. ONI software was used to obtain the trajectories.

Synthesis protocol: BTA (or BTA-$(OH)_3$),[4] BTA-Ba,[5] BTA-Bio,[6] BTA-Cy3,[1] BTA-Cy5,[1] BTA-DNA,[7] monovalent mUPy,[8] bivalent bUPy,[9] UPy-Cy3[10] H_2N-C_{12}-EO_4-N_3,[1] and H_2N-C_{12}-EO_4-OBn[4] were synthesised according to previously reported procedures.

Synthesis of UPyBio:

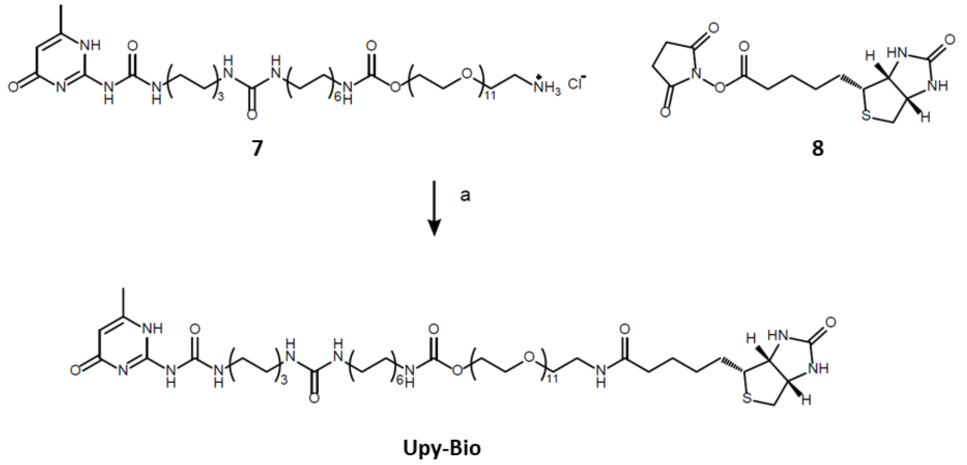

Figure S1. Synthesis of UPyBio. (A) Biotine N-hydroxysuccinimide ester, DIPEA, $CHCl_3$, DMF, RT, 1h, 83%.

Synthesis of UPy-Bio; (37-Oxo-41-((3aR,4R,6aS)-2-oxohexahydro-1H-thieno[3,4-d]imidazol-4-yl)-3,6,9,12,15,18,21,24,27,30,33-undecaoxa-36-azahentetracontyl (12-(3-(6-(3-(6-methyl-4-oxo-1,4-dihydropyrimidin-2-yl)ureido)hexyl)ureido)dodecyl)carbamate): Amine compound **7** (i.e. 1-((6-methyl-4-oxo-1,4-dihydropyrimidin-2-yl)amino)-1,10,25-trioxo-26,29,32,35,38,41,44,47,50,53,56, 59-dodecaoxa-2,9,11,24-tetraazahenhexacontan-61-aminiumchloride was prepared as according to Isja de Feijter et al.[11]

The amine (**7**, 22 mg, 0.020 mmol) and N,N-diisopropylethylamine (DIPEA, 26 mg, 0.2 mmol, 10 mol eq.) were dissolved in $CHCl_3$ (250 µL). A solution of (+)-biotin N-hydroxysuccinimide (8, 8.9 mg, 0.026 mmol, 1.3 eq) in DMF (200 µL) was added, and the solution was stirred at room temperature for 1h. The reaction mixture was precipitated in diisopropylether (10 mL) filtrated, washed with diisopropylether and dried. The white solid was further purified with RF-MPLC using a gradient of water and MeCN mixtures with increasing MeCN-content. The fractions with pure product were pooled and lyophilized giving a 21.5 mg (83%) yield of UPy-Bio.

^{1}H NMR (400 MHz, Chloroform-d) δ 13.14 (s, 1H), 11.84 (s, 1H), 10.06 (s, 1H), 6.75 (s, 1H), 5.90 (s, 1H, br) 5.84 (s, 2H), 5.21 (s, 1H), 5.05 (s, 1H), 4.90 (s, 1H), 4.51 (m, 1H), 4.32 (m, 1H), 4.21 (m, 2H), 3.77 –3.59 (m, 42H), 3.57 (t, J = 5.1 Hz, 2H), 3.44 (q, J = 5.2 Hz, 2H), 3.25 (d, J = 6.3 Hz, 2H), 3.15 (q, J = 6.9 Hz, 7H), 2.92 (dd, J = 12.8, 5.0 Hz, 1H), 2.74 (d, J = 12.8 Hz, 1H), 2.22 (d, J = 10.7 Hz, 5H), 1.97 – 1.54 (m, 6H), 1.46 (m, 8H), 1.37 (s, 4H), 1.26 (m, 16H).

ESI-MS: m/z Calc. for $C_{60}H_{110}N_{10}O_{18}S$ 1291.65; exact mass 1290.77. Obs. $[M+H]^+$ 1291.58, $[M+Na]^+$ 1313.50, $[M+2H]^{2+}$ 646.58 and $[M-H]^-$ 1290.17.

Synthesis of BTA-Ba-Cy5 and BTA-Ba-Cy3:

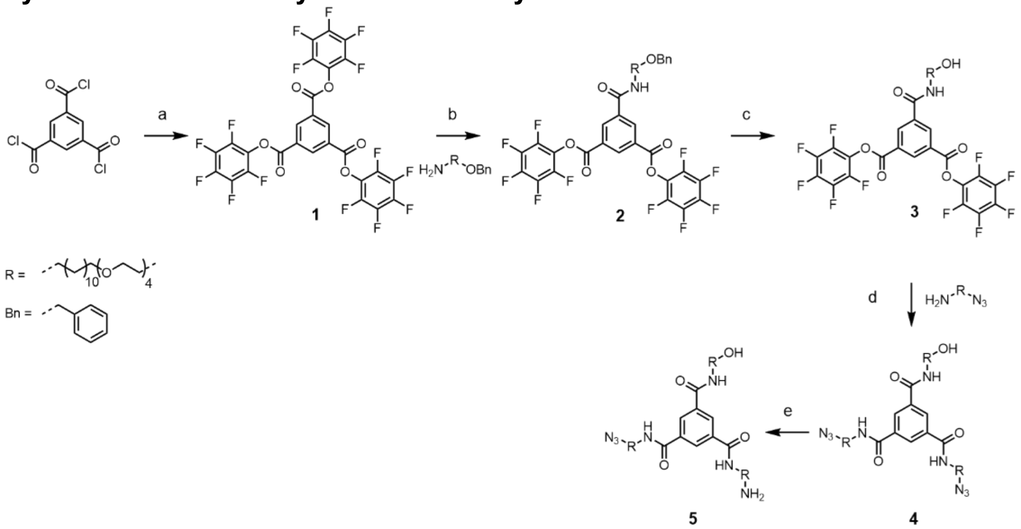

Figure S2. Synthetic route towards orthogonally reactive trifunctional BTA monomer (**5**). Reaction conditions: (A) DCM, DIPEA, 0°C – RT, 94% (B) DCM, TEA, 0°C, 73%* (C) EtOAc, Pd/C, H_2, 97% (D) DCM, TEA, 75% (E) THF/H_2O, PPh_3, 50°C, 48%. (*) conversion for the first statistical aminolysis reaction was 73%, considering the maximum possible theoretical yield of 37% for the statistical reaction.

Tris(perfluorophenyl) benzene-1,3,5-tricarboxylate (1): Pentafluorophenol (2.76 g, 14.98 mmol, 4 eq) and anhydrous DCM (15 mL) were added to a dried round bottom flask and stirred under nitrogen until complete dissolution of the pentafluorophenol. An additional funnel was filled with diisopropylethylamine (DIPEA, 2 mL, 10.36 mmol, 2.75 eq) and anhydrous DCM (10 mL), which was added dropwise to the reaction mixture. After cooling down the reaction mixture with an ice bath, 1,3,5-benzenetricarbonyl trichloride (1 g, 3.77 mmol, 1 eq) was dissolved in 15 mL anhydrous DCM and added dropwise into the reaction mixture. The reaction mixture was removed from the ice bath after 30 minutes and allowed to stir at room temperature overnight. TLC (run in DCM) showed reaction completion ($R_{f,prod}$ = 0.9, $R_{f,PFP}$ = 0.5). The reaction mixture was impregnated with silica (7 g) and passed through a silica bed with DCM. The solvent was removed in vacuo, yielding the product as pure white solid crystals (2.51 g, 94%)

^{1}H-NMR (400 MHz, $CDCl_3$) δ 9.29 (s, 3H).

^{19}F-NMR (375 MHz, $CDCl_3$) = δ -151.97 – -152.20 (m), -156.22 (t, J = 21.7 Hz), -161.15 – -161.45 (m).

Bis(perfluorophenyl)5-((1-phenyl-2,5,8,11,14-pentaoxahexacosan-26-yl)carbamoyl) isophthalate (2): Tris(perfluorophenyl) benzene-1,3,5-tricarboxylate (**1**) (1.51 g, 2.04 mmol, 1 eq) was dissolved in 7.5 mL anhydrous DCM, rigorously stirred under argon flow, and placed in an ice bath. TEA (1.28 mL, 9.16 mmol, 4.5 eq) was added dropwise. H_2N-C_{12}-EO_4-OBn (952.44 mg, 9.16 mmol, 1 eq) was dissolved in 3 mL anhydrous DCM and slowly added dropwise using an additional funnel. The mixture was stirred for about 2 h, and full conversion was checked with ^{19}F-NMR and TLC (eluents ethyl acetate/DCM 50/50, Rf_{start} = 1, Rf = 0.9 monosubstituted product (2), R_f = 0.5 disubstituted compound, R_f = 0.1 trisubstituted compound. The solvent was removed in vacuo and column chromatography was performed using a Biotage Isolera One column machine (Biotage

Sfär 50 g prepacked silica column) by flushing with a gradient of DCM/ethyl acetate of 100/0 – 10/90 v/v. Fractions were analyzed, and solvent removed in vacuo (0.477 g, 73%).

^{1}H-NMR (400 MHz, $CDCl_3$) δ 9.09 (s, 1H), 8.88 (S, 2H), 7.33 (d, *J* = 4.4 Hz, 4H), 6.37 (d, *J* = 5.7 Hz, 1H), 4.56 (s, 2H), 3.73 – 3.61 (m, 14H), 3.60 – 3.48 (m, 4H), 3.43 (t, *J* = 6.8 Hz, 2H), 1.67 (t, 2H), 1.56 (t, *J* = 6.9 Hz, 2H), 1.27 (s, 14H).

^{19}F-NMR (375 MHz, $CDCl_3$) δ -152.18 (d), -156.64 (t, *J* = 2.6 Hz), -161.51 (td, *J* = 21.3, 4.0 Hz), -163.20 (d, *J* = 6.5 Hz), -164.19 (td, *J* = 20.4 Hz), -169.20 (t).

Bis(perfluorophenyl)5-((1-hydroxy-3,6,9,12-tetraoxatetracosan-24-yl)carbamoyl) isophthalate (3): Bis(perfluorophenyl)5-((1-phenyl-2,5,8,11,14-pentaoxahexacosan-26-yl)carbamoyl)isophthalate (**2**) (149 mg, 150 µmol, 1 eq) was dissolved in ethyl acetate (7.5 mL) in a round-bottom flask and degassed with N_2 (g) for 15 minutes. Subsequently, a catalytic amount of Pd/C (25.6 mg, 0.16 eq, 10% Pd/C) was added, and a balloon filled with H_2 (g) was connected after carefully releasing the N_2 gas to ensure a H_2 atmosphere. The reaction mixture was stirred overnight at room temperature, after which the conversion was checked with ^{1}H-NMR in $CDCl_3$. Subsequently, the reaction mixture was filtered over a glass filter with celite. Ethyl acetate was used to wash the sample thrice. The filtrate was concentrated in vacuo and afforded a yellowish oil (132 mg, 97%).

^{1}H-NMR (400 MHz, $CDCl_3$) δ 9.10 (s, 1H), 8.89 (s, 2H), 6.39 (d, *J* = 6.2 Hz, 1H), 3.72 (dd, *J* = 5.5, 3.6 Hz, 2H), 3.69 – 3.57 (m, 14H), 3.53 (dt, *J* = 13.6, 6.6 Hz, 2H), 3.44 (t, *J* = 6.8 Hz, 2H), 1.66 (t, *J* = 7.4 Hz, 4H), 1.57 (t, *J* = 7.0 Hz, 2H), 1.26 (d, *J* = 3.4 Hz, 14H).

N1,N3-Bis(1-azido-3,6,9,12-tetraoxatetracosan-24-yl)-N5-(1-hydroxy-3,6,9,12-tetraoxatetracosan-24-yl)benzene-1,3,5-tricarboxamide (4): Bis(perfluorophenyl)5-((1-hydroxy-3,6,9,12-tetraoxatetracosan-24-yl)carbamoyl)isophthalate (**3**) (777.90 mg, 862.62 µmol, 1 eq) was dissolved in 20 mL anhydrous DCM in a 100 mL round bottom flask, stirred under argon and placed in an ice bath. TEA (0.6 ml, 4.5 eq) was added dropwise. H_2N-C_{12}-EO_4-N_3 (938 mg, 2.33 mmol, 2.7 eq) was dissolved in 6 mL dry DCM and added slowly dropwise using an additional funnel. The mixture was stirred at room temperature for 4 h and checked for reaction completion using ^{19}F-NMR ($CDCl_3$) and TLC (DCM/MeOH 95/5, $R_{f,prod}$ = 0.3, $R_{f,amine}$ = 0.1, stained with $KMnO_4$). The solvent was removed in vacuo. The excess pentafluorophenol was removed by extracting the organic layer thrice, by adding 60 mL of DCM and 15 mL of 0.1 M NaOH. Then, the aqueous layer was back extracted twice with 50 mL DCM. All organic layers were combined and washed twice with 100 mL brine. $MgSO_4$ was added to the organic layer to remove the last traces of water. The product was concentrated in vacuo and coevaporated trice with $CHCl_3$ to afford a yellow clear oil. The reaction mixture was purified by column chromatography using a Biotage Isolera One column machine (Biotage Sfär 50 g prepacked silica column) with a slow gradient of DCM/methanol 100/0 – 95/5. The fractions were analyzed, and the solvent was removed in vacuo, which yielded a sticky solid (867.5 mg, 75%).

^{1}H-NMR (400 MHz, $CDCl_3$) δ 8.36 (s, 3H), 6.56 (t, *J* = 5.5 Hz, 3H), 3.76 – 3.69 (m, 2H), 3.69 – 3.54 (m, 42H), 3.49 – 3.42 (m, 12H), 3.38 (t, *J* = 5.1 Hz, 4H), 1.62 (t, *J* = 7.2 Hz, 4H), 1.55 (t, *J* = 6.4 Hz, 4H), 1.44 – 1.20 (m, 48H).

^{13}C-NMR (100 MHz, $CDCl_3$) δ 165.67, 135.27, 128.01, 73.22 – 69.37 (m), 61.75, 53.43, 50.70, 40.38, 30.45 – 24.59 (m).

LC-MS: Rt = 6.22 min, calculated m/z = 1337.94, observed m/z = 1338.75 $[M+H]^+$, 669.83 $[M+2H]^{2+}$

N1-(1-Amino-3,6,9,12-tetraoxatetracosan-24-yl)-N3-(1-azido-3,6,9,12-tetraoxatetracosan-24-yl)-N5-(1-hydroxy-3,6,9,12-tetraoxatetracosan-24-yl)benzene-1,3,5-tricarboxamide (5): N1,N3-Bis(1-azido-3,6,9,12-tetraoxatetracosan-24-yl)-N5-(1-hydroxy-3,6,9,12-tetraoxatetracosan-24-yl)benzene-1,3,5-tricarboxamide (**4**) (867 mg, 645 µmol, 1 eq) and triphenylphosphine (170.1 mg, 649 µmol, 1eq) were dissolved in THF (40 mL) in a round bottom flask. The mixture was stirred under an argon atmosphere at 50 °C for 24 hours. Subsequently, H_2O (14.5 mL, 804 µmol, 1.24 eq) was added to the mixture and stirred for 24 hours at the same conditions. The reaction conversion was checked with TLC (90% (MeOH/TEA/$CHCl_3$ 10/0.5/89.5) with 10% $CHCl_3$, $R_{f,\text{twice reduced prod}}$ = 0.0, $R_{f,\text{prod}}$ = 0.2, $R_{f,\text{start}}$ = 0.5). The solution was concentrated in vacuo and compound (5) was purified by column chromatography with a Biotage Isolera One column machine (Biotage Sfär 50 g prepacked silica column). By flushing with 100% ethyl acetate the triphenylphosphine oxide was removed. Then the solvent mixture was switched to (MeOH/TEA/$CHCl_3$ 10/0.5/89.5 v/v) / $CHCl_3$ with a gradient 30/70 – 90/10 v/v to remove the starting compound and product. Lastly the column was flushed with MeOH/IPA/$CHCl_3$ 10/2.5/87.5 v/v to flush the double reduced side product. The product (5) was obtained as an oil (410 mg, 48%).

^{1}H-NMR (400 MHz, $CDCl_3$) δ = 8.40 (s, 3H), 6.97 (t, 3H), 3.74 – 3.69 (m, 2H), 3.68 – 3.50 (m, 42H), 3.44 (t, *J* = 4.4 Hz, 12H), 3.38 (t, *J* = 5.1 Hz, 2H), 2.87 (t, *J* = 7.0 Hz, 2H), 1.61 (t, *J* = 6.9 Hz, 6H), 1.54 (t, *J* = 4.6 Hz, 6H), 1.42 – 1.18 (m, 48H).

^{13}C-NMR (100 MHz, $CDCl_3$) δ 165.90, 135.22, 128.22, 73.22 – 68.63 (m), 61.64, 50.69, 41.50, 40.37, 30.35 – 25.76 (m).

LC-MS: Rt = 5.17 min, calculated mass 1311.95 m/z, observed m/z = 1312.92 $[M+H]^+$, 657 $[M+H]^{2+}$, 876 $[2M+3H]^{3+}$

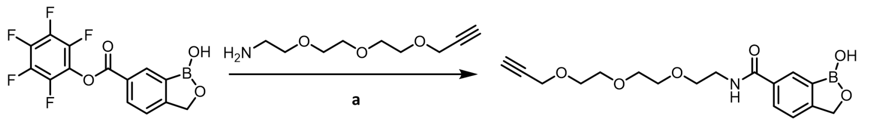

6 7

Figure S3. Synthetic route of Ba-alkyne (7). (A) DCM, RT, 98%.

1-Hydroxy-N-(2-(2-(2-(prop-2-yn-1-yloxy)ethoxy)ethoxy)ethyl)-1,3-dihydrobenzo[c][1,2] oxaborole-6-carboxamide (Ba-alkyne, (7)): Perfluorophenyl 1-hydroxy-1,3-dihydrobenzo[c][1,2]oxaborole-6-carboxylate (PFP-Ba (**6**), 100.4 mg, 291.86 µmol, 1 eq) was stirred with 1 mL DCM under an argon atmosphere. Triethylamine (0.17 mL, 1.21 mmol, 4.14 eq) was added to the turbid mixture which became clear within one minute. A solution of OEG_3-alkyne-NH_2 (109.3 mg, 583.72 µmol, 2 eq) in 2 mL DCM was added dropwise to the mixture and stirred overnight at room temperature. A TLC analysis (eluents $CHCl_3$/MeOH/acetic acid 90/9/1 v/v, R_f = 0.5, stained with $KMnO_4$) confirmed conversion, and the solvent was removed in vacuo. The crude product was purified with column chromatography using a Biotage Isolera One column machine (Biotage Sfär 25 g prepacked silica column) by flushing with a solvent mixture of $CHCl_3$/MeOH/acetic acid 90/9/1 v/v. The product was co-evaporated thrice with $CHCl_3$. Yield: 100.3 mg (98 %).

^{1}H NMR (400 MHz, DMSO-d_6) δ 9.29 (s, 1H), 8.51 (t, J = 5.6 Hz, 1H), 8.22 (s, 1H), 7.93 (dd, J = 8.0, 1.7 Hz, 1H), 7.48 (d, J = 8.0 Hz, 1H), 5.04 (s, 2H), 4.12 (d, J = 2.4 Hz, 2H), 3.54 (d, J = 5.3 Hz, 8H), 3.46 – 3.38 (m, 4H), 3.17 (s, 1H).

^{13}C NMR (100 MHz, DMSO) δ 167.07, 157.19, 133.88, 130.06 (d, J = 23.7 Hz), 121.68, 80.78, 79.64, 77.56, 70.68 – 68.58 (m), 57.94.

^{19}F NMR (375 MHz, DMSO) δ -162.05 (d, J = 22.7 Hz), -165.63 (t, J = 22.9 Hz), -172.44.
LC-MS: Rt = 3.76 min, calculated m/z = 347.15, observed m/z = 348.17 $[M+H]^+$.

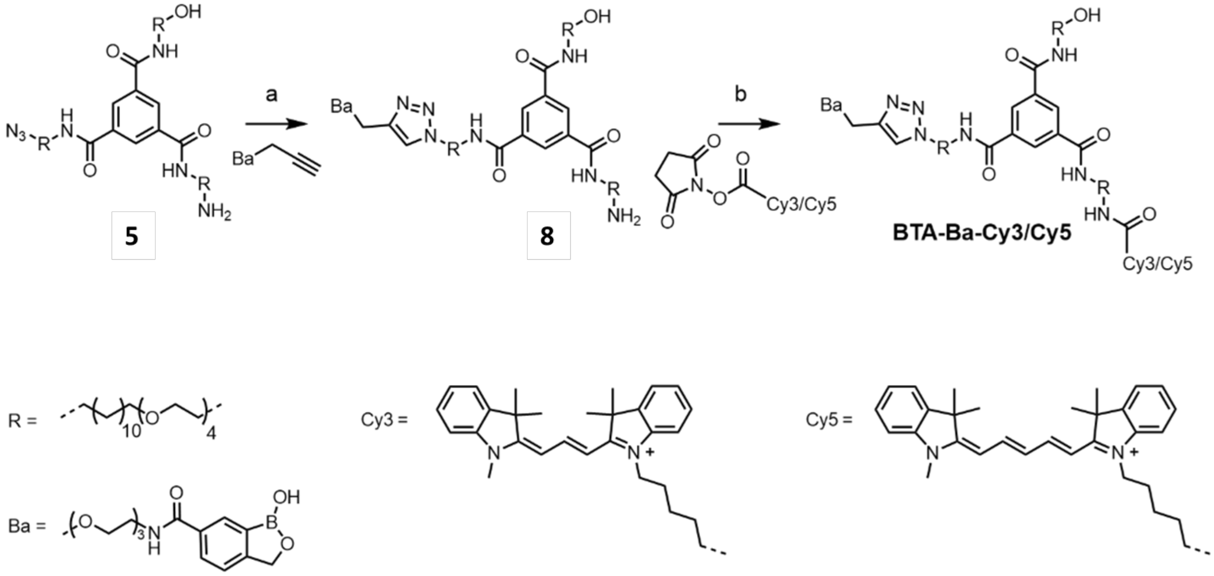

Figure S4. Synthetic route towards trackable monomers BTA-Ba-Cy3/Cy5. (A) $CuSO_4$, NaAsc, Bim(Py)$_2$, aminoguanidine, DMF/H_2O, quant. (B) DMSO, TEA, 31% (BTA-Ba-Cy3), 14% (BTA-Ba-Cy5).

N1-(1-Amino-3,6,9,12-tetraoxatetracosan-24-yl)-N3-(1-(4-(1-(1-hydroxy-1,3-dihydrobenzo[c][1,2]oxaborol-6-yl)-1-oxo-5,8,11-trioxa-2-azadodecan-12-yl)-1H-1,2,3-triazol-1-yl)-3,6,9,12-tetraoxatetracosan-24-yl)-N5-(1-hydroxy-3,6,9,12-tetraoxatetracosan-24-yl)benzene-1,3,5-tricarboxamide (8): Four reaction cocktails were prepared for the CuAAC click reaction; $CuSO_4$ (50 mM) in MQ, aminoguanidine (200 mM) in MQ, Bim(Py)$_2$ (30 mM) in DMF, and NaAsc (200 mM) in MQ. In this sequence, 200 µL of each cocktail was added to a small sample vial which resulted a coloring sequence; blue, green, and brown, respectively. This brown solution was added to a small sample vial containing N1-(1-amino-3,6,9,12-tetraoxatetracosan-24-yl)-N3-(1-azido-3,6,9,12-tetraoxatetracosan-24-yl)-N5-(1-hydroxy-3,6,9,12-tetraoxatetracosan-24-yl)benzene-1,3,5-tricarboxamide (**5**) (40 mg, 30.47 µmol, 1 eq) and alkyne-Ba (**7**) (12.7 mg, 36.56 µmol, 1.2 eq) in 400 µL DMF. The vial was stirred at room temperature overnight, and LC-MS analysis confirmed reaction conversion. The brown solution was passed through a glass filter filled with 3 cm of silica and flushed with $CHCl_3$/MeOH/IPA 68/30/2 v/v. The product fraction was verified with LC-MS, concentrated in vacuo, dissolved again in water, and freeze-dried to obtain the product as brownish sticky solid (60 mg, ~100%)

LC-MS: Rt = 5.89 min, calculated m/z = 1659.10, observed m/z = 1660.17 $[M+H]^{+}$, 830.75 $[M+2H]^{2+}$, 554.33 $[M+3H]^{3+}$.

BTA-Ba-Cy3: N1-(1-Amino-3,6,9,12-tetraoxatetracosan-24-yl)-N3-(1-(4-(1-(1-hydroxy-1,3 dihydrobenzo[c][1,2]oxaborol-6-yl)-1-oxo-5,8,11-trioxa-2-azadodecan-12-yl)-1H-1,2,3-triazol-1-yl)-3,6,9,12-tetraoxatetracosan-24-yl)-N5-(1-hydroxy-3,6,9,12-tetraoxatetracosan-24-yl)benzene-1,3,5-tricarboxamide (8) (10.30 mg, 6.20 µmol, 1 eq), NHS-coupled cyanine-3 dye (3.44 mg, 6.20 µmol, 1 eq), TEA (300 µL, 24.82 µmol, 4 eq) were dissolved in 300 µL DMSO in a sample vial. The reaction was stirred overnight at room temperature shielded from light. The reaction mixture was precipitated in 30 mL cold heptane/ether 20/80 v/v, incubated in the fridge for 30 minutes, and centrifugated (15 min, 3000 RPM). The supernatant heptane/ether layer was removed

carefully. The bright pink pellet was dissolved in water/can 80/20 and lyophilized to remove the remaining solvents. The crude compound was dissolved in 2 mL $CHCl_3$/MeOH 90/10 v/v and solvent loaded onto a normal phase column using a Biotage Isolera One column machine (Biotage Sfär 6 g prepacked silica column) with a gradient of $CHCl_3$/MeOH/isopropylamine 100/0/0 to 87.5/10/2.5 v/v. The fractions were analyzed by LC-MS (gradient of 50 to 100 % H_2O), concentrated in vacuo, dissolved again in water, and freeze-dried to obtain the bright pink product (4 mg, 30.5%) . LC-MS (50-100% gradient): Rt = 3.38 min, calculated m/z 2098.38, calculated m/z internal ester 2080.37, observed m/z = 1041.17 [internal ester+H]$^{2+}$, 694.33 [internal ester+2H]$^{3+}$

BTA-Ba-Cy5: N1-(1-Amino-3,6,9,12-tetraoxatetracosan-24-yl)-N3-(1-(4-(1-(1-hydroxy-1,3 dihydrobenzo[c][1,2]oxaborol-6-yl)-1-oxo-5,8,11-trioxa-2-azadodecan-12-yl)-1H-1,2,3-triazol-1-yl)-3,6,9,12-tetraoxatetracosan-24-yl)-N5-(1-hydroxy-3,6,9,12-tetraoxatetracosan-24-yl)benzene-1,3,5-tricarboxamide (**8**) (13.68 mg, 8.24 µmol, 1.1 eq), NHS-coupled cyanine-5 dye (5 mg, 6.20 µmol, 1 eq), TEA (4.2 µL, 30 µmol, 4 eq) were dissolved in 300 µL DMSO in a sample vial. The reaction was stirred overnight at room temperature shielded from light. The crude was loaded on a Biotage Isolera One column machine (Biotage Sfär 6 g prepacked silica column) with a gradient of $CHCl_3$/MeOH/isopropylamine 100/0/0 to 87.5/10/2.5 v/v. Further purification was done on a C18 reverse phase column using a Biotage Isolera One column machine (Biotage Sfär 6 g prepacked silica C18 column) with a gradient of water/ACN 60/40 to 100/0 v/v. The product fraction was verified with LC-MS and freeze-dried to obtain the product as a blue sticky solid (1.8 mg, 14%).

LC-MS: Rt = 5.05 min, calculated m/z 2125.40, calculated m/z internal ester 2108.40, observed m/z = 1069.42 [internal ester + MeOH + H]$^{2+}$, 705.92 [internal ester + 2H]$^{3+}$, 529.75 [internal ester + 3H]$^{4+}$

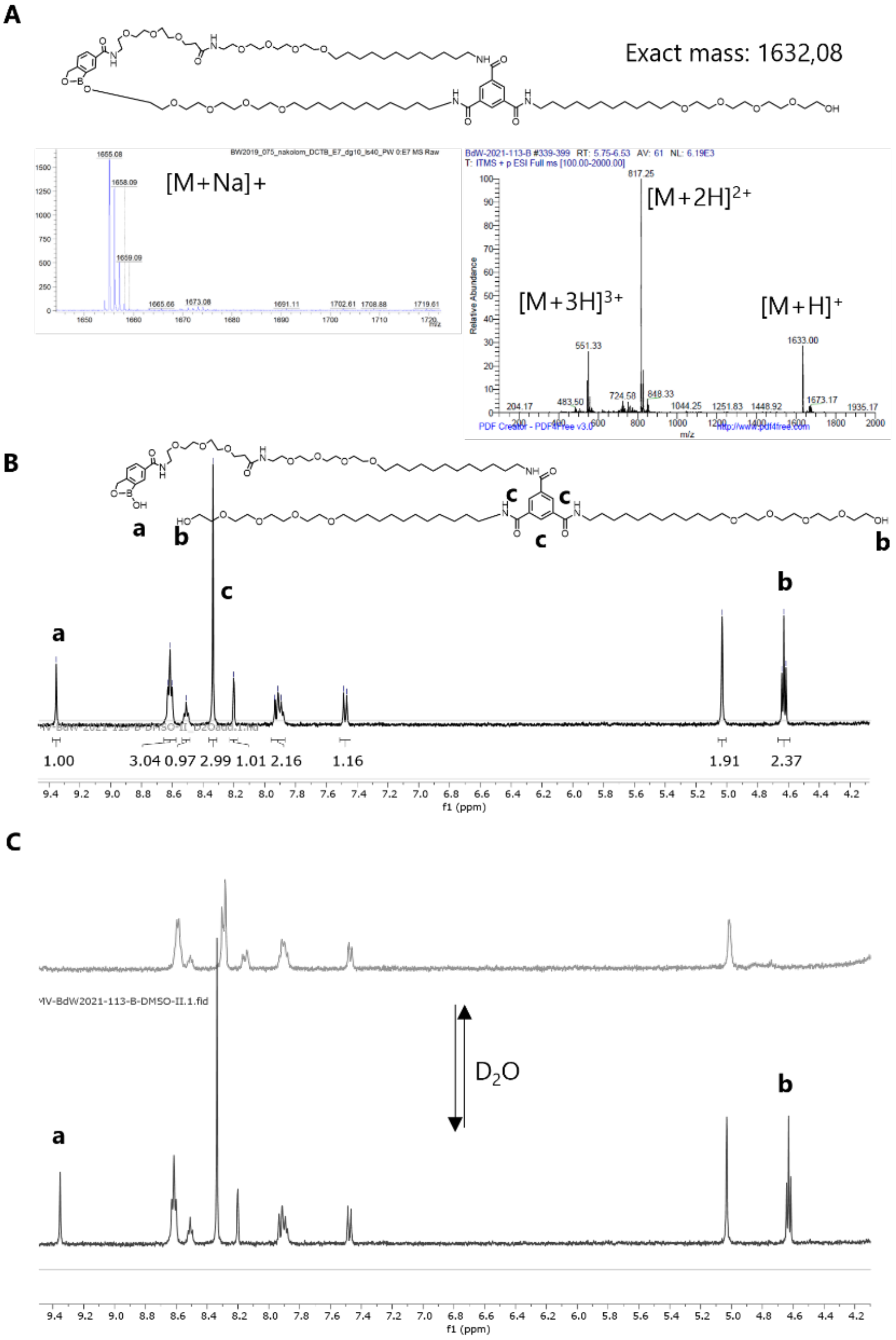

Figure S5. (A) Boronic ester derivative formation during analysis of BTA-Ba, with corresponding mass spectra from MALDI-ToF and LC-MS. (B) ^{1}H NMR spectrum of BTA-Ba in DMSO-d_6. (C) ^{1}H NMR spectra of BTA-Ba in DMSO-d_6 (black, bottom) with a drop of D_2O (grey, top) wherein the peripheral hydrogens exchange for deuterium and the signals of a and b disappear.

Additional note on the internal ester formation for benzoxaborole functionalized BTAs: During the synthesis of BTA derivatives presenting benzoxaborole, mass analysis (MALDI-ToF as well as LC-MS) indicates the presence of boronic ester derivatives, wherein the peripheral alcohol on the Ba and the BTA side chains undergo an intramolecular reaction (Figure S4A). The presence of the intramolecular BTA boronic ester derivate is undesirable, as the boronic acid would not be available for binding with sialic acid. Therefore, an ^{1}H NMR (in DMSO-d_6) was measured to rule out this boronic ester formation. Figure S4B shows the spectrum of the BTA-Ba product wherein the signals of both alcohols integrate correctly. A small-scale proton-deuterium exchange (HDX) experiment was performed by adding a drop of D_2O to the sample in DMSO-d_6. The most labile protons were exchanged for a deuterium, meaning that these proton peaks disappeared in the spectrum (Figure S4C). Hence, these protons were present in the sample and the product was formed. The formation of the boronic ester derivate occurs during ionization, or due to the slightly acidic environment during LC-MS due to the presence of formic acid in the mobile phase.

Sample preparation:
BTA solution: Stock solution of solid BTA and desired functional BTA derivative with ligand (BTA-Ba, BTA-Bio, BTA-DNA, etc.) were weighed and mixed with MQ water to prepare stock solutions. In a closed capped glass vial, required volume of BTA stocks were mixed to yield the desired molar ratio and overall concentration was adjusted with MQ water. The mixture was stirred at 80 °C for 15 minutes. The mixture was stirred at 80 °C for 15 minutes. The resulting hot mixture was vortexed for 15 seconds and allowed to equilibrate at room temperature overnight. The samples were stored in fridge for up to 1 week.

BTA samples with fluorescent dyes: Stock solution of solid BTA and functional BTA derivatives with ligands (BTABa, BTABio, BTADNA, etc.) were weighed and mixed with MQ water to prepare stock solutions. In a closed capped glass vial, required volume of BTA stocks were mixed to yield the desired molar ratio and overall concentration was adjusted to 50 μM with MQ water. The mixture was stirred at 80 °C for 15 minutes. The resulting hot mixture was vortexed for 15 seconds. To it, fluorescent BTA derivative with dye (BTA-Cy3, BTA-Cy5, BTA-Ba-Cy3, BTA-Ba-Cy5, etc.) was added from their Methanol stock solution. The solution was stirred at 45 °C for 15 minutes and subsequently allowed to equilibrate at room temperature overnight. The samples were stored in fridge for upto 1 week.

UPy samples with fluorescent dyes: Stock solution of solid UPy and functional UPy derivatives with ligands (UPyBio, UpyCy3) were weighed and mixed in Methanol to prepare stock solutions. In a closed capped glass vial, required volume of UPy stocks were mixed to yield the desired molar ratio and overall concentration was adjusted to 50 μM with MQ water. The solution allowed to equilibrate at room temperature overnight. The samples were stored in fridge for up to 1 week.
Small Unilamellar Vesicles (SUVs) preparation: Stock solution of lipids (0.1-1 mg/mL) were prepared by dissolving required amount of lipids in chloroform. In a glass vial, the estimated volume of lipid stocks were mixed in the desired molar ratio. Chloroform from solution was evaporated at room temperature under a nitrogen stream to obtain lipid films. The vial was vacuum dried for 1 hour at room temperature. The dried lipid film was then resuspended in estimated volume of MQ water to attain lipid vesicles with total lipid concentration of 1 mg/mL. This solution was sonicated for 5 minutes and extruded through 0.1 μm membrane filter at least 11 times to obtain SUVs with size ca. 100 nm. The SUVs were stored in refrigerator at 4 °C and used up to 10 days.
*In case of DPPC based SUVs, the solution was heated at 60 °C (above its transition temperature) for 1 hour prior to extrusion.

Supported lipid bilayer (SLB) preparation: SLBs were prepared in well plates.
Well plates were cleaned by immersing and sonicating in solution of (i) 1 % w/v sodium dodecyl sulfate (SDS) solution, (ii) 70 % v/v ethanol solution and (iii) MQ water for 10 minutes each. After each step, the well-plates were thoroughly rinsed with MQ water. The well plates were then dried under a nitrogen stream and activated by UV/ozone treatment for 10 minutes.
The well plates surface was then made hydrophilic for vesicle fusion by 2M NaOH solution. To the wells, 200 μL 2M NaOH was added, and the well plate was incubated for 1 hour at room temperature. The wells were then thoroughly rinsed with MQ water at least 3 times to remove excess NaOH.
SUVs solution with concentration of 1 mg/mL in MQ water was diluted to 0.1 mg/mL in 1×PBS and added to the wells. The well plate was incubated for 30 minutes for vesicle fusion and SLB formation. Thereafter, wells were carefully washed with 1×PBS at least 3 times to remove excess SUVs from the wells. The SLB now should be carefully handled with no bubbles or drying to avoid SLB disruption and kept under 1×PBS.
For SAv appended SLB, the SLB were injected with 200 μL 1 μM SAv solution in 1×PBS and incubated for 5 minutes. Thereafter, wells were carefully washed with 1×PBS at least 3 times to remove excess SAv from the wells.
For DNA based receptor, the SAv appended SLB is injected with 200 μL 1 μM DNA_nBio solution in 1×PBS and incubated for 5 minutes. Thereafter, wells were carefully washed with 1×PBS atleast 3 times to remove excess DNA_nBio from the wells.

Anchoring of supramolecular polymers on SLB:
Solution of fibers were diluted from 50 μM in MQ to 2.5 μM 1×PBS prior to imaging.
This solution was added to wells and incubated for desired time (1 hour in case of BTA-Ba and 1 minute in other cases).

STORM samples:
Imaging on glass. 50 μM BTA samples in Milli-Q water were diluted to 1 μM in 1×PBS and flown in an imaging chamber (see Instrumentation). After annealing for 1 minute, the chamber was washed with 1×PBS and STORM buffer (50 mM 2-aminoethanol, 3% (v/v) OxyFluor, 20% (v/v) sodium DL-lactate, pH adjusted to 8-8.5 with NaOH).

Imaging on SLB. 50 μM samples of BTA fibers in Milli-Q water were diluted to 1 μM in 1×PBS. Then, 200 μL BTA solution was added to the SLB. BTAs were incubated on the SLB for one hour after which unbound BTAs were removed from the well by washing three times with 1×PBS. Finally, the well was washed with STORM buffer (see above).
The well-plates were washed twice with 1×PBS and then 200 μL STORM buffer was added. STORM buffer contains 50 mM Tris pH 7, an oxygen scavenging system (0.5 mg/mL glucose oxidase, 40 μg/mL catalase), 10% (w/v) glucose and 10 mM 2-aminoethanethiol.
For BTA-Ba, BTA-Ba-Cy3, and BTA-Ba-Cy5, we used the other buffer without glucose to prevent unbinding of the BTAs. STORM buffer: 50 mM 2-aminoethanol, 3% (v/v) OxyFluor, 20% (v/v) sodium DL-lactate, pH adjusted to 8-8.5 with NaOH.

Protocol for dynamic monomer exchange:
SLB is prepared as discussed above, to it 200 μL of 2.5 μM desired BTA sample solution (with ligand and dye 1) is added and incubated for 5 minutes. The well is washed 1×PBS solution three times to remove excess fibers. To it 200 μL of appropriate ratio of BTA sample (with dye 2) is added and images at time intervals are recorded.

Protocol QCM-D measurements:
The measurements were performed on activated SiO_2-coated sensors at 22 °C. A flow rate of 50 μL/min were set. For analysis, the fifth overtone was used for the change in frequency (Δf_5) and dissipation (ΔD_5). All solutions were prepared in 1×PBS and prior to measurements 10 mM PBS was used to obtain a stable baseline. After each step 1×PBS is injected for washing.
SiO_2-coated sensors for QCM-D: Sensors were cleaned by immersing them for 30 minutes in (i) 2 % w/v SDS solution, * and(ii) MQ water (after thorough rinsing with MQ). The sensor surfaces should be kept wet after SDS treatment until thoroughly washed. The sensors were then dried under a nitrogen stream and activated by UV/ozone treatment for 10 minutes.
Preparation of SLB: The 0.1 mg/mL solution of SUVs in 1×PBS was flown through the chamber till a stable saturated profile was observed. A typical vesicle adhesion and rupture for SLB formation was seen.
Washing off excess SUVs/lipids: The chambers/sensor was washed with 1×PBS solution till a stable line was observed.
Preparation of SAv-appended SLB: 1 μM solution of SAv in 1×PBS was flown till a stable plateau reached. A typical saturation profile is seen.

Mechanism of anchoring (BTABio / UPyBio and SAv system)

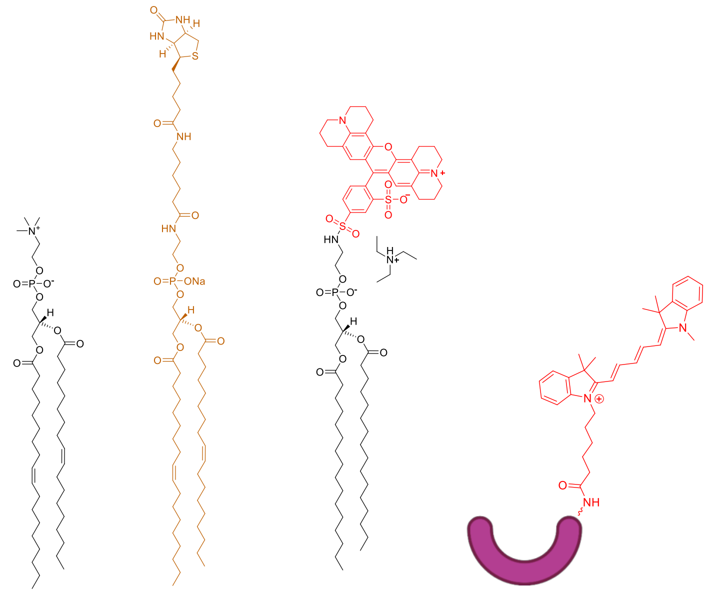

Figure S6. *Structure of DOPC, DOPEbiotin, TR-DHPE and SAvCy5.*

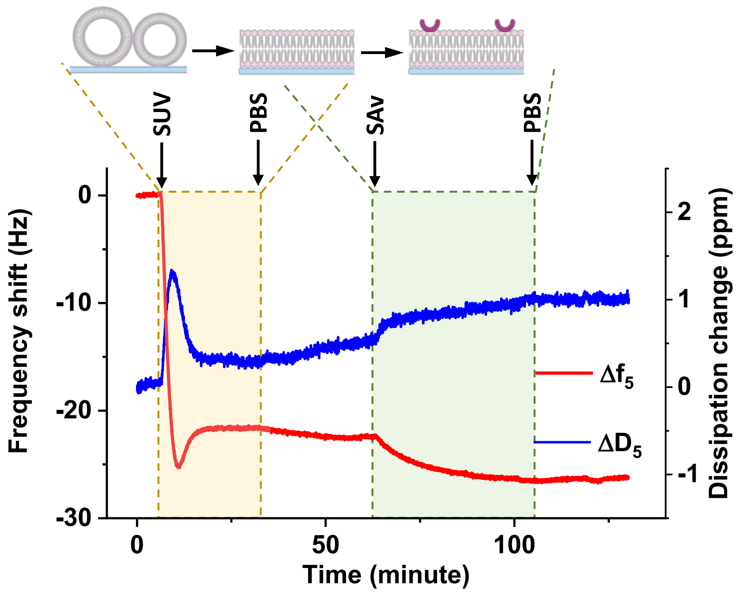

Figure S7. *QCM-D measurement depicting stepwise formation of SAv appended SLB from adsorption of SUVs, vesicle fusion for SLB formation and SAv binding. 0.1 mol% of DOPE–biotin in DOPC. The 5th overtone for frequency shift and change in dissipation is shown. The sections are represented by different colors.* $C_{T,lipids}$ *= 0.1 mg/mL,* $c_{T,SAv}$ *= 1* μ*M.*

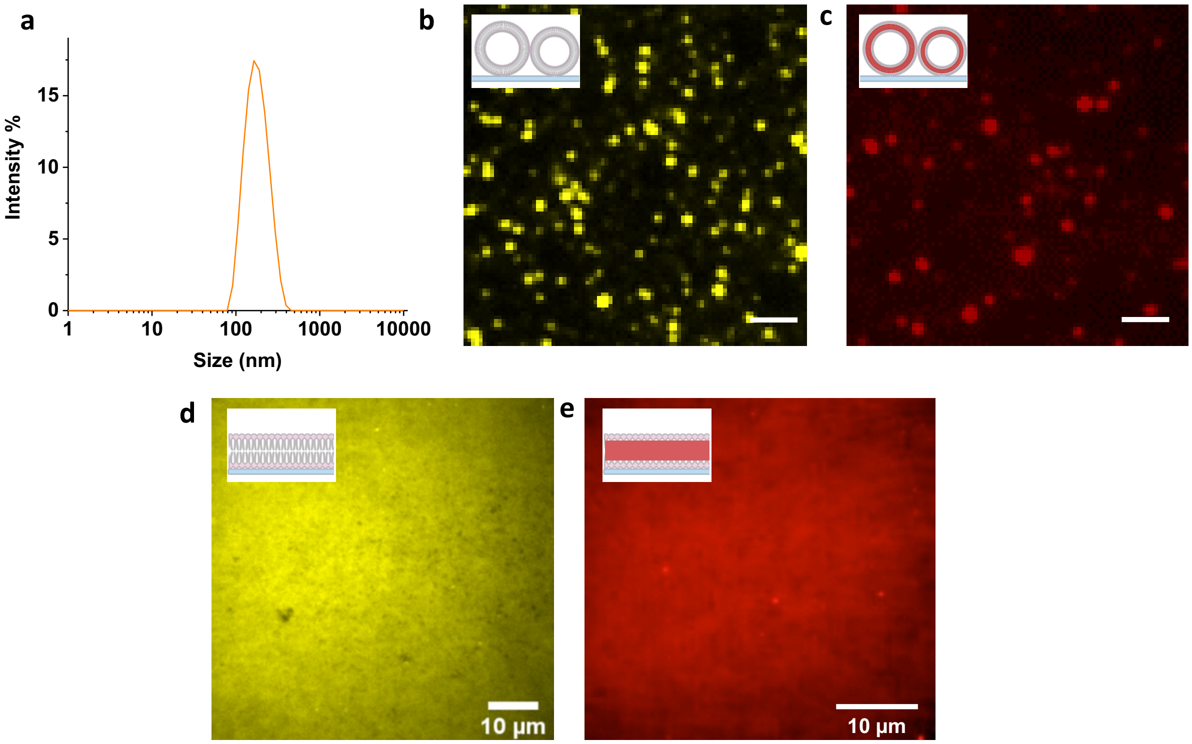

Figure S8. *(a) Dynamic light scattering of SUVs. TIRF image of (b) SUVs with 1% DOPE-TR, (c) SUVs with Nile red, (d) SLB with 1% DOPE-TR and I SLB with Nile red. 0.1 % DOPEBio in DOPC,* $c_{T,lipids}$ *= 0.1 mg/mL,* $c_{T,nile\ red}$ *= 1* μ*M.*

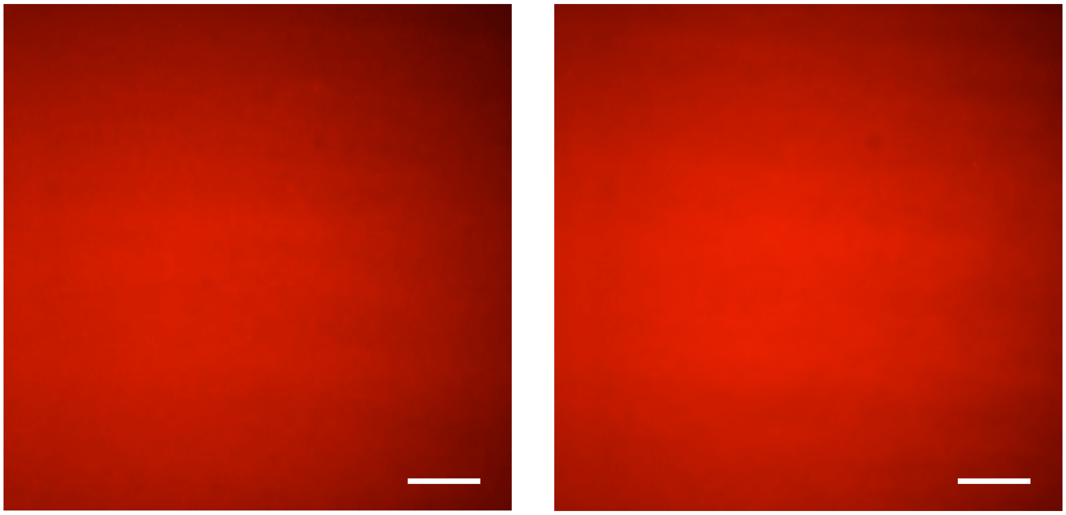

Figure S9. *SLB with SAvCy5 on 0.1 % DOPEBio in DOPC.* $c_{T,SAv}$ *= 1* μ*M.*

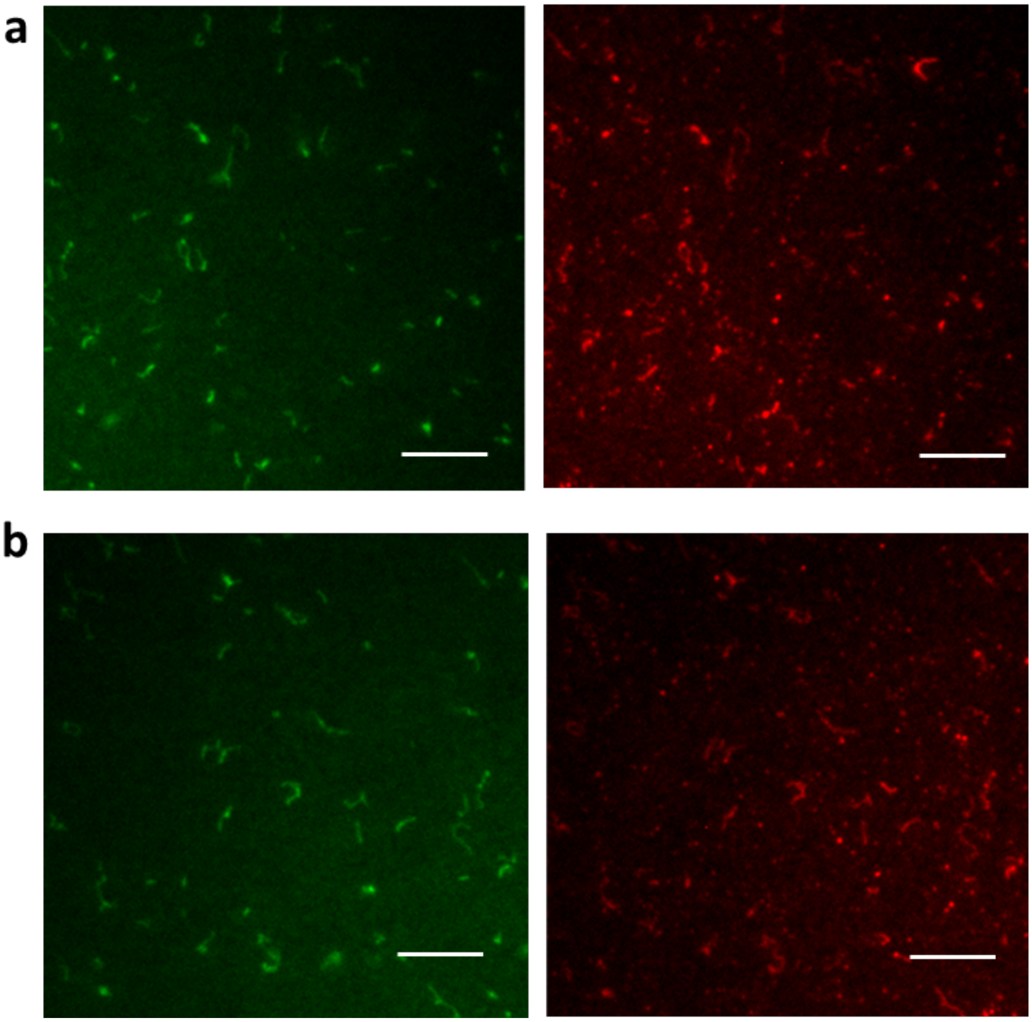

Figure S10. *Two channel TIRF image for BTABioCy3 (1% BTA-Bio and 5% BTA-Cy3) and SAvCy5 respectively depicting (b) anchoring of supramolecular polymers and corresponding receptor recruitment in presence of ligand.* $c_{T,BTA}$ *= 2.5* μ*M, scale bar = 10* μ*m*

a

b

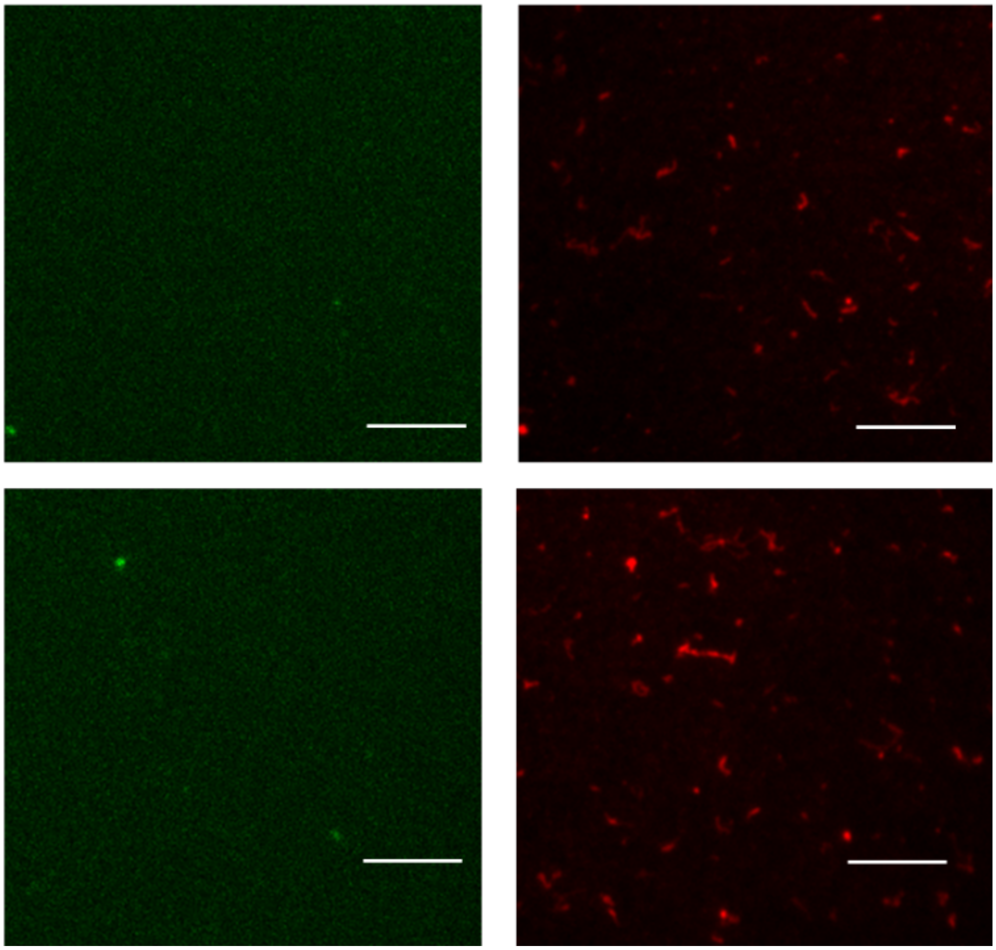

Figure S11. *Two channel TIRF image for BTABio (1% BTA-Bio) and SAvCy5 respectively depicting no visible fibers but recruitment of receptors in absence of dye (BTACy3) and presence of ligands 0.1% DOPE-Bio in DOPc.* $c_{T,BTA}$ *= 2.5* μ*M, scale bar = 10* μ*m*

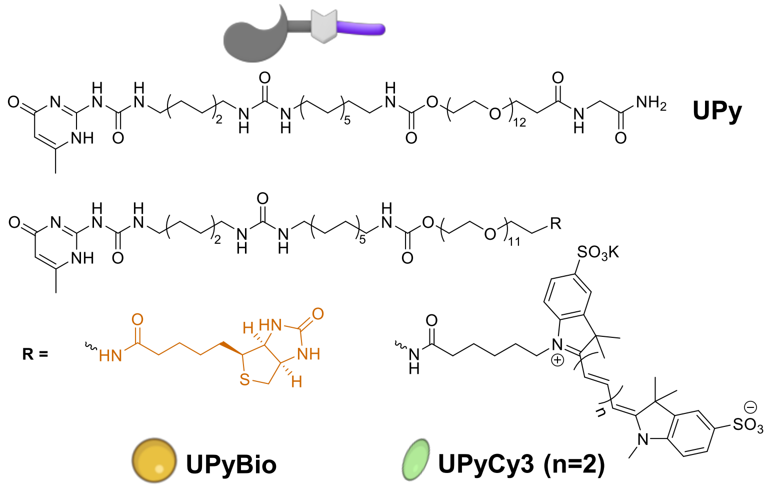

Figure S12. *Structure of UPy derivatives.*

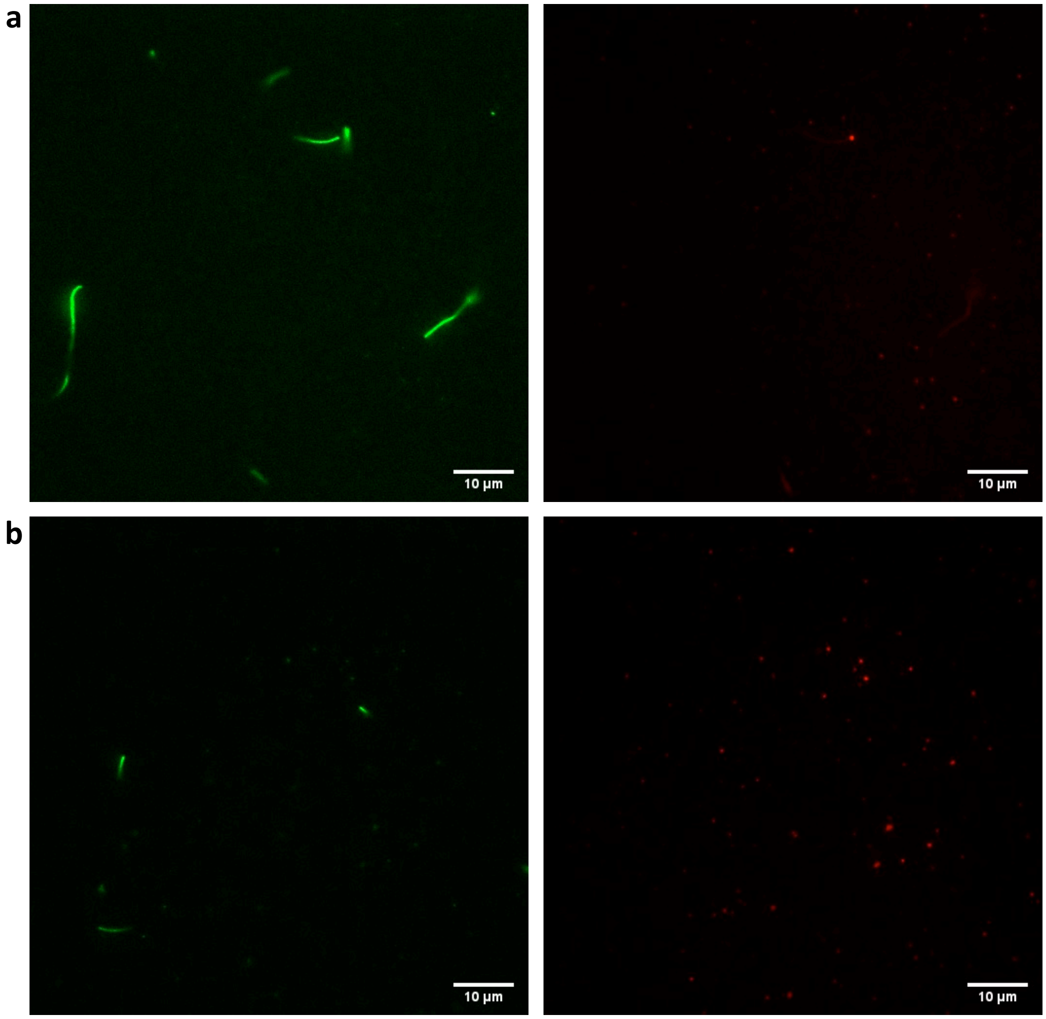

Figure S13. *Two channel TIRF image for (a) UPyBioCy3 and SAvCy5 and (b) UPyCy3 and SAvCy5 respectively depicting no anchoring of fibers and no recruitment of receptors. 0.1 % DOPE-Bio in DOPC (a) [UPyBio] = 1 %, (a,b) [UPyCy3] = 5 %, $c_{T,UPy}$ = 10 μM, scale bar = 10 μm*

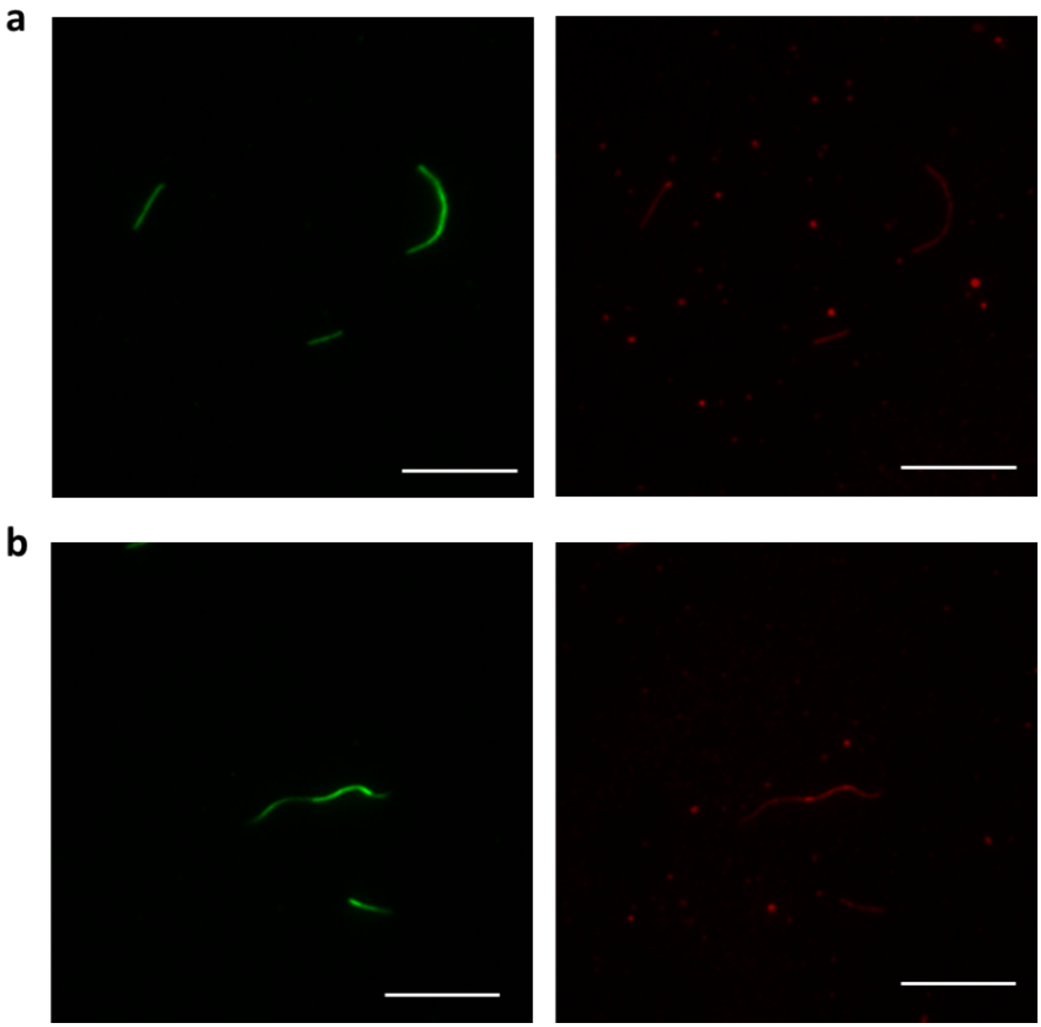

Figure S14. *Two channel TIRF image for (a) UPyBioCy3 and SAvCy5 and (b) UPyCy3 and SAvCy5 respectively depicting anchoring of fibers and recruitment of receptors after 30 minutes. (a) [UPyBio] = 1 %, (a,b) [UPyCy3] = 5 %,* $c_{T,UPy}$ *= 10* μ*M, scale bar = 10* μ*m*

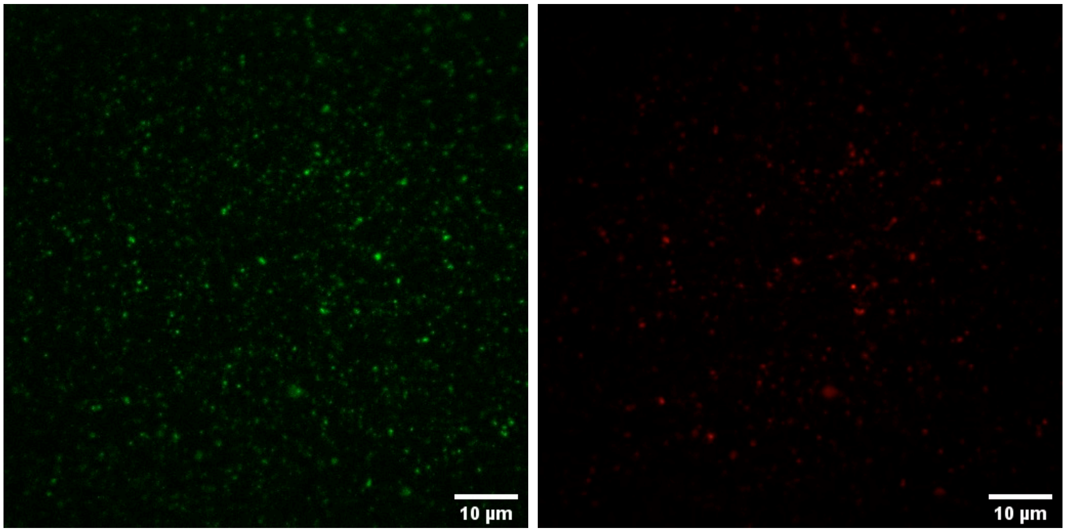

Figure S15. *Two channel TIRF image for dynamic supramolecular polymer BTABioCy3 (1% BTA-Bio and 5% BTA-Cy3) and receptor SAvCy5 on immobile SLB composed of 0.1 % DPPE-Bio in DPPC depicting no anchoring of supramolecular polymers or receptors recruitment.* $c_{T,BTA}$ *= 2.5 μM, scale bar = 10 μm.*

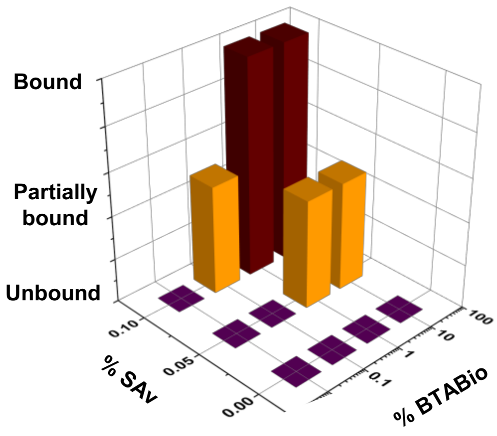

Figure S16. *Effect of change in % ligand (BTABio) and % receptor (SAv) on anchoring of supramolecular polymers to SLB.*

Note: Quantitative analysis with high certainty could not be obtained due to uncontrolled number of fibers in field of view.

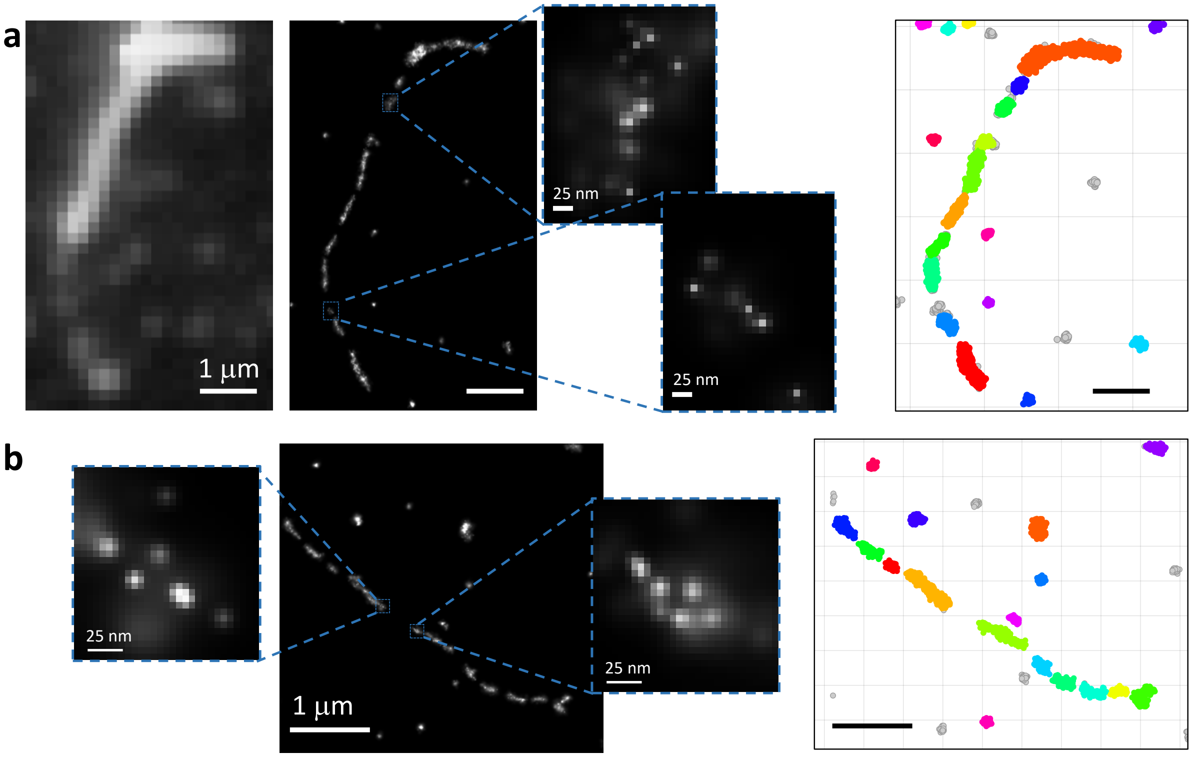

Figure S17: *STORM imaging displaying formation of clusters of receptors labelled with Cy5 after anchoring of supramolecular polymers and corresponding nearest neigh our cluster visualization through MATLAB clustering algorithm.*

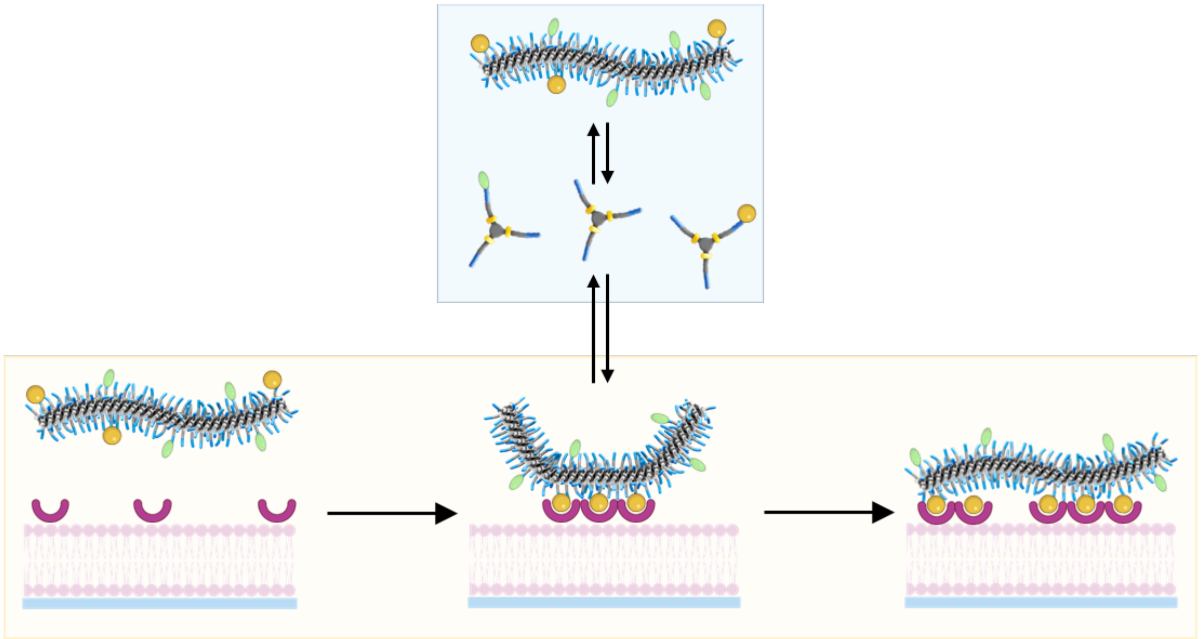

Figure S18. *The enrichment hypothesis refers to enrichment of ligand monomers in anchoring/anchored supramolecular polymers via interfiber monomer exchange.*

Stochastic model

Stochastic model for dynamic recruitment of receptors

To understand the effect of the dynamics of receptors and ligands on binding, we propose a stochastic model. In this model the complex phenomenon of binding is approximated by applying constraints on neighbouring receptors and ligands as we will explain next.

Mathematical model:

In this supplementary material we represent random variables by capital letters (e.g., X), and $X \sim F$ implies that X follows distribution F (e.g., a Gaussian distribution).

Ligands:

We model a single fiber consisting of $n_{\text{fiber}} = 3001$ monomers that have size 3nm by 1/3 nm. The number of monomers that are functionalized with a ligand (N_{lig}) is random and follows a Poisson distribution with mean $n_{\text{fiber}} \cdot C_{\text{lig}}$, i.e.

$$N_{\text{lig}} \sim \text{Poi}\left(n_{\text{fiber}} \cdot C_{\text{lig}}\right),$$

and the ligands are placed uniformly at random on the fiber at positions $\boldsymbol{X}_0$. Over time ligands can exchange and therefore 'move' over the fiber.

The position of ligand i ($\in [1, N_{\text{lig}}]$) at time t is modelled with a discretized Brownian motion as

$$X_{ti} = X_{0i} + \sum_{s=1}^{\frac{t}{\Delta t}} f_r\left(3 \cdot \left(Z_{is}\sqrt{2D_{\text{lig}}\Delta t}\right)\right) \cdot \frac{1}{3},$$

where $f_r(\cdot)$ represents the standard rounding function (e.g., $f_r(1.7) = 2$ and $f_r(1.2) = 1$), $Z_{is} \sim N(0,1)$ (follows a standard Gaussian distribution) and independent for different i,s, Δt equals the time step and D_{lig} equals the diffusion coefficient. The discretization is important when considering clustering as we introduce later. Otherwise, several ligands can cluster on a single monomer with significant probability. The random variable X_{it} thus equals a one-dimensional random walk over the fiber (whose monomers have width 1/3 nm). As a boundary restriction we let $X_{ti} = X_{t-1,i}$ if X_{ti} would be realized outside of the fiber.

Receptors:

The ligands may interact with receptors on a lipid membrane of dimension 1µm x 1µm that consists of $n_{\text{membrane}} = 2000$ by n_{membrane} monomers (with a diameter of 1nm). A monomer on the membrane is thus three times as wide as the units on the fiber. The total number receptors (N_{lip}) follows a Poisson distribution with mean $n_{\text{membrane}} \cdot n_{\text{membrane}} \cdot C_{\text{lip}}$, i.e.,

$$N_{\text{lip}} \sim \text{Poi}\left(n_{\text{membrane}} \cdot n_{\text{membrane}} \cdot C_{\text{lip}}\right).$$

A receptor j ($\in [1, N_{\text{lip}}]$) is placed uniformly at random the membrane at position $\boldsymbol{Y}_{0j} = (Y_{x0j}, Y_{y0j})$. The receptors undergo a two-dimensional Brownian motion with diffusion coefficient D_{lip},

$$\boldsymbol{Y}_{tj} = \begin{pmatrix} Y_{x0j} \\ Y_{y0j} \end{pmatrix} + \sum_{s=1}^{\frac{t}{\Delta t}} \begin{pmatrix} H_{xjs}\sqrt{2D_{\text{lip}}\Delta t} \\ H_{yjs}\sqrt{2D_{\text{lip}}\Delta t} \end{pmatrix},$$

where H_{xjs} and H_{yjs} are independent and standard Gaussian distributed. As for the ligands, if a lipid's position would be realized such that it is outside of the membrane, then $\boldsymbol{Y}_{tj} = \boldsymbol{Y}_{t-1,j}$. Two examples of initial realizations of the ligands and receptors can be found in Fig.S19. No rounding is applied to reduce the computation time of the simulations.

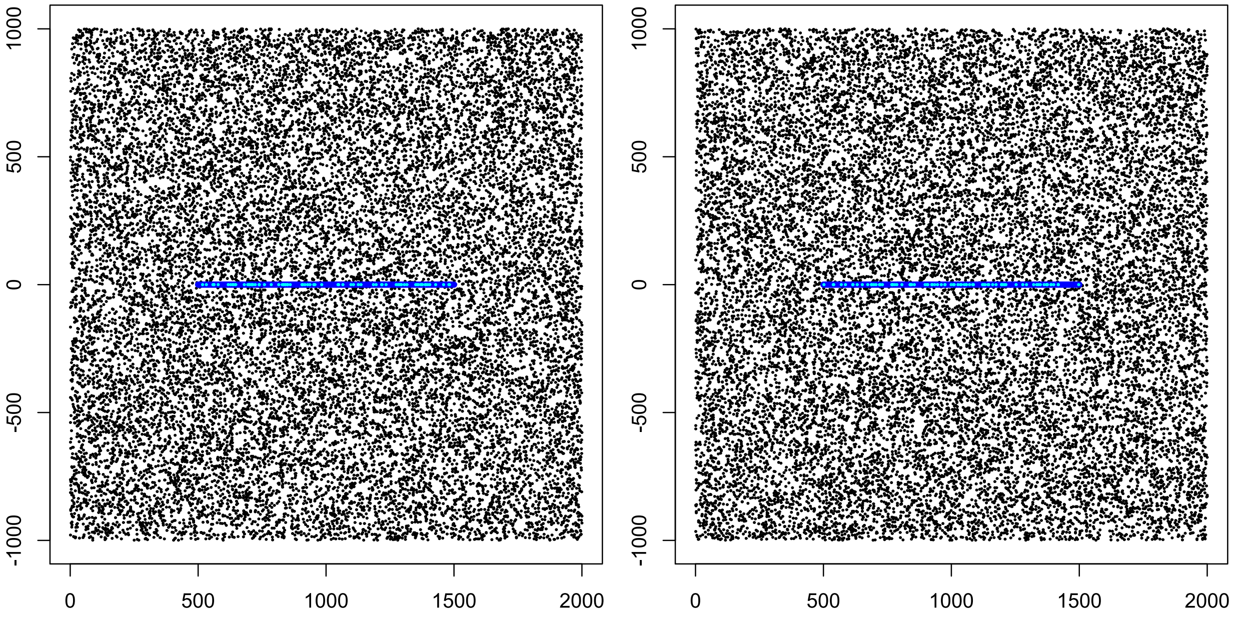

Figure S19. Two examples of realizations of the stochastic model at $t = 0$. Receptors are presented as black dots, the fiber is highlighted in dark blue and the realized ligands are marked with cyan dots. The parameters for these examples were set equal to $C_{lig} = 0.025, C_{lip} = 0.005, n_{\text{membrane}} = 2000\ and\ n_{\text{fiber}} = 3001$.

Binding:
We model the process where ligands and receptors bind according to a *clustering restriction*. In this work we do not model the molecular interaction in detail (see references[1,12]), but replace the complex deterministic process by a stochastic process. Bound ligands and receptors are no longer dynamic, so that (over time) in their neighborhood, larger clusters are formed.

We say that ligands and receptors form a *cluster* when:
within d_{fiber} subsequent units on the fiber at least n_{cluster} ligands are contained,
there are also at least n_{cluster} lipids at a distance of d_{lip} nm (d_{lip} membrane units) of those d_{fiber} units on the fiber.
If so, then an optimal matching (smallest sum of distances between matched receptors and ligands) is applied to pair ligands to receptors. In our main setting we use $d_{\text{fiber}} = 20,\ n_{\text{cluster}} = 5,\ d_{\text{lip}} = 5$, and the binding constraints are graphically presented in Figure 3B.

Dynamics of lipids and ligands:
The average number of bound ligands (equal to the number of bound lipids) over time does depend on the mobility of the ligands and receptors. We have considered three types of dynamics for the lipids and ligands:
diffusing lipids and ligands ($D_{\text{lip}} > 0, D_{\text{lig}} > 0$) with binding,
diffusing lipids and fixed ligands ($D_{\text{lip}} > 0, D_{\text{lig}} = 0$), with binding,
diffusing lipids and ligands without binding ($D_{\text{lip}} > 0, D_{\text{lig}} > 0$).
For the last case, over time, we have tracked how many ligands meet the clustering restriction, but we did not fix any ligands or lipids. The mean number of ligands that meet the clustering restriction for setting 3 is equal to the mean number for the case where the ligands and receptors are both fixed and cannot diffuse ($D_{\text{lip}} = 0, D_{\text{lig}} = 0$).

Simulation results
For varying values of $C_{\text{lig}} \in \{0.01, 0.025, 0.05, 0.075, 0.1, 0.125, 0.150\}$ and $C_{\text{lip}} \in \{0.005, 0.025, 0.05\}$, we have simulated 1000 instances of the mathematical model described before during 1000 time steps ($\Delta t = 1$).

For diffusing lipids and ligands with binding, the mean number of bound ligands over time is presented for the different settings in Figure S20. As a result of the dynamics and binding the average number of bound ligands increase over time. It becomes clear that for lower ligand densities the receptor density is not affecting the curve, while for higher densities the maximum number of bound ligands is reached faster. Moreover, for higher ligand densities it becomes clear that the receptor density strongly affects the final number of bound ligands, see e.g., the settings with $C_{\text{lig}} \in \{0.125, 0.150\}$ and $C_{\text{lip}} \in \{0.005, 0.025\}$. Note that when the lipids and ligands were not motile the average number would be constant over time.

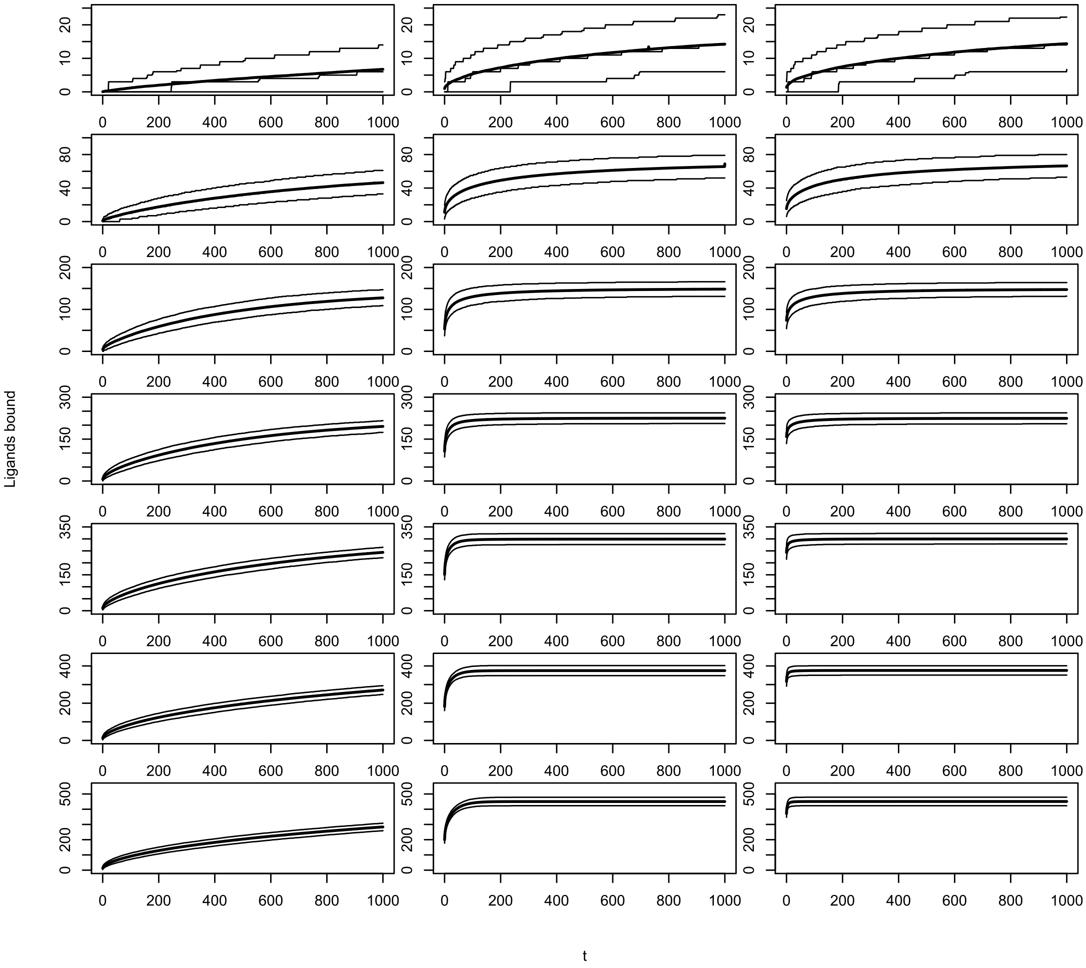

Figure S20. Mean (thick lines), as well as the 10%, 50% and 90% quantiles (thin lines) of the number of bound ligands in 1000 simulations per setting ($d_{\text{fiber}} = 20, n_{\text{cluster}} = 5, d_{\text{lip}} = 5, n_{\text{membrane}} = 2000, n_{\text{fiber}} = 3001, \Delta t = 1$). The rows correspond to the ligand density $(0.01, 0.025, 0.05, 0.075, 0.1, 0.125, 0.150)$ and the columns to the receptor density $(0.005, 0.025, 0.05)$.

To compare the impact of the different types of dynamics we present the C_{lig} versus the mean number of bound ligands at time 100, 500 and 1000 for $C_{\text{lip}} = 0.005$ in Figure S22. Without binding there are (on average) barely any clusters formed at each timepoint. If the receptors move and can bind (so that they are fixed) while the ligands are fixed, clusters are formed. Finally, when the ligands also move the number of bound ligands is even higher. The relation between the ligand density and the mean number of bound ligands is non-linear.

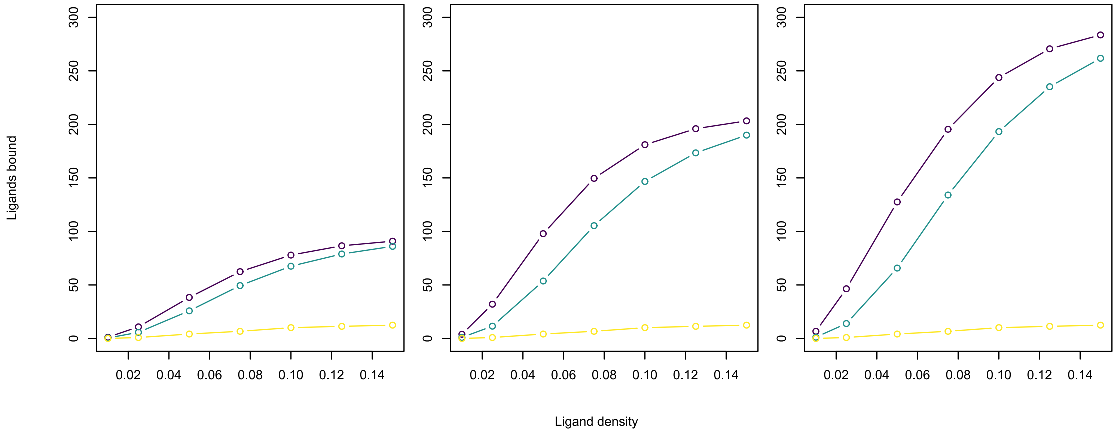

Figure S21. Ligand density versus the mean number of bound ligands from 1000 simulations ($C_{lip} = 0.005$, $d_{\text{fiber}} = 20, n_{\text{cluster}} = 5, d_{\text{lip}} = 5, n_{\text{membrane}} = 2000, n_{\text{fiber}} = 3001, \Delta t = 1$) under different types of dynamics: lipids and ligands diffusing with binding (purple, $D_{lig} = 0.1$), lipids diffusing with binding (green) and without binding (yellow), where at time 100 (left), 500 (middle) and 1000 (right). For the three different types of dynamics the mean number of bound ligands at $t = 500$ are presented for all combinations of C_{lip} and C_{lig} in Figure 3Bi-iii and Figure S22.

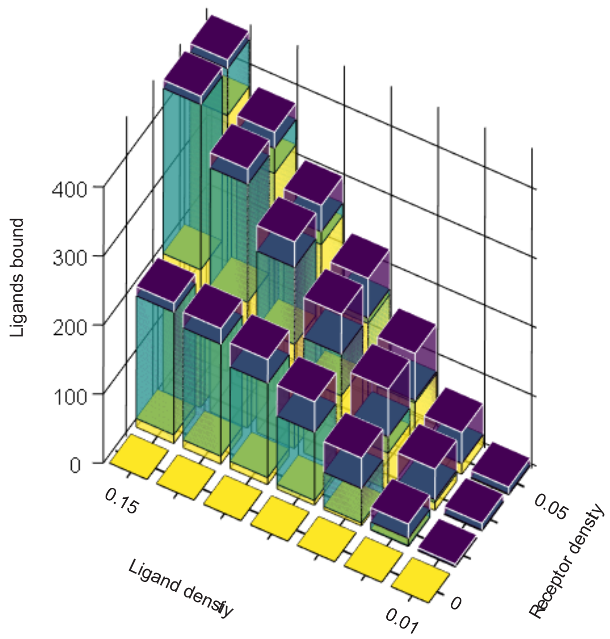

Figure S22. Ligand density and receptor density versus the mean number of bound ligands at $t = 500$ based on 1000 simulations for the different types of dynamics: lipids and ligands diffusing with binding (purple), lipids diffusing with binding (blue) and without binding (yellow).

Effect of the clustering parameters

When we would adjust the clustering parameters, $n_{\text{cluster}}, d_{\text{fiber}}, d_{\text{lip}}$, the level of binding is directly affected. If we increase the number of ligands/lipids that should meet the clustering restrictions from 3 to 5, the mean number of bound ligands drastically decrease as shown in Figure S23. Now, the ligand density should be much higher to observe any clusters at all for the process where both ligands and lipids are diffusing. If the ligands are not diffusing, then an even higher density is necessary to observe any clusters at all.

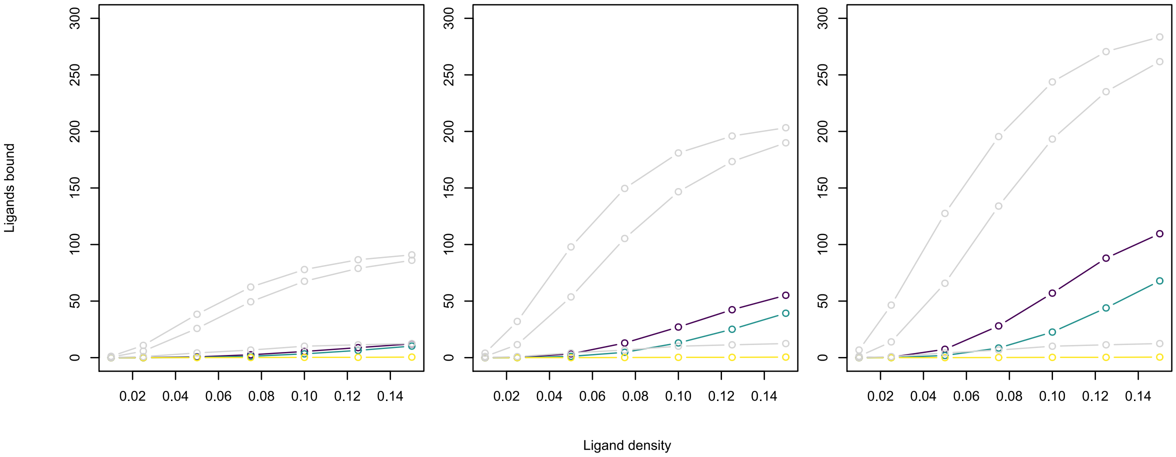

Figure S23. Ligand density versus the mean number of bound ligands from 1000 simulations for $n_{\text{cluster}} = 5$ under different types of dynamics: lipids and ligands diffusing with binding (purple), lipids diffusing with binding (green) and without binding (yellow), where $C_{lip} = 0.005$ at time 100 (left), 500 (middle) and 1000 (right). The lines from Figure S21 are presented in light grey for comparison. Similarly, if we decrease d_{fiber} from 20 to 10, less ligands will meet the restriction, so that the mean number of bound ligands will decrease as shown in Figure S24 However, it is clear that this change has less impact that changing n_{cluster} from 3 to 5 as shown before. We could lower the d_{fiber} further to obtain similar decreases.

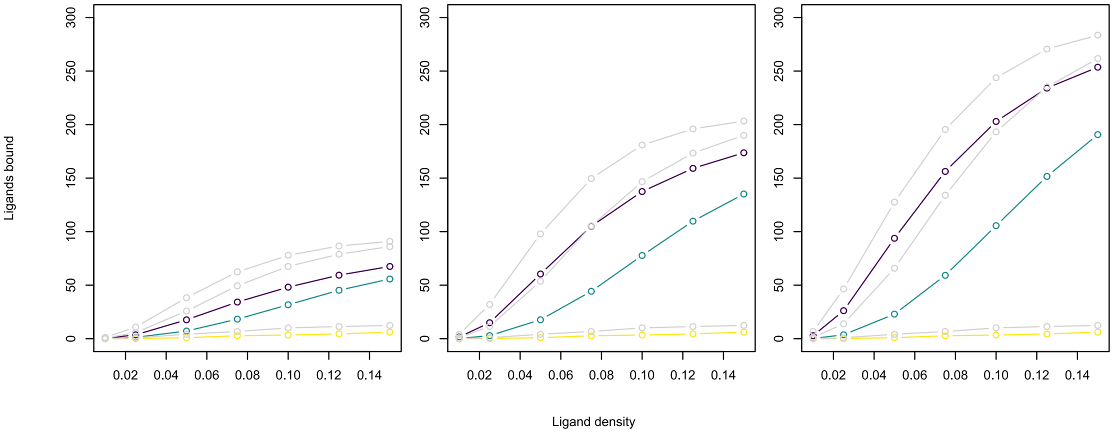

Figure S24. Ligand density versus the mean number of bound ligands from 1000 simulations for $d_{fiber} = 10$ under different types of dynamics: lipids and ligands diffusing with binding (purple), lipids diffusing with binding (green) and without binding (yellow), where $C_{lip} = 0.005$ at time 100 (left), 500 (middle) and 1000 (right). The lines from Figure S21 are presented in light grey for comparison.

Finally, we have changed d_{lip} from 5 to 3, so that fewer lipids would be meet the clustering restriction. Again, the numbers of bound ligands decrease as presented in Figure S25.

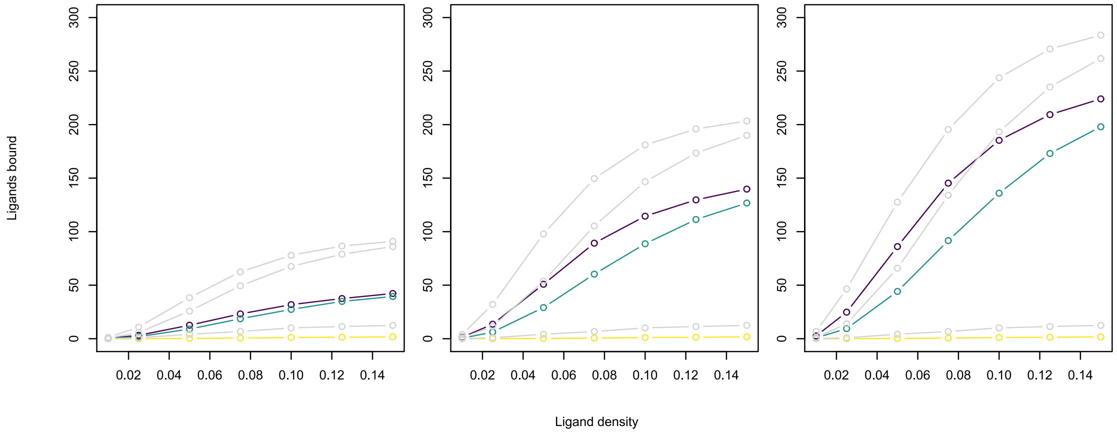

Figure S25. Ligand density versus the mean number of bound ligands from 1000 simulations for $d_{lip} = 3$ under different types of dynamics: lipids and ligands diffusing with binding (purple), lipids diffusing with binding (green) and without binding (yellow), where $C_{lip} = 0.005$ at time 100 (left), 500 (middle) and 1000 (right). The lines from Figure S22 are presented in light grey for comparison.

Sensitivity analysis
In our simulation binding can take place at every time step ($\Delta t = 1$). When we decrease the time step, binding can take place at more moments, but the computation time of the simulation increases. To verify whether our time resolution was high enough we have also run the simulation for $\Delta t = 0.1$, where a very similar curve was observed, as shown in Figure S26.

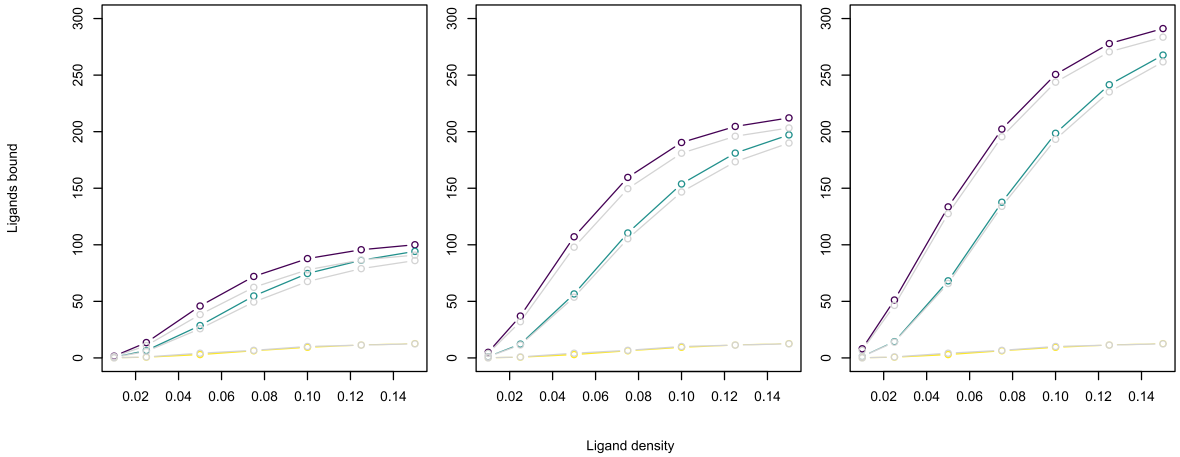

Figure S26. Ligand density versus the mean number of bound ligands from 1000 simulations with $\Delta t = 0.1$ under different types of dynamics: lipids and ligands diffusing with binding (purple), lipids diffusing with binding (green) and without binding (yellow), where $C_{lip} = 0.005$ at time 100 (left), 500 (middle) and 1000 (right). The lines from Figure S22 ($\Delta t = 1$) are presented in light grey for comparison.

Also, when we increase the membrane size, the diffusion of many more receptors should be computed and thus the computing time increases. However, when increasing the membrane width from 2000 to 5000 units the mean number of bound ligands does not change as shown in Figure S27.

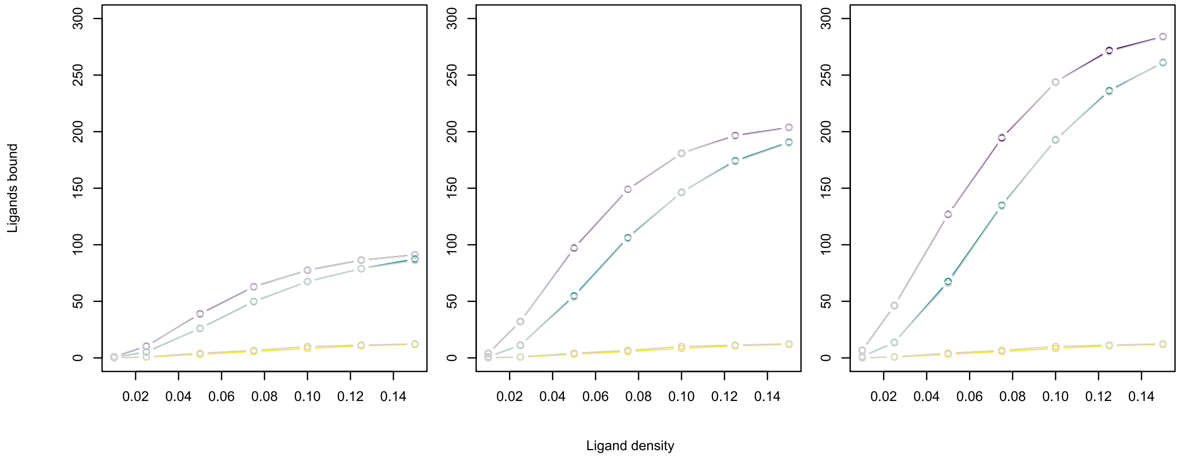

Figure S27. Ligand density versus the mean number of bound ligands from 1000 simulations with $n_{\text{membrane}} = 5000$ under different types of dynamics: lipids and ligands diffusing with binding (purple), lipids diffusing with binding (green) and without binding (yellow), where $C_{lip} = 0.005$ at time 100 (left), 500 (middle) and 1000 (right). The lines completely overlap with those of Figure S22 $(n_{\text{membrane}} = 2000)$.

Molecular models and molecular dynamics simulations:

BTA-fiber model:

The minimalistic, implicit-solvent BTA coarse-grained (CG) model employed herein is has been adapted from our previous works.[13,14] In particular, the model is representative of threefold symmetric monomers having three side amphiphilic arms (with a hydrophobic interior and hydrophilic terminal units) surrounding a planar core. that interact directionally with each other consistently with how BTA water-soluble monomers interact in water.[17,18] In this model, each BTA monomer is composed of 18 CG particles. At the centre of the molecule there is a CG-bead that containing a rigid central dipole, composed of two small charged beads with a partial charge of $q = \pm 1.4$. The dipole parameters have been tuned to reproduce the same directional core-core interactions seen in atomistic and finer CG BTA models.[18,15,16] Three arms, each composed of 5 CG particles, originate from the central bead: the first 2 CG particles connected to the core are solvophobic, while the three terminal CG particles of each arm are hydrophilic (similar to the C12-TEG side chains of water soluble BTA monomers).[17-20] The intra-molecule bonded interactions are modelled via harmonic bond and angle potentials, the parameters are reported in Ref 18. The non-bond interaction terms between the CG particles constituting the core and arms of the monomers are defined by a via Lennard–Jones (LJ) potential. Complete force field parameters of these models are available at: https://doi.org/10.5281/zenodo.6453179.[17] An initial fibre model composed of 40 monomers has been pre-formed and simulated in periodic boundary conditions. Of such 40 monomers, 4 have been kept restrained to mimicking binding of the fiber with receptors present on a surface. In particular, this has been done by allowing the tip CG particle of one of the 3 arms of such 4 bound monomer to move only on the *xy* plane during the simulation, while all other 36 monomers are free to move in unconstrained way (Figure 3C in the main paper: the yellow CG particles identify the bound terminal units of the bound monomers.) Implicit solvent model has been adopted. A longer fibre model, made of 240 monomers with 24 restrained/bound one has been also created and simulated to check the robustness and size independence of the obtained results. Complete details of all molecular models used for the simulations, and of the simulation parameters (input files, etc.) are available at: https://github.com/GMPavanLab/MultiSPbind (this temporary folder will be replaced with a definitive Zenodo archive upon acceptance of the final version of this paper).

Molecular dynamics equilibration:

After initial energy minimization, the short and long fibre models were simulated in periodic boundary conditions for 10 μs, setting the reference temperature at 300 K with a V-rescale thermostat ($\tau_T = 0.1$ ps).[17] The two fibres were separately equilibrated in implicit solvent via Langevin dynamics, accounting for the friction of the solvent and thermal fluctuations. We used the stochastic dynamics (sd) integrator, setting the inverse of the friction constant to $\tau_T = 0.1$ ps. Coulomb and Lennard-Jones were used to model the non-bonded interaction potentials, truncated, and shifted at $r_c = 1.1$ nm. All simulations were carried out in GROMACS 2021 software[18] patched with the PLUMED 2.7.1 plugin.[19]

Metadynamics simulation and free energy decomposition:

We used metadynamics (MetaD) simulations[20] to explore the most favoured configurations in terms of anchoring points displacement and clustering within the bound fibre model.[17,18] Parallel bias metadynamics (PBMetaD) simulations[21] were carried out using GROMACS 2021 software[22] patched with the PLUMED 2.7.1 plugin.[23] We started from the smaller fibre model, containing 4 anchored monomers and other 36 no bound BTA monomers. During the PBMetaD simulations, an external bias is deposited in such a way to explore all possible internal reorganizations of the N=4 BTA anchored monomers within the bound fibres. This is achieved efficiently by simultaneously applying multiple parallel bias potentials on all the relative distances between the 4 grafted CG particles, which are selected as the collective variables (CVs), and by sampling the relative configurations of the 4 grafted ligands simultaneously. The free energy surface (FES) is then computed on the space of the used CVs. Good convergence in the calculation of the FES was obtained through 40 μs of PBMetaD using a HILLS height of 0.02 kcal mol^{-1}, a Gaussian SIGMA of 0.4 nm, and a deposition time of 10 ps. The free energy has been then reweighted[22] and

represented as a function of the coordination number (Cn) between the grafted ligands (see Figure 2D in the main paper). In particular, in Figure 2D in the main paper, a Cn number tending to 0 means negligible spatial coordination between the anchoring monomers (no clustering: all bound monomers are spatially-separated from each other), while a Cn of, e.g., 4-5 means (in a fiber model such as this one containing 4 anchoring monomers) that all bound monomers are gathered together in a single large cluster (Cn_{max}: maximum clustering).
The enthalpic (ΔH) and entropic (ΔS) contributions to the FES (ΔG = ΔH -TΔS) have been computed from the results of the MetaD calculations as recently done for other molecular systems.[23,24,25] In the case of conformational transitions of bound ligands in solution, being in canonical ensemble, ΔH(Cn) reduces to the internal energy ΔU(Cn), which is the sum of kinetic and potential energy. Furthermore, at constant T, the kinetic energy does not depend on Cn, and the internal energy contribution ΔU(Cn) further reduces to the potential energy of the system $\Delta E^P(Cn)$, which can be computed as follows: $\Delta E^P(Cn) = \langle E^P \rangle_{Cn} - \langle E^P \rangle_{ref}$ where $\langle E^P \rangle_{Cn}$ is the local ensemble average of the potential energy computed over the ensemble configurations that are degenerated on Cn, and $\langle E^P \rangle_{ref}$ is the potential energy in a reference Cn. We thus obtained $\langle E^P \rangle_{Cn}$ from:

$$\langle E^P \rangle_{Cn} = \int E^P \, p(E^P|Cn) dE^P \,,$$

where $p(E^P|Cn)$ is the conditional probability to have a specific E^P in Cn. Once computed $\Delta E^P(Cn)$, then, the entropic term, -TΔS(Cn), is obtained as the difference between the free energy and the potential energy term for each Cn value (see Figure 2E).

Information on the conformational entropy of the fibre in the various sampled clustering states has been obtained by calculating the Root Mean Square Displacement (RMSD) of the fibre (see Figure 2F). This RMSD measurement quantifies the conformational variability of BTA fibre configurations associated to the different sampled Cn states, and for this reason it can be considered proportional to the conformational entropy of the bound fibre (i.e., how much the fibre can oscillate/move once bound in the different clustering configurations). The RMSD result shows how increasing the Cn the fibre gains more degree of freedom, since the latter is blocked and engaged in binding the surface in one single point while the rest of the fibre can freely fluctuate better preserving its entropy. To provide an additional validation of the results obtained via MetaD for the small fibre model, we also analysed a longer fibre model (12 anchored monomers out of 120 total monomers). Given the size of this fibre model, efficient sampling of the Cn states (especially for low Cn values) could be achieved even from classical CG-MD simulations. We could thus estimate also for this case the free energy profile ΔG as a function of Cn from 40 μs of unbiased CG-MD simulations (see Figure S28). Specifically, the free energy profile was extracted from the probability density function distribution corresponding to all possible coordination number (Cn) states between the bound ligands visited by the long fibre along the 40 μs CG-MD trajectory. As seen in Figure S28 below, also in this case the FES has similar features as that of Figure 2D in the main paper, showing intermediate Cn configurations as the most favourable states for this fibre when establishing multivalent interactions with a surface of mobile receptors.

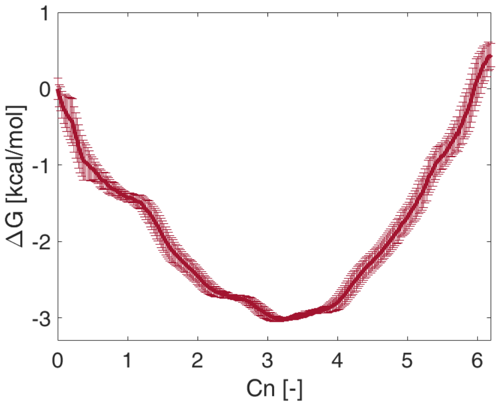

Figure S28. Free Energy profile as a function of the coordination number (Cn) between the 12 anchored monomers (out of 120) obtained from 40μs of unbiased simulation. High Cn indicates bigger clusters.

Effect of association constant and zipping mechanism (BTADNA and DNAnbio; BTABio and SAv)

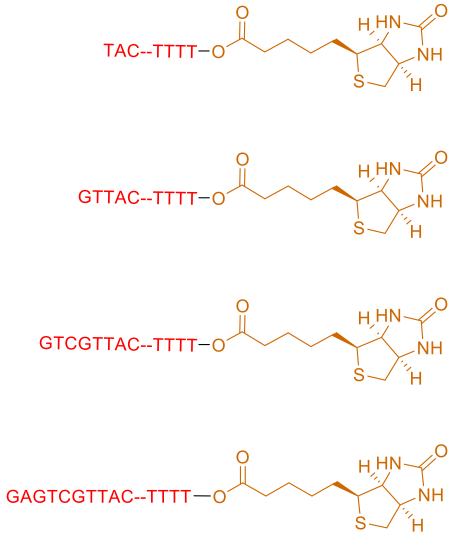

DNA_3bio

DNA_5bio

DNA_8bio

$DNA_{10}bio$

Figure S29. Structures of **DNA_nbio**, n = 3, 5, 8, 10.

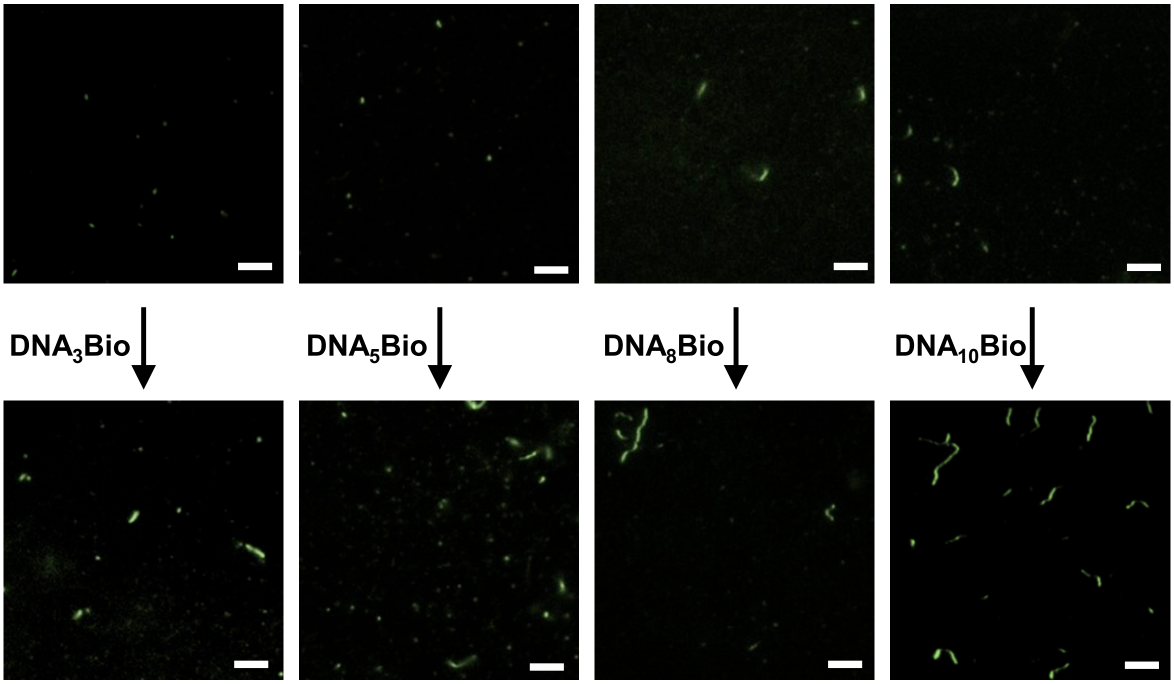

Figure S30. TIRF image of BTA fibers binding in presence of different DNA based receptor (DNAnBio, n = 3-5). An increasing number of bound fibers are observed with increase of n. [Receptor] = 0.1 mol % [BTA-DNA] = 1 %, [BTA-Cy3] = 5%, $c_{T,BTA}$ = 2.5 μM, scale bar = 10 μm

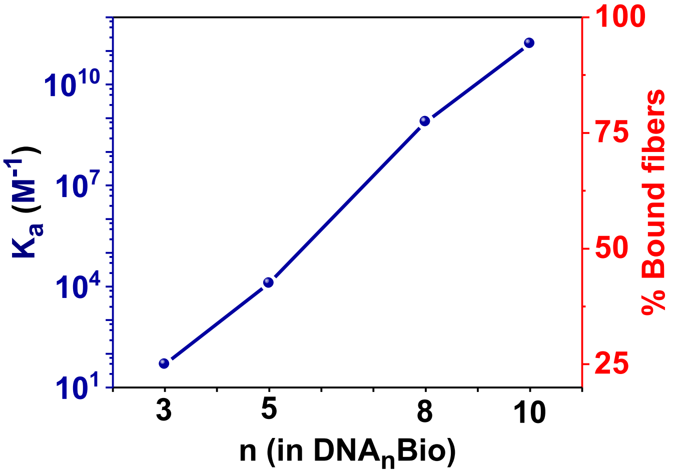

Figure S31. Effect of change in receptor strength on (K_a) on anchoring of supramolecular polymers to SLB. [Receptor] = 0.1 mol % [BTA-DNA] = 1 %, [BTA-Cy3] = 5%, $c_{T,BTA}$ = 2.5 μM, scale bar = 10 μm. This is representative of calculation from 3 samples over 3 field of view each.

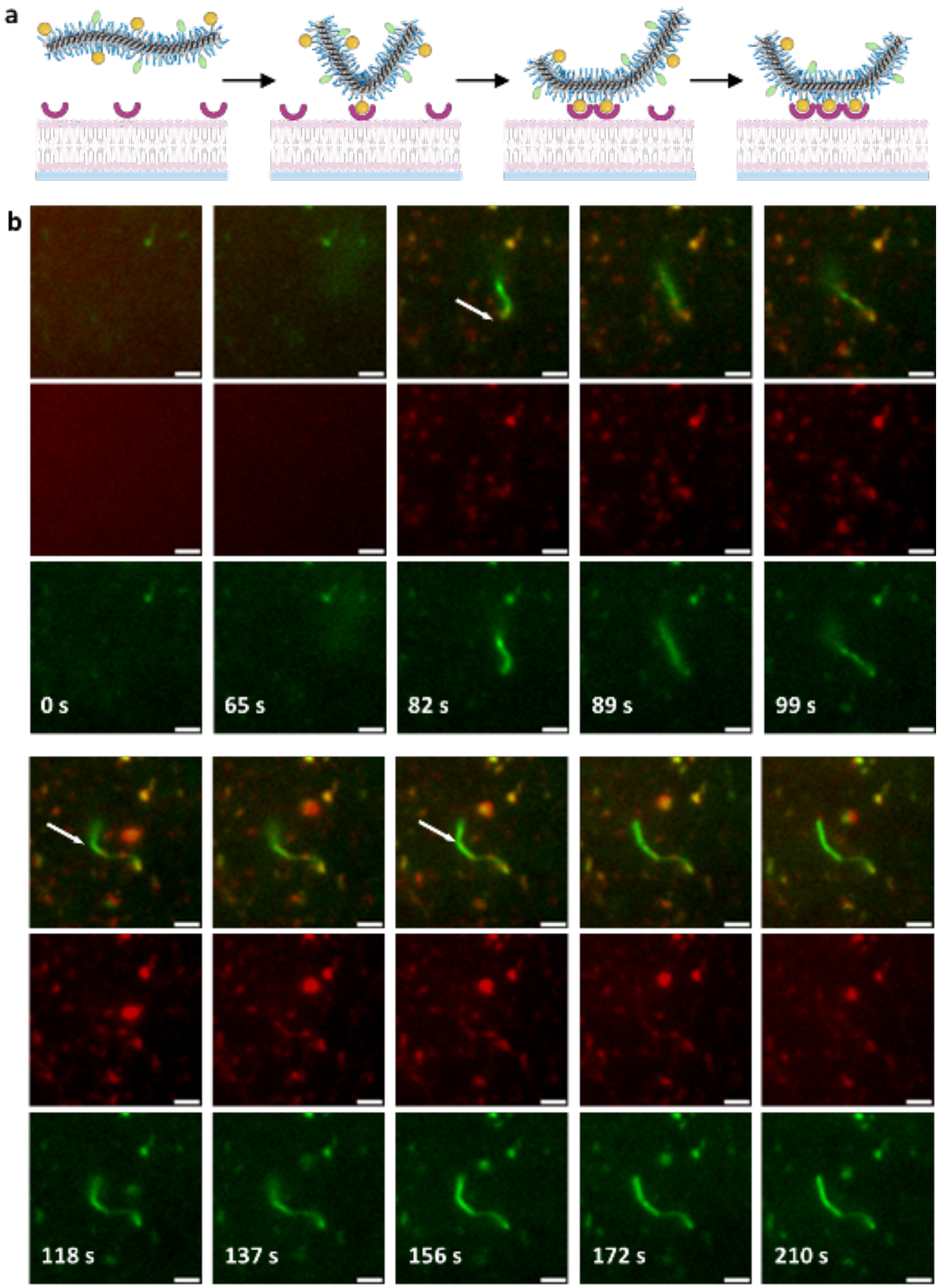

Figure S32. (a) Proposed mechanism of adhesion of fiber to SLB via highly coordinated sequential binding of multiple ligand-receptor pairs. (b) Time lapse imaging showing zipping effect. [Receptor] = 0.1 mol %, [BTA-DNA] = 1 %, [BTA-Cy3] = 5%, DNA_3Biotin = 1 eq., $c_{T,BTA}$ = 2.5 μM, scale bar = 2 μm. Red channel = receptor, Green = BTACy3

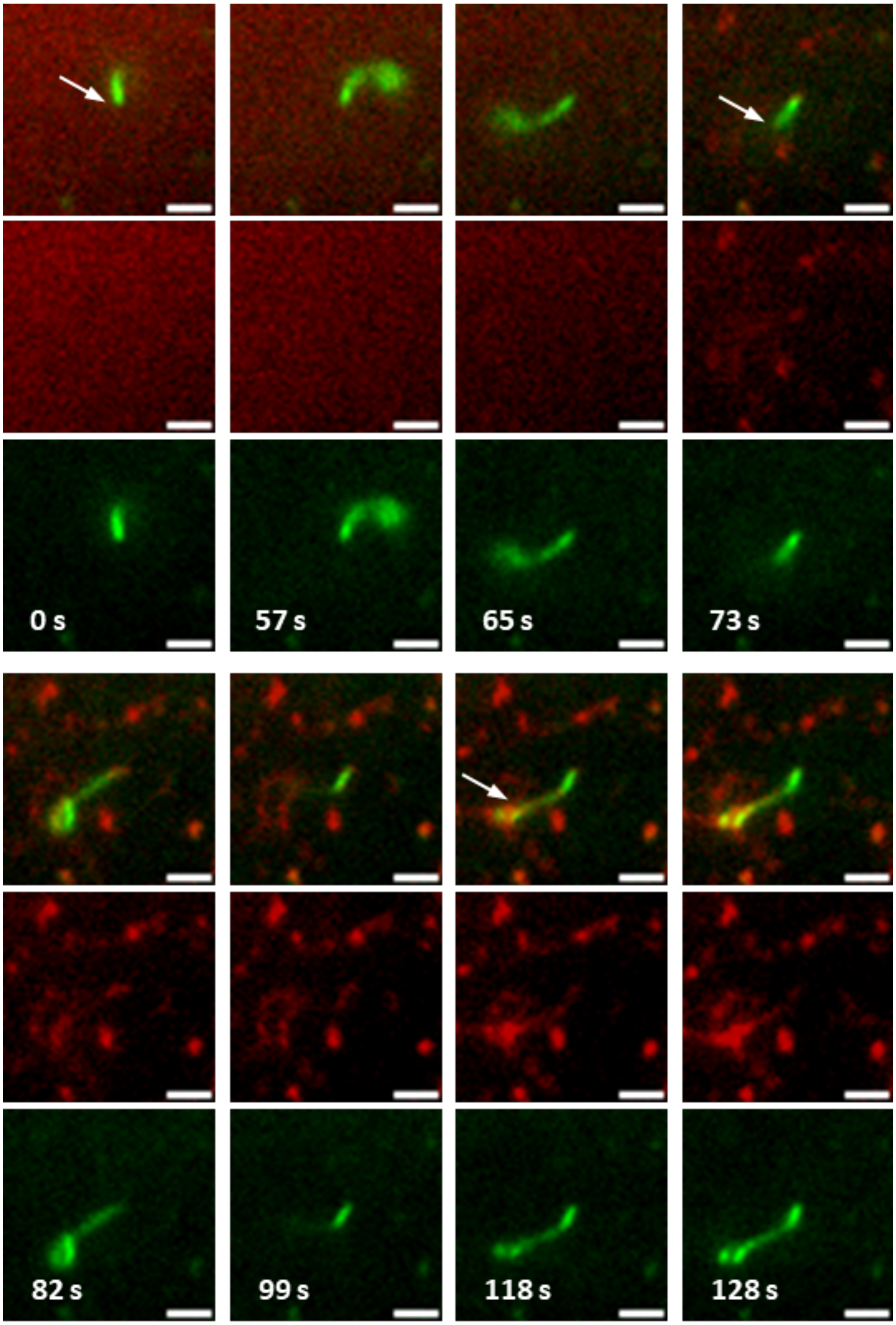

Figure S33. Additional time lapse imaging showing zipping effect. [Receptor] = 0.1 mol %, [BTA-DNA] = 1 %, [BTA-Cy3] = 5%, DNA_3Biotin = 1 eq., $c_{T,BTA}$ = 2.5 μM, scale bar = 2 μm. Red channel = receptor, Green = BTA-Cy3

Note: Images show clustering of homogeneous receptors labelled with Cy5 during anchoring of fibers labelled with Cy3.

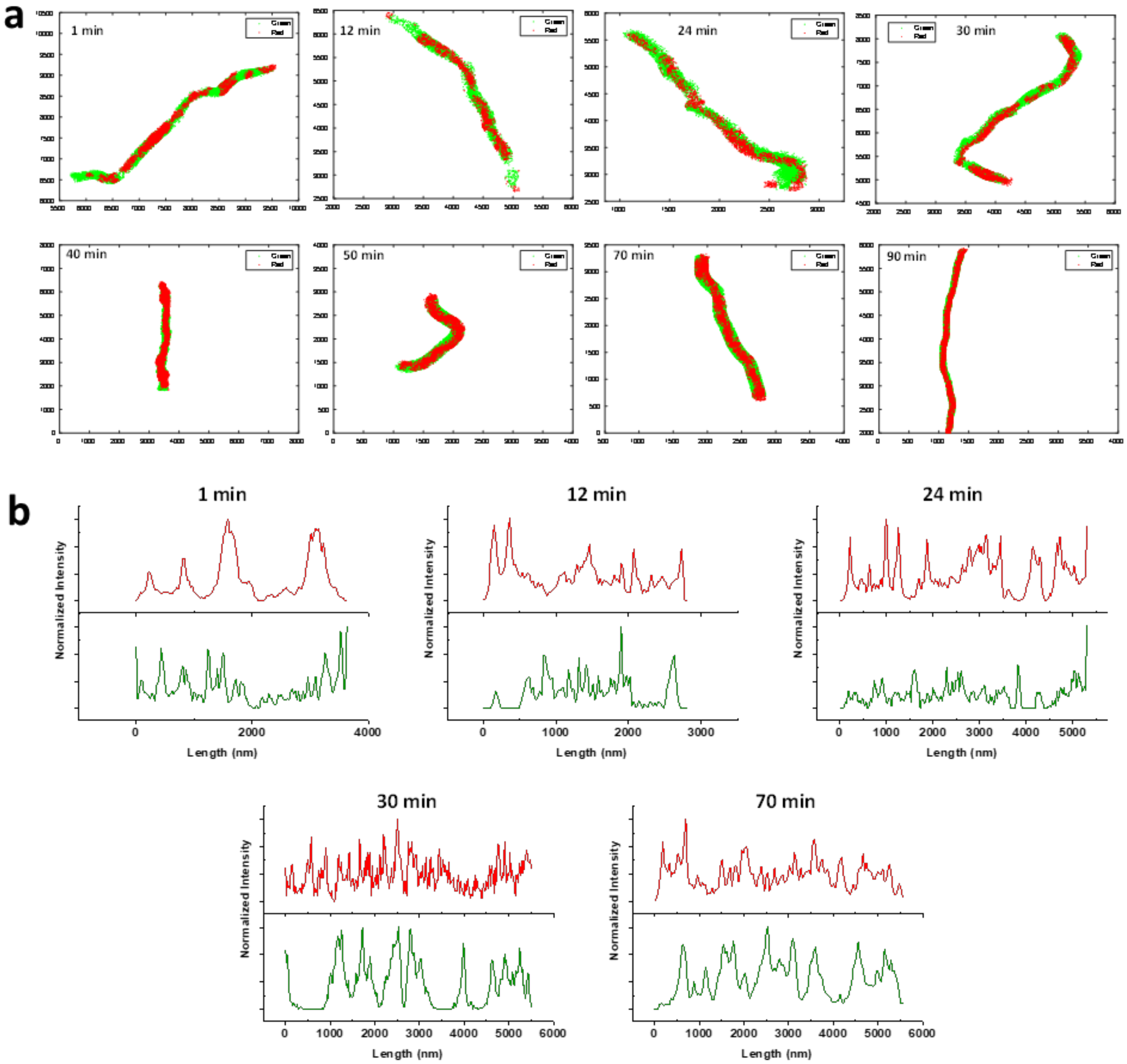

Figure S34. (a) Dynamic exchange of monomers between anchored supramolecular fiber BTA-Bio-Cy3 and added supramolecular polymer BTA-Cy5. Corresponding two channel STORM localization depicting fast, homogeneous exchange of monomers. (b) Corresponding intensity variation throughout polymer backbone depict homogeneous exchange of monomers during dynamic exchange of monomers between anchored supramolecular fiber BTA-Bio-Cy3 and added supramolecular polymer BTA-Cy5 [BTA-Cy3] = [BTA-Cy5] = 5 %, [BTA-Bio] = 1 %, $c_{T,BTA}$ = 2.5 μM.

Generality of concept (BTABa, BTABaCy3 and GD3):

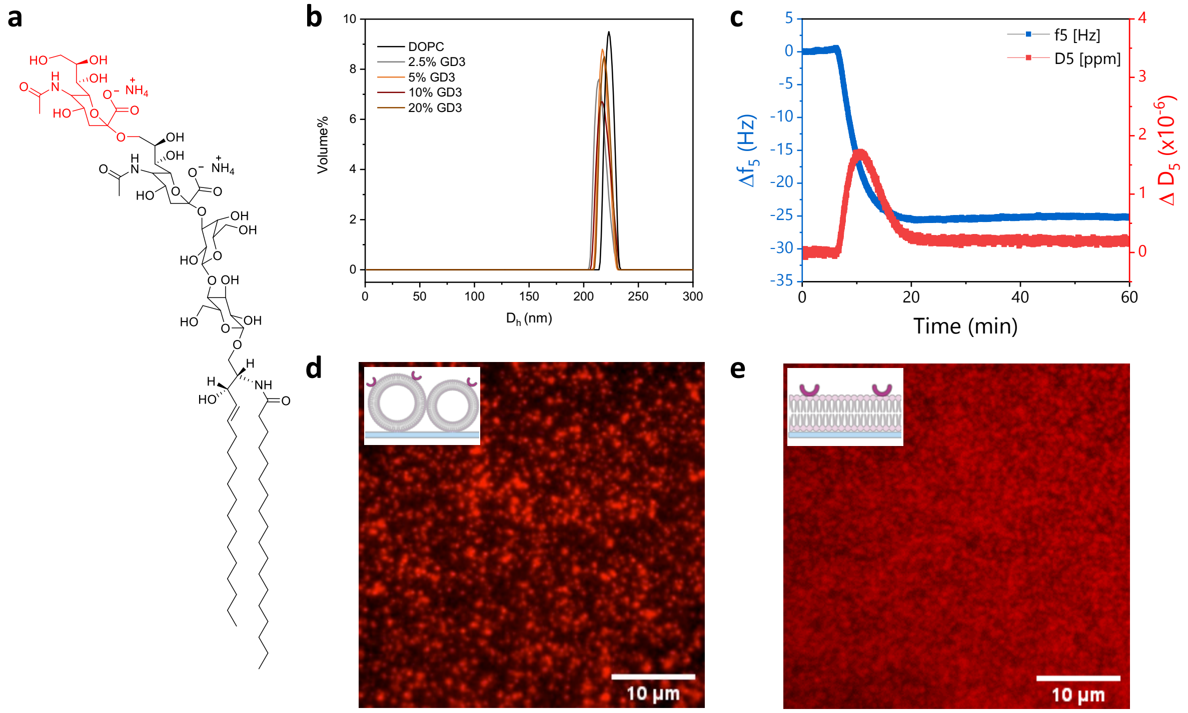

Figure S35. (a) Chemical structure of GD3. (b) Dynamic light scattering of SUVs with varying % of GD3 in DOPC. (c) QCM-D measurement depicting stepwise formation of SLB from SUVs. Th^{e} 5th overtone for frequency shift and change in dissipation is shown. 5% GD3 in DOPC. TIRF image of (d) SUVs and (e) SLB of 10 % GD3 in DOPC in presence of nile red/Texas red-DOPE, $c_{T,BTA}$ = 2.5 µM, $c_{T,lipids}$ = 0.1 mg/mL.

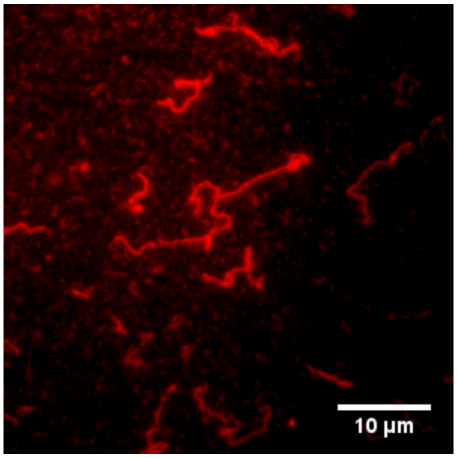

Figure S36. TIRF image of BTA fibers on glass coverslip. 1% BTA-Ba, 5% BTACy3, $c_{T,BTA}$ = 2.5 μM.

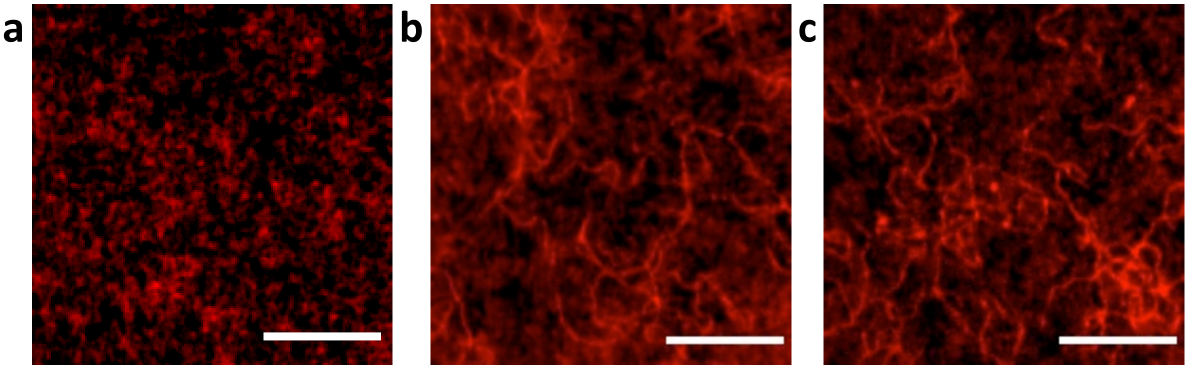

Figure S37. TIRF image of (b) Unbound state [Sialic acid] = 5 %, (c) Partially bound state [Sialic acid] = 10 mol % and (d) Completely bound state [Sialic acid] = 20 mol %. [BTA-Ba] = 1 %, [BTACy5] = 5%, $c_{T,BTA}$ = 2.5 μM, scale bar = 10 μm.

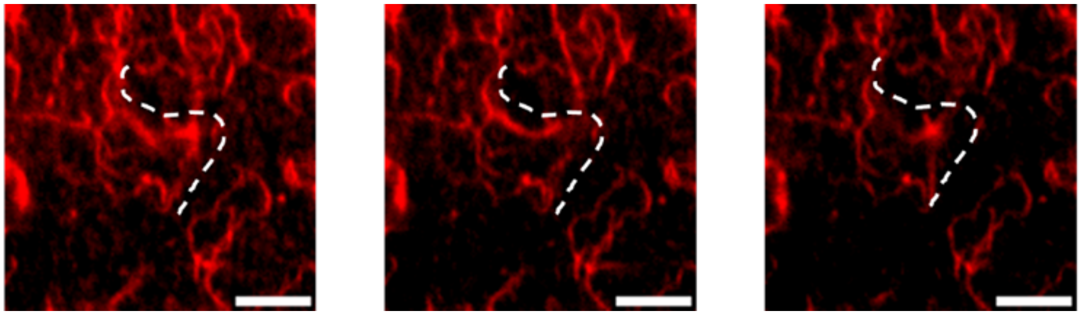

Figure S38. Interaction of BTA fibers with supported lipid bilayers. Schematic representation and TIRF image of (a) interacting fibers (**BTA-Ba** 1%) (b) non-interacting fibers (**BTA-Ba** 0%) on 20% GD3 SLB. (c) Effect of change in % ligand (BTA-Ba) and % receptor (GD3/sialic acid) on anchoring of BTA fibers to SLB. (d) Time lapse imaging of partially bound BTA fibers (BTA-Ba 1% on 10% GD3 SLB). ($c_{BTA,total}$ = 2.5 µM, **BTA-Cy5** 5%, with respect to total BTA concentration, scalebar = 5 µm). The dashed white line is added as a guide to the eye for the moving supramolecular polymer.

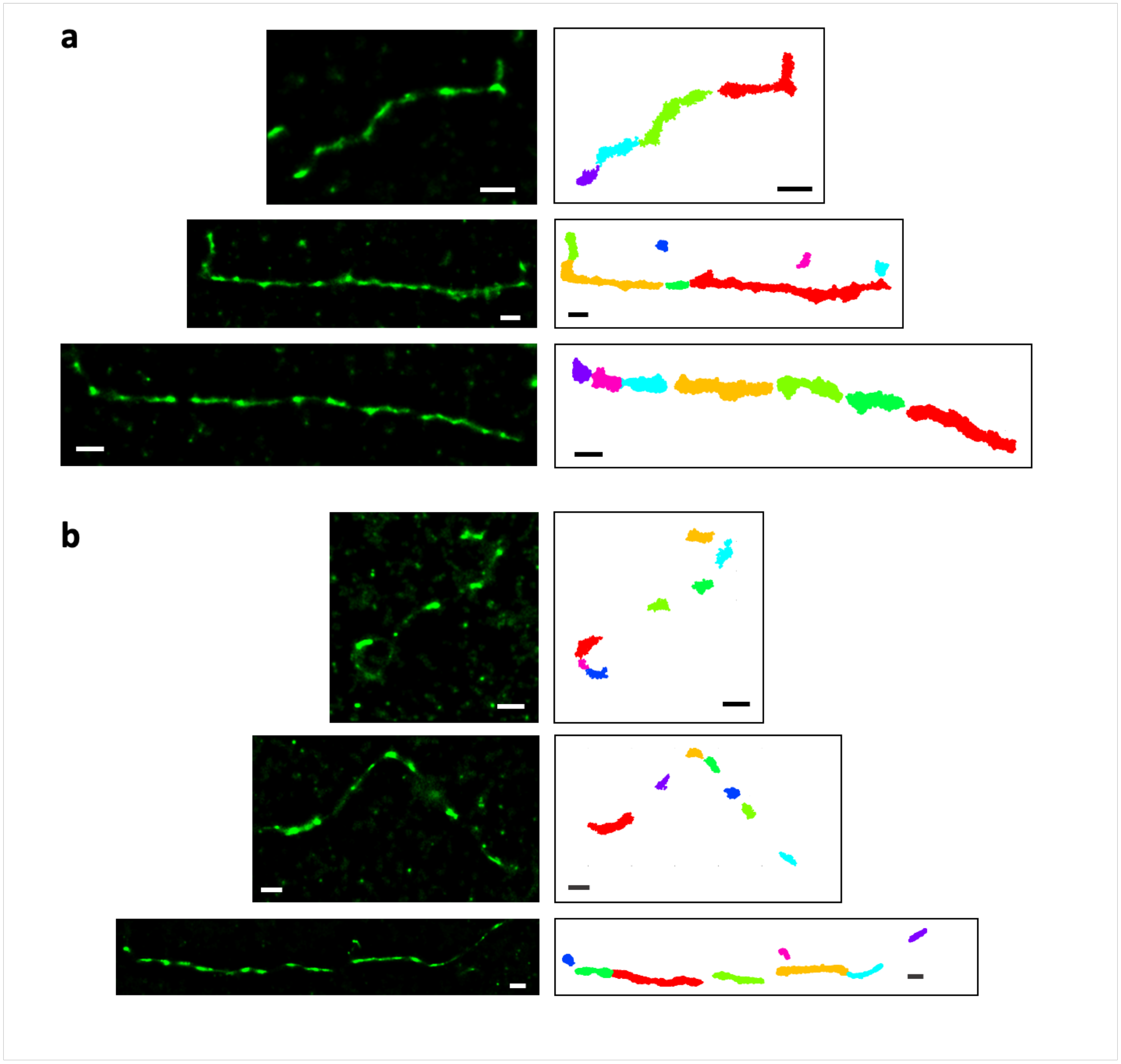

Figure S39. STORM image and corresponding cluster analysis of BTABaCy3 fibers on (a) Glass and (b) GD3 based SLB.

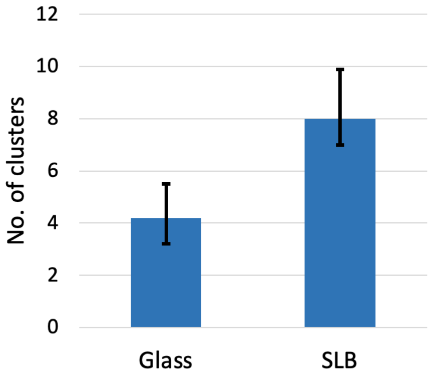

Figure S40. No. of cluster obtained for BTABaCy3 fibers on Glass and GD3-based SLB with n = 3, and r = 10. Standard deviation is of 5 images each.

Receptor mobility (BTABio and SAv)

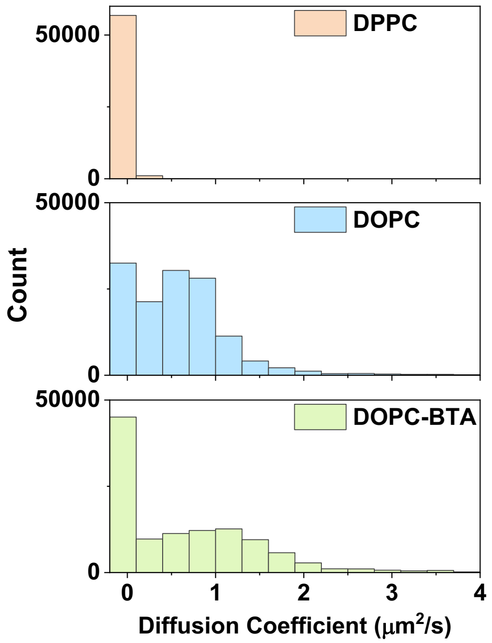

Figure S41. Diffusion coefficient vs. count from SPT-PALM for DPPC (0.1 % DPPC-Bio), DOPC (0.1 % DOPC-Bio) and DOPC-BTA (BTA fibers anchored on 0.1 % DOPC-Bio). $c_{T,BTA}$ = 2.5 μM.

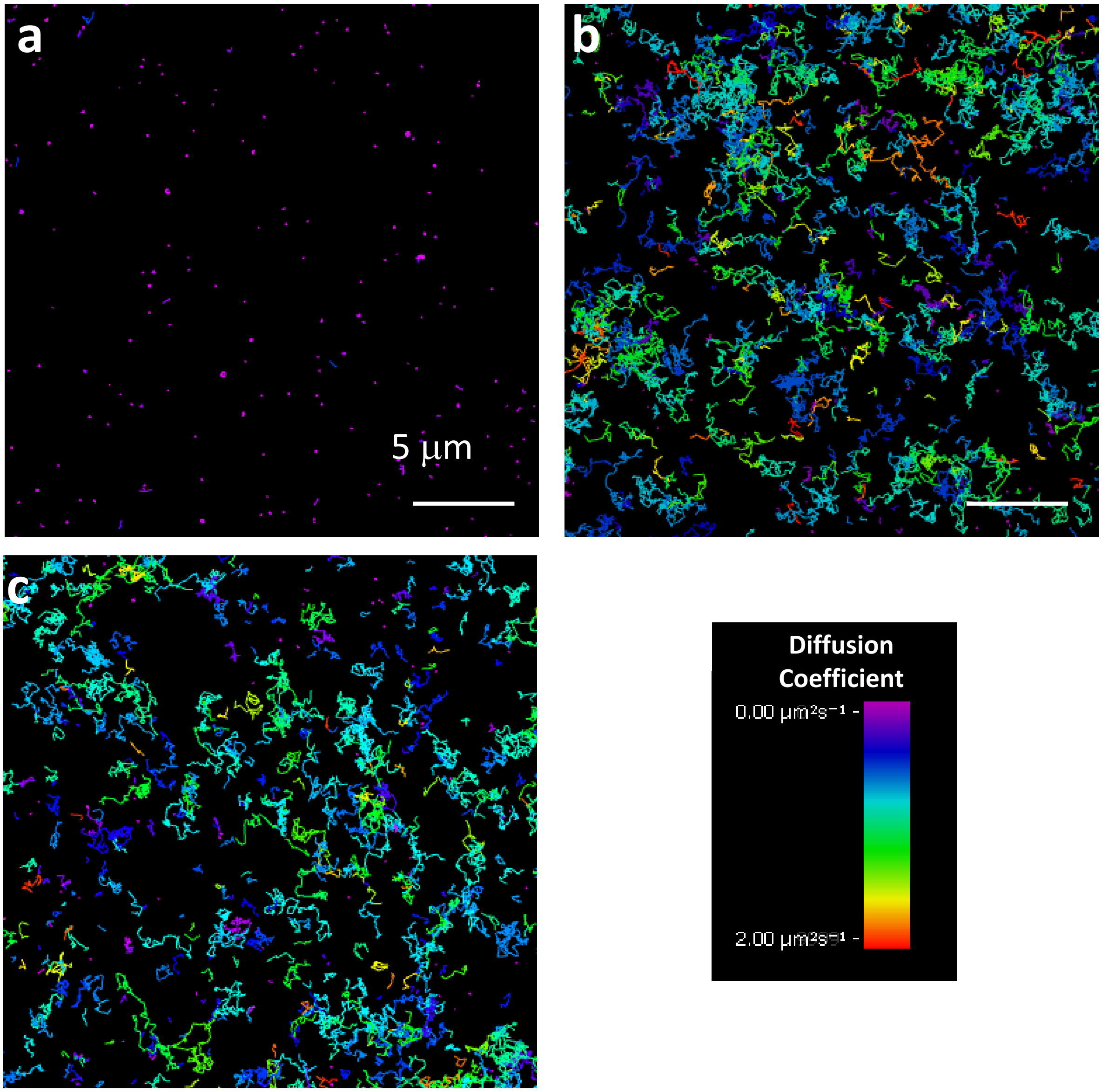

Figure S42. Tracks of receptor SAv from SMT-PALM of (a) DPPC (0.1 % DPPC-Bio), (b) DOPC (0.1 % DOPC-Bio) and (c) DOPC (0.1 % DOPC-Bio) after anchoring of BTA-Bio-Cy3 fibers depicting change in diffusion coefficient of receptors. $c_{T,BTA}$ = 2.5 µM.

References:

1. L. Albertazzi, D. van der Zwaag, C. M. Leenders, R. Fitzner, R. W. van der Hofstad, E. W. Meijer. Probing exchange pathways in one-dimensional aggregates with super-resolution microscopy. *Science* **344**, 491–495 (2014).
2. M. Ester, H.-P. Kriegel, J. Sander, X. Xiaowei. A density-based algorithm for discovering clusters in large spatial databases with noise. In *Proceedings of the Second International Conference on Knowledge Discovery in Databases and Data Mining* (pp. 226–231). Portland, OR: AAAI Press (1996).
3. https://de.mathworks.com/help/stats/dbscan-clustering.html
4. C. M. A. Leenders, L. Albertazzi, T. Mes, M. M. Koenigs, A. R. Palmans, E. W. Meijer. Supramolecular polymerization in water harnessing both hydrophobic effects and hydrogen bond formation. *Chem. Commun.* **49**, 1963–1965 (2013).
5. G. Morgese, B. F. M. de Waal, S. Varela-Aramburu, A. R. A. Palmans, L. Albertazzi, E. W. Meijer. Anchoring supramolecular polymers to human red blood cells by combining dynamic covalent and non-covalent chemistries. *Angew. Chem. Int. Ed.* **132**, 17229–17233 (2020).
6. L. Su, J. Mosquera, M. F. J. Mabesoone, S. M. C. Schoenmakers, C. Muller, M. E. J. Vleugels, S. Dhiman, S. Wijker, A. R. A. Palmans, E. W. Meijer. Dilution-induced gel-sol-gel-sol transitions by competitive supramolecular pathways in water. *Science* **377**, 213–218 (2022).
7. S. P. Wijnands, W. Engelen, R. P. Lafleur, E. W. Meijer, M. Merkx. Controlling protein activity by dynamic recruitment on a supramolecular polymer platform. *Nat. Commun.* **9**, 65 (2018).
8. R. E. Kieltyka, A. C. H. Pape, L. Albertazzi, Y. Nakano, M. M. Bastings, I. K. Voets, P. Y. W. Dankers, E. W. Meijer. Mesoscale modulation of supramolecular ureidopyrimidinone-based poly(ethylene glycol) transient networks in water. *J. Am. Chem. Soc.* **135**, 11159–11164 (2013).
9. P. Y. W. Dankers, T. M. Hermans, T. W. Baughman, Y. Kamikawa, R. E. Kieltyka, M. M. Bastings, H. M. Janssen, N. A. J. M. Sommerdijk, A. Larsen, M. J. A. van Luyn, A. W. Bosman. Hierarchical formation of supramolecular transient networks in water: a modular injectable delivery system. *Adv. Mater.* **24**, 2703–2709 (2012).
10. S. I. Hendrikse, S. P. Wijnands, R. P. Lafleur, M. J. Pouderoijen, H. M. Janssen, P. Y. W. Dankers, E. W. Meijer. Controlling and tuning the dynamic nature of supramolecular polymers in aqueous solutions. *Chem. Commun.* **53**, 2279–2282 (2017).
11. I. de Feijter, O. J. Goor, S. I. Hendrikse, M. Comellas-Aragones, S. H. Söntjens, S. Zaccaria, P. P. Fransen, J. W. Peeters, L. G. Milroy, P. Y. Dankers. Solid-Phase-Based Synthesis of Ureidopyrimidinone–Peptide Conjugates for Supramolecular Biomaterials. *Synlett* **26**, 2707–2713 (2015).
12. F. J. Martinez-Veracoechea, D. Frenkel. Designing super selectivity in multivalent nano-particle binding. *Proc. Natl. Acad. Sci.* **108**, 10963–10968 (2011).
13. M. Crippa, C. Perego, A. L. de Marco, G. M. Pavan. Molecular communications in complex systems of dynamic supramolecular polymers. *Nat. Commun.* **13**, 2162 (2022).
14. A. L. de Marco, D. Bochicchio, A. Gardin, G. Doni, G. M. Pavan. Controlling exchange pathways in dynamic supramolecular polymers by controlling defects. *ACS Nano* **15**, 14229–14241 (2021).
15. D. Bochicchio, M. Salvalaglio, G. M. Pavan. Into the dynamics of a supramolecular polymer at submolecular resolution. *Nat. Commun.* **8**, 147 (2017).
16. D. Bochicchio, G. M. Pavan. From cooperative self-assembly to water-soluble supramolecular polymers using coarse-grained simulations. *ACS Nano* **11**, 1000–1011 (2017).

17. H. C. Andersen. Molecular dynamics simulations at constant pressure and/or temperature. *J. Chem. Phys.* **72**, 2384–2393 (1980).

18. M. J. Abraham, T. Murtola, R. Schulz, S. Páll, J. C. Smith, B. Hess, E. Lindahl. GROMACS: High performance molecular simulations through multi-level parallelism from laptops to supercomputers. *SoftwareX* **1**, 19–25 (2015).

19. The PLUMED Consortium. Promoting transparency and reproducibility in enhanced molecular simulations. *Nat. Methods* **16**, 670–673 (2019).

20. A. Laio, M. Parrinello. Escaping free-energy minima. *Proc. Natl. Acad. Sci.* **99**, 12562–12566 (2002).

21. J. Pfaendtner, M. Bonomi. Efficient sampling of high-dimensional free-energy landscapes with parallel bias metadynamics. *J. Chem. Theory Comput.* **11**, 5062–5067 (2015).

22. P. Tiwary, M. Parrinello. A time-independent free energy estimator for metadynamics. *J. Phys. Chem. B* **119**, 736–742 (2015).

23. L. Kollias, D. C. Cantu, V.-A. Glezakou, R. Rousseau, M. Salvalaglio. On the role of enthalpic and entropic contributions to the conformational free energy landscape of MIL-101 (Cr) secondary building units. *Adv. Theory Simul.* **3**, 2000092 (2020).

24. I. Gimondi, G. A. Tribello, M. Salvalaglio. Building maps in collective variable space. *J. Chem. Phys.* **149**, 104104 (2018).

25. L. Leanza, C. Perego, L. Pesce, M. Salvalaglio, M. von Delius, G. M. Pavan. Into the dynamics of rotaxanes at atomistic resolution. *Chem. Sci.* **14**, 6716–6729 (2023).
